# Help! – you need your hands: Contribution of arm movements on balance performance in healthy individuals: A systematic review with meta-analysis

**DOI:** 10.1371/journal.pone.0323309

**Published:** 2025-05-08

**Authors:** Katharina Borgmann, Thomas Muehlbauer, Mathew W. Hill

**Affiliations:** 1 Division of Movement and Training Sciences/Biomechanics of Sport, University of Duisburg-Essen, Essen, Germany; 2 Center for Physical Activity, Sport and Exercise Sciences, Coventry University, Coventry, United Kingdom; Aichi Prefectural Mikawa Aoitori Medical and Rehabilitation Center for Developmental Disabilities, JAPAN

## Abstract

**Background:**

Emerging evidence highlights that arm movements exert a substantial and functionally relevant contribution on postural control in healthy individuals. However, performance differences between free versus restricted arm movement for different balance categories with varying levels of task difficulty have not been systematically investigated yet.

**Objective:**

The objective of this systematic review and meta-analysis was to characterise, aggregate, and quantify performance discrepancies between free and restricted arm movement conditions for diverging balance categories with varying levels of task difficulty in healthy individuals.

**Methods:**

A systematic search of the literature according to the PRISMA guidelines was performed on the electronic databases PubMed, Web of Science, and SPORTDiscus from their inception date till 1st September 2024. To be applicable for analysis, studies had to report at least one measure of balance performance in healthy individuals. The included studies were coded according to the following criteria: age, sex, status, arm movement conditions, balance test, and test modality. Methodological study quality was assessed using the Appraisal tool for Cross-Sectional Studies. Weighted standardized mean differences (*SMD*) were calculated and classified according to their magnitude.

**Results:**

The literature search identified a total of *N* = 941 records, 25 of which met the inclusion criteria and were analysed in this review. A total of 725 participants (*n* = 331 females) participated in the studies. The free use of arm movement resulted in moderate (static: *SMD* = 0.51, dynamic: *SMD* = 0.66, proactive: *SMD* = 0.52, reactive: *SMD* = 0.50) improvements of balance performance. In addition, the performance enhancements were more pronounced for balance tasks with a high (static: *SMD* = 0.89, dynamic: *SMD* = 1.04) compared to a low (static: *SMD* = 0.20, dynamic: *SMD* = 0.76) difficulty level. Due to a lack of studies, no analysis for measures of proactive and reactive balance was performed.

**Conclusions:**

The findings revealed that the free use of arm movement positively affects several measures of balance performance, and this is effect is more pronounced for balance tasks with a high difficulty level.

## Introduction

Traditional conceptual frameworks have viewed human balance control as a predominantly lower limb function with two distinct muscle synergies, often referred to as *ankle* or *hip* strategies [1]. Mechanically, the ankle strategy – which moves the whole body as single-segment inverted pendulum with counteractive torques at the ankle joint – is typically the default option to maintain quiet stance when balance demands are low (i.e., standing on a wide support surface) [2]. More challenging tasks (i.e., standing on a narrow support surface) often place greater reliance on a hip strategy, which moves the body as a double-segment inverted pendulum with counterphase motion at the ankle and hip [3]. Although upright quiet standing can usually be maintained by ankle and hip strategies during most scenarios [4], movements of the arms are not taken into account by these two control strategies.

During challenging balance situations (e.g., standing or walking along a narrow beam) humans intuitively outstretch their arms in an attempt to increase stability [5]. Emerging evidence has given considerable prominence for the existence of an ‘upper body strategy’ complementing the ankle and hip strategies during static and dynamic balance tasks [6,7]. Empirical support for this strategy is drawn largely from research [8–10] reporting robust improvements in balance performance under *free* (i.e., arms moved freely in all directions) compared to *restricted* (i.e., hands placed on the hips or crossed over the chest) arm movement conditions. In particular, the contribution of arm movements to postural control appears to be more pronounced for balance tasks with a high compared to a low degree of difficulty, e.g., during modified versus normal sensory conditions [11]. Although arm movements appear to exert a substantial and functionally relevant contribution on balance performance in healthy individuals, between-study variability and sample size issues complicate the interpretation of the magnitude of effect. Further evaluation through a systematic review and meta-analysis may reveal unique insights in relation to how arm movements influence balance performance.

Therefore, the purpose of the present systematic review with meta-analysis was to characterise, aggregate, and quantify performance differences between free and restricted arm movement conditions for diverging balance categories with varying levels of task difficulty in healthy individuals. We assumed that the free use of arm movement would lead to improvements in measures of balance and this positive effect would be more pronounced for balance tasks with a high (e.g., standing with eyes closed) compared to a low (e.g., standing with eyes opened) difficulty level.

## Materials and methods

### Literature search

**Fig 1:**
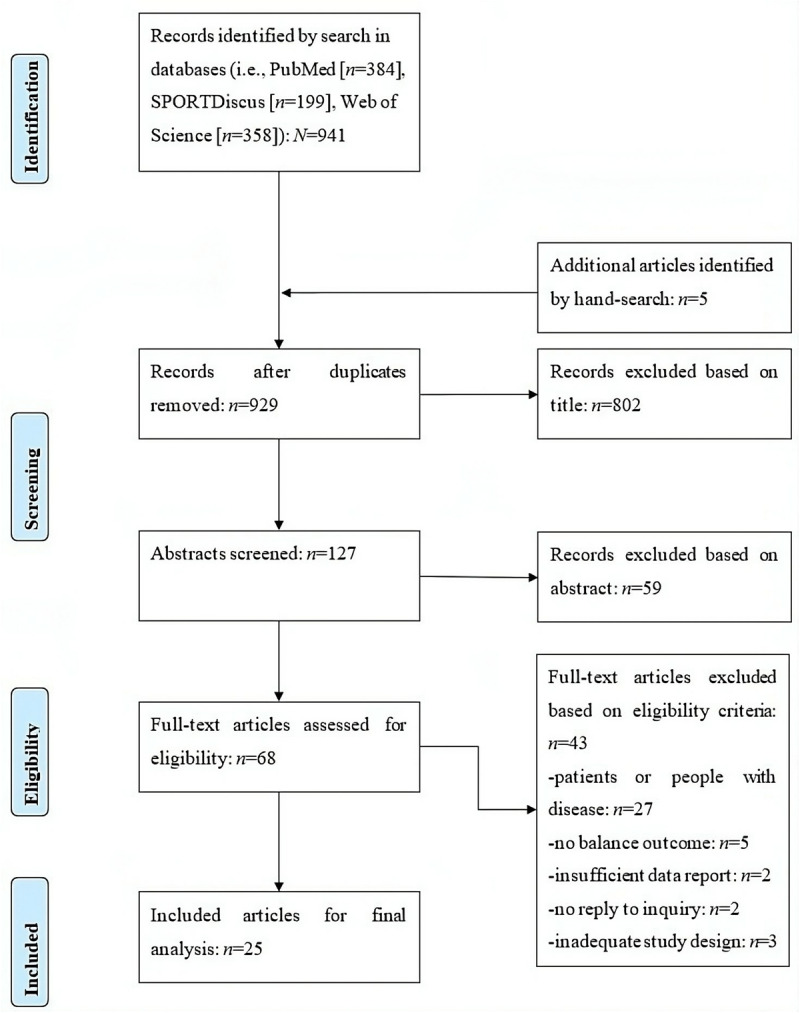
PRISMA flow chart illustrating the different phases of literature search.

The electronic databases PubMed, Web of Science, and SPORTDiscus were searched systematically to identify relevant articles using the following Boolean search strategy: (((“balance performance” OR “postural control” OR “postural balance” OR “postural stability” OR “balance test” OR “balance function” OR “balance ability”) AND (“arm movement” OR “arm position” OR “arm motion” OR “arm swing” OR “upper body” OR “upper extremity”) NOT (patient OR disease OR impairment OR dysfunction OR disability OR pathology))). The search covered the period from their inception until 1st September 2024. Only articles written in English language with full-text access that investigated human species were included. Moreover, reference lists of the included articles were checked for other studies potentially suitable for inclusion. After removing all duplicates, the title and abstract of all articles were screened for eligibility according to the inclusion and exclusion criteria. The full texts of all potentially eligible records were independently assessed by the authors and disagreement was resolved through discussion and consent (S1 Table). The process of literature search, study selection, and reasons for exclusion records are presented in Fig 1 by using the PRISMA flow chart [12]. Further, the PRISMA checklist was used for transparent reporting of systematic reviews and meta-analyses (S2 Table).

### Criteria for study selection

The inclusion and exclusion criteria are summarised in Table 1. To be eligible for inclusion, studies had to meet the following inclusion criteria: (a) participants were healthy; (b) at least one measure of balance (static, dynamic, proactive, or reactive) was assessed; (c) descriptive data (e.g., mean value and standard deviation) for calculating effect sizes were reported; (d) a cross-sectional study design was used. The exclusion criteria were as follows: (a) patients or people with disease; (b) reported data did not include a measure of balance performance or allow the calculation of effect size; (c) study authors did not reply to our inquiries to send original data by email; (d) an intervention study was conducted but no pre-intervention data were reported. Study eligibility was independently assessed by two authors (KB and TM). If both did not reach a consensus concerning study eligibility, the third author (MWH) was asked for clarification.

**Table 1:** Description of the inclusion and exclusion criteria.

Categories	Inclusion criteria	Exclusion criteria
Population	Healthy individuals	Patients, people with musculoskeletal dysfunction, neurological impairment, or orthopaedic pathology
Outcome	At least one descriptive measure of static, dynamic, proactive, or reactive balance	No descriptive measure of balance or the reported data did not allow for calculation of effect size, or the study authors did not respond to our inquiries to send original data
Study design	Cross-sectional studies	Intervention studies not reporting pre-intervention data

### Coding of studies

**Fig 2:**
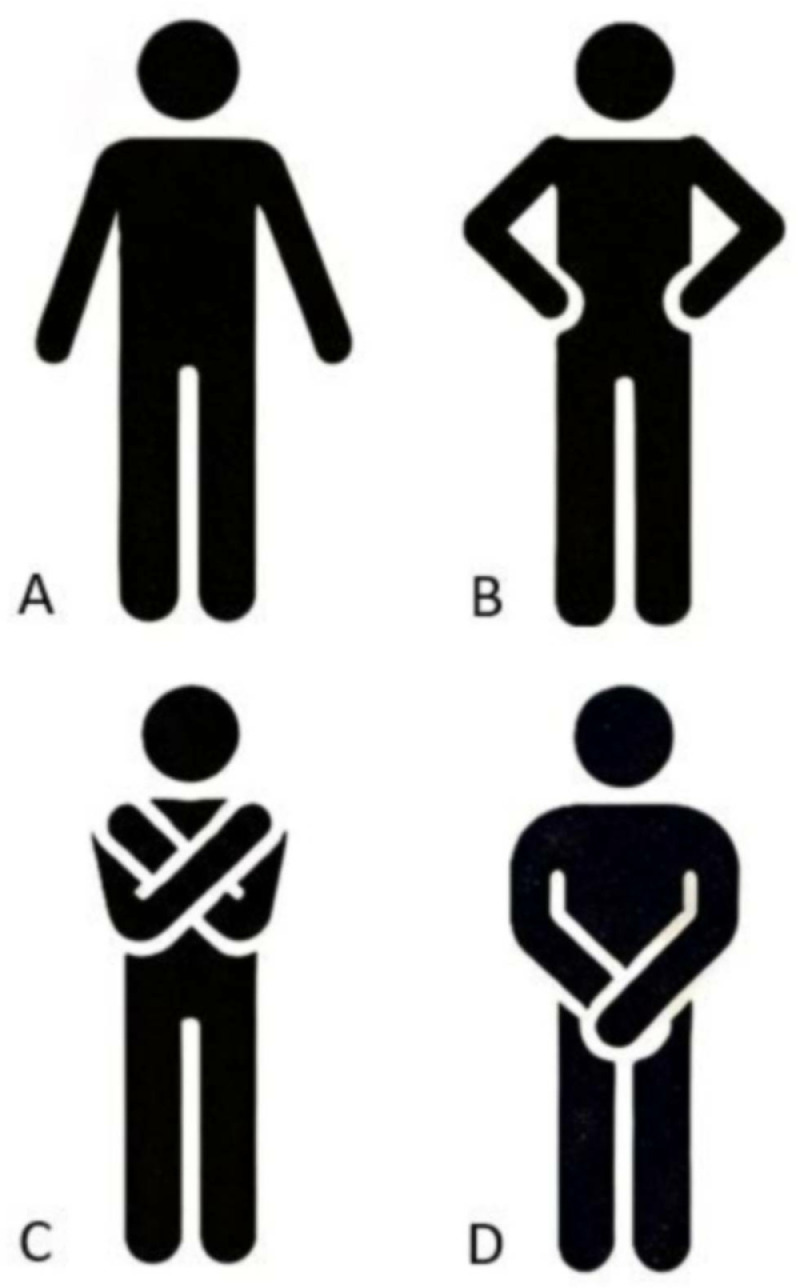
Schematic description of the different arm movement conditions. A) Arms free (i.e., arms free to move); B) Arms restricted (i.e., hands placed on the hips); C) Arms restricted (i.e., arms crossed over the chest); D) Arms restricted (i.e., hands clasped in front of the body at the waist).

The included studies were coded for the following criteria: authors, year of publication, number of participants, sex, chronological age, arm movement conditions, balance assessment, and test modality. Arm movement conditions in free (i.e., arms free to move) and restricted (i.e., hands placed on the hips, hands crossed over the chest or hands clasped in front of the body at the waist) arm movements (Fig 2 A–D). As suggested by Shumway-Cook and Woollacott [13], assessment of balance performance was classified into the following four categories: *static* (i.e., maintenance of a steady position while standing), *dynamic* (i.e., maintenance of a steady position while ambulation), *proactive* (i.e., anticipation of an expected postural disturbance), and *reactive* (i.e., compensation of an unexpected postural disturbance). Because some studies reported several outcomes within one balance domain, we *a priori* defined preferred and alternative outcomes for each domain to reduce heterogeneity (Table 2). In terms of static balance, highest priority was given to centre of pressure (CoP) amplitude during standing, while step width during walking was used with reference to dynamic balance. Regarding proactive balance, highest relevance was given to the composite score in the Y Balance Test–Lower Quarter (YBT–LQ), while step width during perturbed walking were defined as most representative for reactive balance.

**Table 2:** Overview of the preferred and alternative outcome by balance domain.

Balance domain	Preferred outcome	Alternative outcome
Static balance	CoP amplitude (*n* = 4)	CoP displacement (*n* = 1); stance time (*n* = 3)
Dynamic balance	Step width (*n* = 3)	Walking speed (*n* = 2); walking time (*n* = 2); step number (*n* = 3), stride duration (*n* = 1); torque amplitude (*n* = 1)
Proactive balance	YBT–LQ composite score (*n* = 9)	*N/A*
Reactive balance	Step width (*n* = 3)	Recovery or transfer time (*n* = 2); DPSI (*n* = 2)

Note. The figure in brackets indicates the number of studies that used the respective outcome. *CoP* centre or pressure, *N/A* not applicable, *YBT–LQ* Y Balance Test–Lower Quarter.

### Appraisal of methodological study quality

For the assessment of methodological study quality, we used the Appraisal tool for Cross-Sectional Studies [14]. The tool consists of 20 questions addressing study quality, study design, and risk of bias, which have to be answered with “yes”, “no”, or “do not know”. Seven questions (1, 4, 10, 11, 12, 16, and 18) refer to the quality of reporting and another seven questions (2, 3, 5, 8, 17, 19, and 20) to the quality of the study design. Further six questions (6, 7, 9, 13, 14, and 15) relate to a possible risk of bias. However, three questions (7, 13, 14) were excluded from our analysis because they address aspects (e.g., potential non-responders) that were not applicable for the included studies. The assessment of methodological study quality was independently performed by two authors (KB and TM). If the respective authors did not obtain a consensus, the third author (MWH) was asked to clarify.

### Statistical analyses

To quantify differences in balance performance between free and restricted arm movement condition, the within-subject weighted standardised mean difference was calculated as *SMD* = (free arm movement mean value – restricted arm movement mean value)/free arm movement standard deviation using Review Manager version 5.4.1 [15]. Depending on the balance outcome, the *SMD* value can be negative or positive for the same arm movement condition. For example, a decrease in CoP displacement under free arm movement condition during single-leg stance would yield a negative *SMD* value, whereas a longer time in balance during free compared to restricted arm movement condition would result in a positive *SMD* value, although both values represent better balance performance. Thus, *SMD* values with a positive sign are always indicative for a better balance performance in favour of the free arm movement condition, while *SMD* values with a negative sign indicate better balance performance in favour of the restricted arm movement condition. In accordance to Cohen [16], the *SMD* value was classified as small (0 ≤ 0.49), moderate (0.50 ≤ 0.79), or large (≥ 0.80). In addition, heterogeneity (*I*^2^) between studies was computed by using the formula provided by Deeks et al. [17]: *I*^2^ = (*Q* – *df*/*Q*) ▪ 100% where *Q* is the *Chi*^*2*^ statistics and *df* represents the degrees of freedom [18]. In agreement with Deeks et al. [17], the *I*^2^ value was classified as trivial (0 ≤ 40%), moderate (30 ≤ 60%), substantial (50 ≤ 90%), or considerable (75 ≤ 100%). To test whether the heterogeneity between studies was influenced by certain studies with extreme effect sizes, we additionally conducted an influence analysis using the Leave-One-Out method [19]. Further, Egger’s test was used as a method to check for publication bias [20].

## Results

Fig 1 illustrates the process of the systematic electronic literature search, which identified a total of 941 records. After removing duplicates, screening titles, reading abstracts, and excluding ineligible articles, 25 studies remained and were included in our meta-analysis.

### Characteristics of the included studies

Table 3 shows the characteristics of the 25 included studies. Eight [10,11,21–26], thirteen [6,7,9,22–24,27–33], nine [6,8,10,22–24,34–36], and six [6,28,29,37–39] studies reported variables of static, dynamic, proactive, and reactive balance, respectively (S3 Table). A total of 725 subjects (*n *= 331 females) participated in the 25 studies, with six [6,22–24,35,39], eighteen [7–11,21,22,25,26,28–31,33,34,36–38], and five [11,26,27,32,33] studies investigating youth, young and older adults, respectively.

**Table 3:** Chronological overview of the included studies investigating the contribution of arm movements on balance performance in healthy individuals.

References	No. of subjects; gender; age (range or mean ± SD); status	Arm movement conditions	Assessment (outcome [unit]) by balance domain	Test modality
Stephenson et al. [27]	10; F (4), M (6); 62 ± 6 years; healthy	Arms *free* (arms hang freely by the sides of the body) vs. *restricted* (holding onto handles that were fixed in place)	*Dynamic balance:* Walking at slow speed of 0.70 ± 0.23 m/s on a treadmill (stride duration [s], stride length [m], joint angles [°], muscle activation [%])	*N/A*
Milosevic et al. [9]	10; F (5), M (5); 52.9 ± 1.8 years; healthy	Arms *free* (arms free to move) vs. *restricted* (arms flat against the body)	*Dynamic balance:* Maximal Step Length Test (step time [s], step length [cm], step speed [cm/s]); Step Test (steps [no]); Timed Up and Go Test (time [s]); Walk along an elliptical line (time [s])	*N/A*
Patel et al. [21]	12; F (5); 28 years, M (7); 27 years; healthy	Arms *free* (outstretched laterally approx. at shoulder level) vs. *restricted* (arms down to the side)	*Static balance:* Tandem stance on a force plate (sway amplitude [cm], sway path length [mm], sway velocity [cm/s])	Standing with eyes open and closed
Cheng et al. [37]	12; M; 23.9 ± 1.9 years; healthy	Arms *free* (arms free to move) vs. *restricted* (folding the arms on the front of the chest)	*Reactive balance:* Single-step balance recovery after a sudden release from a forward-lean posture (transfer time [ms], step time [ms], total balance time [ms], step length [%BH], step velocity [m/s], maximum anterior/vertical CoM displacement [%BH])	Release from three forward-lean angles (i.e., 12.5°, 15.0°, 17.5°)
Cheng et al. [38]	12; M; 23.9 ± 1.9 years; healthy	Arms *free* (arms free to move) vs. *restricted* (folding the arms on the front of the chest)	*Reactive balance:* Single-step balance recovery after a sudden release from a forward-lean posture (recovery time [ms], success rate [%], vertical and horizontal ground reaction force [%BW], joint angles [°])	Release from three forward-lean angles (i.e., 7.5°, 10.0°, 12.5°)
Hébert-Losier [8]	46; F (23), M (23); 25.7 ± 4.6 years; healthy	Arms *free* (arms free to move) vs. *restricted* (hands on the hips)	*Proactive balance:* YBT–LQ (reach distance [%LL], composite score [%LL])	*N/A*
Boström et al. [7]	22; M; 24.3 ± 3.0 years; healthy	Arms *free 1* (arms free to move) vs. *free 2* (outstretched perpendicularly in the frontal plane) vs. *restricted* (hands on the thighs)	*Dynamic balance:* Beam Walking Forward Test (torque amplitude [kNm/kg▪m], torque variation [kNm/kg▪m])	Walking over different beam widths (i.e., 6.0, 4.5, 3.0 cm)
Hill et al. [6]	29; F (15), M (14); 10.6 ± 0.5 years; healthy	Arms *free* (arms free to move) vs. *restricted* (arms placed flat across the chest touching the contralateral shoulder)	*Dynamic balance:* Tandem Beam Walking Test (time [s]) *Proactive balance:* YBT–LQ (reach distance [%LL], composite score [%LL]) *Reactive balance:* Anterior Jump-Landing Task on a force plate (DPSI)	*N/A*
Objero et al. [10]	20; F (10), M (10); 20.7 ± 1.3 years; healthy	Arms *free* (arms free to move) vs. *restricted* (arms placed flat across the chest touching the contralateral shoulder)	*Static balance:* Quiet Stance Test on a force plate (CoP path length [cm], CoP displacement [cm]) *Proactive balance:* YBT–LQ (reach distance [%LL], composite score [%LL])	Bipedal, tandem, and unipedal stance on firm and foam surface
Gholizadeh et al. [28]	15; F (7), M (8); 23.4 ± 2.8 years; healthy	Arms *free* (arms free to move) vs. *restricted* (arms were bound at their sides while walking, across the midpoint of their forearms) vs. *released* (walk without arm swing unless it was necessary to recover balance)	*Dynamic balance:* Unperturbed symmetric and asymmetric walking on a split belt treadmill (step width [cm], stance time [s], WBAM [m/s], peak trunk angular velocity [°/s], CoM range [cm]) *Reactive balance:* Perturbed symmetric and asymmetric walking on a split belt treadmill (step width [cm], stance time [s], WBAM [m/s], peak trunk angular velocity [°/s], CoM range [cm])	Symmetric (treadmill speed: 1.2 m/s) and asymmetric (treadmill speed, left leg: 1.2 m/s; treadmill speed, right leg: 0.96 m/s) walking
Gholizadeh et al. [29]	15; F (7), M (8); 23.4 ± 2.8 years; healthy	Arms *free* (arms free to move) vs. *restricted* (arms were bound at their sides while walking, across the midpoint of their forearms) vs. *released* (walk without arm swing unless it was necessary to recover balance)	*Dynamic balance:* Unperturbed symmetric and asymmetric walking on a split belt treadmill (step width [cm], stance time [s], WBAM [m/s], peak trunk angular velocity [°/s], CoM range [cm]) *Reactive balance:* Perturbed symmetric and asymmetric walking on a split belt treadmill (step width [cm], stance time [s], WBAM [m/s], peak trunk angular velocity [°/s], CoM range [cm])	Symmetric (treadmill speed: 1.2 m/s) and asymmetric (treadmill speed, left leg: 1.2 m/s; treadmill speed, right leg: 0.96 m/s) walking
Siragy et al. [30]	15; F (7), M (8); 23.4 ± 2.8 years; healthy	Arms *free 1* (participants’ natural arm motion) vs. *free 2* (arms actively swinging to shoulder height) vs. *restricted* (arms held alongside the thighs)	*Dynamic balance:* Unperturbed symmetric and asymmetric walking on a split belt treadmill (step width [cm], step length [cm], step time [ms], WBAM [cm/s], trunk angular velocity [°/s], trunk linear velocity [cm/s])	Symmetric (treadmill speed: 1.2 m/s) and asymmetric (treadmill speed, left leg: 1.2 m/s; treadmill speed, right leg: 0.96 m/s) walking
Mezher et al. [31]	15; F (7), M (8); 23.4 ± 2.8 years; healthy	Arms *free 1* (participants’ natural arm motion) vs. *free 2* (arms actively swinging to shoulder height) vs. *restricted* (arms held alongside the thighs)	*Dynamic balance:* Walking at a speed of 1.2 m/s on a dual-belt treadmill (step width [cm], step length [cm], step time [ms], margin of stability [cm])	Walking on regular or rocky surface
Wdowski et al. [39]	18; M; 10.1 ± 1.6 years; healthy	Arms *free* (arms free to move) vs. *restricted* (arms placed flat across the chest touching the contralateral shoulder)	*Reactive balance:* Anterior and Lateral Jump-Landing Task on a force plate (DPSI, ROM [°], joint position [°], joint position variability [°])	*N/A*
da Silva Costa et al. [32]	20; F (14), M (6); 69 ± 4 years; healthy	Arms *free* (arms free to move) vs. *restricted 1* (arms akimbo) vs*. restricted 2* (arms crossed over chest)	*Dynamic balance:* Beam Walking Forward Test (distance [m], step speed [m/s], RMS of the trunk acceleration [°/s^2^], RMS of CoM displacement [m]) at preferred speed	Walking over different beam widths (i.e., 10.0, 8.0, 6.0 cm) and without and with a concurrent cognitive task (i.e., serial 3 subtractions)
Muehlbauer et al. [22]	111; F (51), M (60); 11–28 years; healthy	Arms *free* (arms free to move) vs. *restricted* (hands placed on the hips)	*Static balance:* Timed Unipedal Stance Test (time [s]) *Dynamic balance:* Beam Walking Backward Test (steps [no.]) *Proactive balance:* YBT–LQ (reach distance [%LL], composite score [%LL])	*Static balance:* Standing with eyes open and closed on firm and foam ground *Dynamic balance:* Walking over different beam widths (i.e., 6.0, 4.5, 3.0 cm)
	40; F (22), M (18); 11.5 ± 0.6 years; healthy			
	30; F (15), M (15); 14.0 ± 1.1 years; healthy			
	41, F (14), M (27); 24.7 ± 3.0 years; healthy			
Muehlbauer et al. [23]	18; F; 10.9 ± 0.9 years; trained (gymnasts) 18; F; 11.3 ± 0.5 years; untrained	Arms *free* (arms free to move) vs. *restricted* (hands placed on the hips)	*Static balance:* Timed Unipedal Stance Test (time [s]) *Dynamic balance:* Beam Walking Backward Test (steps [no.]) *Proactive balance:* YBT–LQ (reach distance [%LL], composite score [%LL])	*Static balance:* Standing with eyes open and closed on firm and foam ground *Dynamic balance:* Walking over different beam widths (i.e., 6.0, 4.5, 3.0 cm)
Muehlbauer et al. [24]	22; F; 11.5 ± 0.6 years; healthy 18; M; 11.5 ± 0.6 years; healthy	Arms *free* (arms free to move) vs. *restricted* (hands placed on the hips)	*Static balance:* Timed Unipedal Stance Test (time [s]) *Dynamic balance:* Beam Walking Backward Test (steps [no.]) *Proactive balance:* YBT-LQ (reach distance [%LL], composite score [%LL])	*Static balance:* Standing with eyes open and closed on firm and foam ground *Dynamic balance:* Walking over different beam widths (i.e., 6.0, 4.5, 3.0 cm)
Sogut et al. [34]	51; F (21), M (30); 22.7 ± 1.9 years; healthy and physically active	Arms *free* (arms free to move) vs. *restricted* (hands on the hips)	*Proactive balance:* YBT-LQ (reach distance [%LL], composite score [%LL])	*N/A*
da Silva Costa et al. [33]	17; F (6), M (11); 24 ± 3 years; healthy 14; F (11), M (3); 69 ± 4 years; healthy	Arms *free* (arms free to move) vs. *restricted* (arms crossed over chest)	*Dynamic balance:* Beam Walking Forward Test (distance [m], step speed [m/s], margin of stability [m]) at preferred speed	Walking without and with a concurrent cognitive task (i.e., serial 3 subtractions)
Hill et al. [25]	30; F (12), M (18); 22.0 ± 4.0 years; healthy	Arms *free* (arms free to move) vs. *restricted* (hands clasped in front of the body at the waist)	*Static balance:* Tandem stance on a force plate (CoP amplitude [mm], CoP frequency [Hz])	Standing on ground level (no threat) and 80 cm above ground (threat)
Johnson et al. [26]	15; F (7), M (8); 21.3 ± 4.2 years; healthy 15; F (7), M (8); 73.3 ± 5.0 years; healthy	Arms *free* (arms free to move) vs. *restricted* (hands clasped in front of the body)	*Static balance:* Bipedal, semi-tandem, and tandem stance on a force plate (CoP amplitude [cm], CoP frequency [Hz])	Bipedal, semi-tandem, and tandem stance
Johnson et al. [11]	15; F (7), M (8); 21.3 ± 4.2 years; healthy 15; F (7), M (8); 73.3 ± 5.0 years; healthy	Arms *free* (arms free to move) vs. *restricted* (hands clasped in front of the body)	*Static balance:* Bipedal, stance on a force plate (CoP range [cm], CoP frequency [Hz])	Standing with eyes open and closed on firm and foam ground
Borgmann et al. [35]	43; F (19), M (24); 12.8 ± 1.9 years; healthy	Arms *free* (arms free to move) vs. *restricted* (hands on the hips)	*Proactive balance:* YBT-LQ (reach distance [%LL], composite score [%LL])	Non-fatigued versus fatigued condition
Borgmann et al. [36]	52; F (21), M (31); 22.6 ± 1.6 years; healthy	Arms *free* (arms free to move) vs. *restricted* (hands on the hips)	*Proactive balance:* YBT-LQ (reach distance [%LL], composite score [%LL])	Non-fatigued versus fatigued condition

*BH* body height, *BW* body weight, *CoM* centre of mass, *CoP* centre of pressure, *DPSI* dynamic postural stability index, *F* female, *LL* leg length, *M* male, *N/A* not applicable, *RMS* root mean square, *WBAM* whole body angular momentum, *YBT–LQ* Y Balance Test–Lower Quarter

### Contribution of arm movement on static balance performance

**Fig 3:**
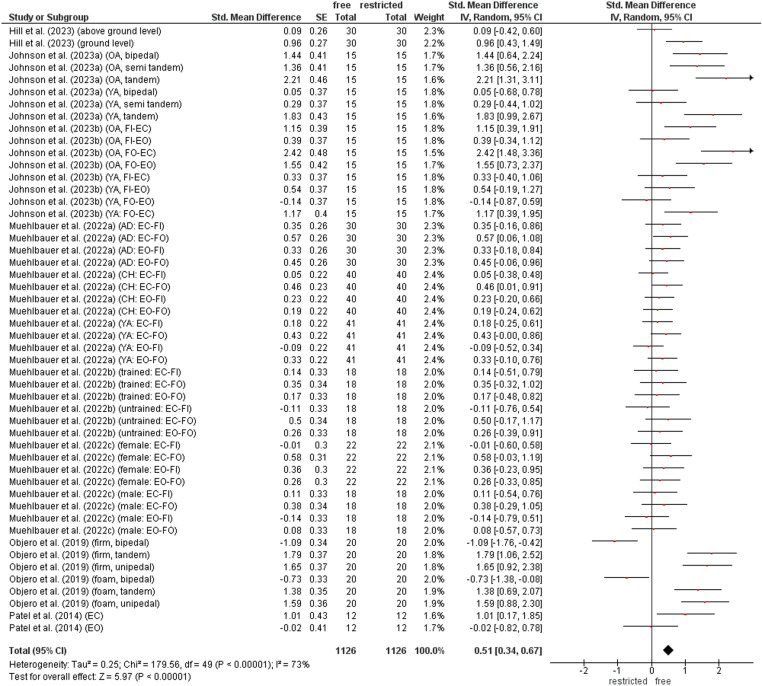
Differences in measures of static balance by arm contribution (i.e., restricted vs. free arm movement condition). *CI* confidence interval, *df* degrees of freedom, SE standard error, *IV* inverse variance.

The comparison of static balance performance between restricted and free arm movement condition is shown in Fig 3. The weighted mean *SMD* value amounted to 0.51 (8 studies, 50 comparisons) indicating a moderate effect in favour of the free arm movement condition. Heterogeneity between studies was substantial (*I*^2^ = 73%).

### Contribution of arm movement on dynamic balance performance

**Fig 4:**
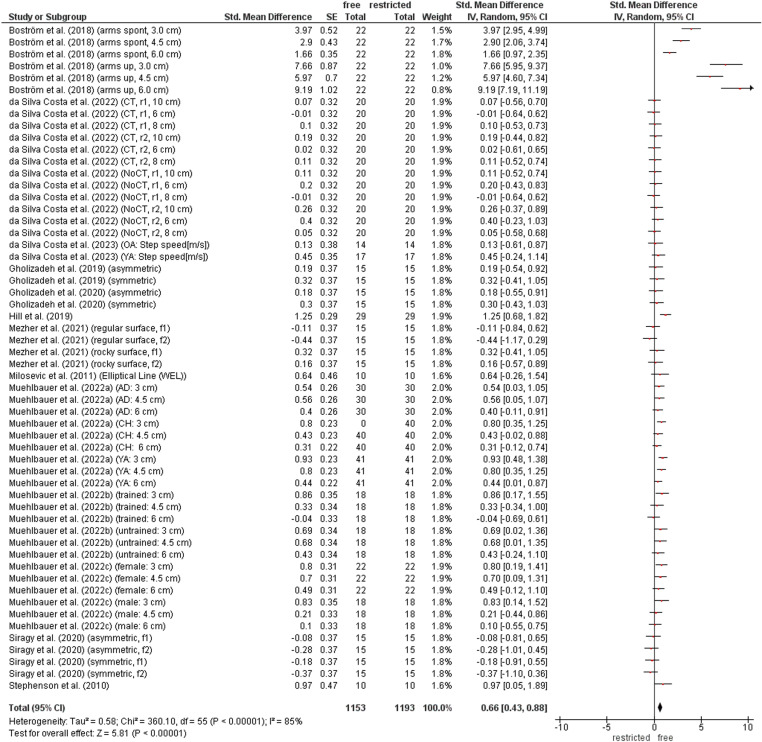
Differences in measures of dynamic balance by arm contribution (i.e., free vs. restricted arm movement condition). *CI* confidence interval, *df* degrees of freedom, SE standard error, *IV* inverse variance.

Fig 4 illustrates the comparison of dynamic balance performance between restricted and free arm movement condition. The weighted mean *SMD* value amounted to 0.66 (13 studies, 56 comparisons) that is indicative for a moderate effect in favour of the free arm movement condition. Heterogeneity between studies ranged from substantial to considerable (*I*^2^ = 85%).

### Contribution of arm movement on proactive balance performance

**Fig 5:**
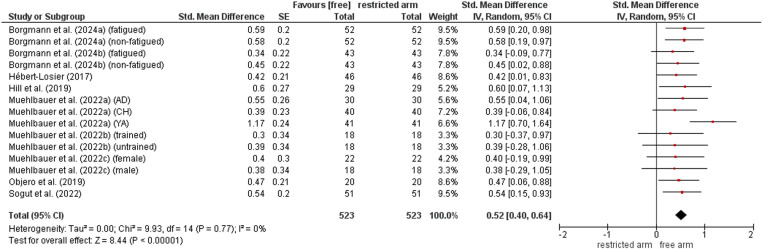
Differences in measures of proactive balance by arm contribution (i.e., free vs. restricted arm movement condition). *CI* confidence interval, *df* degrees of freedom, SE standard error, *IV* inverse variance.

The comparison of proactive balance performance between restricted and free arm movement condition is shown in Fig 5. The weighted mean *SMD* value amounted to 0.52 (9 studies, 15 comparisons) indicating a moderate effect in favour of the free arm movement condition. Heterogeneity between studies was trivial (*I*^2^ = 0%).

### Contribution of arm movement on reactive balance performance

**Fig 6:**
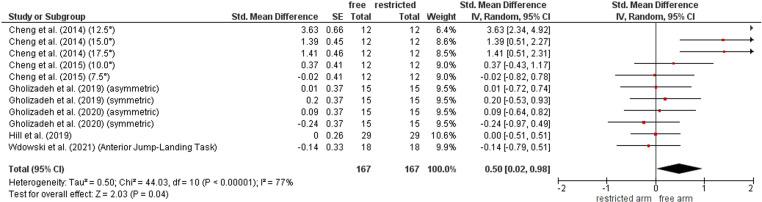
Differences in measures of reactive balance by arm contribution (i.e., free vs. restricted arm movement condition). *CI* confidence interval, *df* degrees of freedom, SE standard error, *IV* inverse variance.

Fig 6 illustrates the comparison of reactive balance performance between restricted and free arm movement condition. The weighted mean *SMD* value amounted to 0.50 (6 studies, 11 comparisons) that is indicative for a moderate effect in favour of the free arm movement condition. Heterogeneity between studies ranged from substantial to considerable (*I*^2^ = 77%).

### Methodological quality of the included studies

The assessment of methodological study quality revealed that most included studies met the criteria for study quality, study design, and risk of bias (S4 Table). Precisely, all included studies fulfilled ≥ 4 out of 7 criteria regarding quality of reporting and study design. In addition, all included studies fulfilled ≥ 2 out of 3 criteria with respect to risk of bias. The categorization in different balance domains resulted in substantial heterogeneity among the included studies. To address this, subgroup analyses were conducted concerning age and task difficulty for the static and dynamic balance. However, a differentiation by age and task difficulty was not feasible for the reactive and proactive balance data due to the limited number of available studies. Age-specific subgroup analyses of the static balance data revealed a considerable reduction in heterogeneity for children (*I*² = 0%) but not for young (*I*² = 80%) and older (*I*² = 62%) adults. The subsequently performed Leave-One-Out method did not lead to notable changes in heterogeneity or effect size for young adults. However, for older adults, the exclusion of one comparison (i.e., bipedal stance with eyes open on firm ground) conducted in the work of Johnson et al. [11] reduced heterogeneity to trivial (23%) but did not change the *SMD* value (data not shown). The analysis of dynamic balance data revealed a comparable pattern. Age-specific subgroup analyses of the dynamic balance data showed a considerable reduction in heterogeneity for children (*I*² = 16%) and older adults (*I*² = 0%) but not for young adults (I² = 93%). The Leave-One-Out method revealed notable changes in heterogeneity and effect size. Specifically, the exclusion of the study by Boström et al. [7] reduced heterogeneity to trivial (0%) and changed the *SMD* value to 0.18 (data not shown). The funnel plots of effect sizes revealed asymmetries, which were further confirmed by Egger’s test of intercept. The test indicated considerable asymmetry for measures of static (*β₀* = 4.075, 95% CI = 1.91–6.24, *t* = 3.691, *p* < .001), dynamic (*β₀* = 5.546, 95% CI = 3.01–8.08, *t* = 4.294, *p* < .001), and reac*t*ive (*β₀* = 7.975, 95% CI = 4.56–11.39, *t *= 4.582, *p* < .01) balance, suggesting *t*hat the distribution of effect sizes may be biased due to selective publication of studies. However, for measures of proactive balance, no considerable asymmetry was detected.

### Contribution of arm movement on balance performance: Effect of task difficulty

The comparison of balance performance between free and restricted arm movement condition depending on the level of task difficulty is shown in Table 4. Regardless of the balance domain, the weighted mean *SMD* values indicate better performance under the free arm movement condition. In addition, the values for tasks with a high difficulty level (e.g., standing with eyes closed) were higher than those with a low difficulty level (e.g., standing with eyes opened). Consequently, the positive influence of free arm movement on postural control appears to be stronger when the level of balance task difficulty increases. No *SMD* values could be calculated for proactive and reactive balance due to a lack of or too few studies that compared varying degrees of task difficulty.

**Table 4:** Differences between restricted and free arm movement condition depending on the level of task difficulty by balance domain.

Balance domain	Low difficulty level	High difficulty level
Static balance	0.20 [0.04, 0.36]; *I*² = 65%	0.89 [0.56, 1.22]; *I*² = 74%
Dynamic balance	0.76 [-0.02, 1.55]; *I*² = 91%	1.04 [0.14, 1.94]; *I*² = 93%
Proactive balance	*N/A*	*N/A*
Reactive balance	*N/A*	*N/A*

Note. Data are presented as standardized mean difference (*SMD*) and the 95% confidence interval in parentheses., SMD values with a positive sign indicate an effect in favour of the free arm movement condition. *I*^2^ means heterogeneity between studies, *N/A* not applicable

## Discussion

To the best of our knowledge, the present systematic review and meta-analysis is the first to characterise, aggregate, and quantify performance differences between free and restricted arm movement conditions for different balance categories with varying levels of task difficulty in healthy individuals. The analysis of 25 studies including 725 participants (*n *= 331 females) revealed positive, moderate effects of free *versus* restricted arm movements, irrespective of balance domain (i.e., static, dynamic, proactive, or reactive balance). The results additionally indicate that for static and dynamic balance tasks, the positive influence of free arm movement was more pronounced in test conditions with a high (e.g., standing with eyes closed) compared to a low (e.g., standing with eyes opened) difficulty level. Below, we discuss the potential mechanisms and implications for testing and/or training programmes.

### Effects of free arm movement on balance performance

A consistent finding in the current systematic review with meta-analysis was that of a moderate improvement in balance performance during free *versus* restricted arm movements, supporting the first part of our hypothesis. This effect was relatively consistent across static (*SMD* = 0.51) [10,11,21–26], dynamic (*SMD* = 0.66) [6,7,9,22–24,27–33], proactive (*SMD* = 0.52) [6,8,10,22–24,34–36], and reactive (*SMD* = 0.50) [6,28,29,37–39] balance domains. The observation that different domains of balance performance were robustly affected by free arm movements highlights the broad functional relevance of arm movement strategies on postural control.

Although the mechanisms through which arm movements positively influence balance performance remain unclear, researchers have attributed the effect to mechanical factors associated with outstretching the arms. For example, greater dispersion of body mass increases the moment of inertia, which should theoretically increase stability of the postural control system [6]. Additionally, arm movements may be used to generate restoring torques to reduce angular momentum of the body [40] and act as counterweight to shift the centre of mass away from the direction of instability [41]. From a neural perspective, allowing free arm movements may utilise more practiced motor patterns and therefore result in better automatic control of balance. It also seems plausible that allowing the arms to move freely may enhance awareness of limb position and movement, contributing to enhanced proprioceptive feedback. Although the present study is not able to answer the question regarding which mechanisms govern the improved balance performance with free arm movements, it is important to acknowledge that individual balance components (i.e., static, dynamic, proactive, and reactive) represent relatively independent motor skills [42]. Thus, arm movements may affect balance components differently, possibly due to different postural mechanisms involved.

While the present findings emphasize the compensatory role of arm movements in mitigating postural destabilization, existing evidence suggests that arm movements also contribute to balance recovery during tripping events [43] and to dynamic balance regulation during challenging locomotion tasks [7]. Future experimental studies should explore these mechanisms in detail using methods such as motion capture, biomechanical modelling, and neurophysiological assessments. Such research could elucidate how arm movements (i.e., upper-body strategy) interact with lower-body strategies to maintain balance under varying conditions, providing a more comprehensive understanding of postural control mechanisms.

### Moderating effect of task difficulty level

A further notable finding in the present systematic review with meta-analysis was that the effect of free arm movements on balance performance is influenced by the difficulty level of the balance task that is performed. That is, the positive effect of free arm movement on balance performance was more pronounced for tasks with a high-difficulty (*SMD* = 0.89 to 1.04) compared to low-difficulty (*SMD* = 0.20 to 0.76) level, confirming the second part of our hypothesis. It is important to highlight that these observations only relate to static and dynamic balance tasks, as too few studies have compared varying degrees of task difficulty during the assessment of proactive and reactive balance. This represents a significant research gap that warrants further investigation. Future research should prioritize these balance domains, particularly across varying levels of task difficulty, to provide a broader understanding of the compensatory role of arm movements.

Although it is difficult to generalise potential mechanisms across static and dynamic tasks (owing to their unique qualities [42]), there is clear evidence that the arms hierarchically compliment the lower body during both static [10,21] and dynamic [7,23] balance scenarios. In challenging balance situations, the centre of mass is more prone to shift closer to the limits of the base of support. Consequently, especially during difficult tasks, arm movements appear to play a critical role in making rapid adjustments to help restore balance. Thus, we propose that the observed improvements in balance performance when arm movements are permitted – particularly under challenging conditions – serve as indirect evidence that the arms play a “compensatory” role in mitigating postural destabilisation.

### Implications and recommendations

The present findings highlight the strong influence that arm restriction or movement can exert on several domains of balance performance in healthy individuals. As such, the observations presented here have important practical implications for both scientific assessments, training interventions, and daily life applications.

From a testing perspective, restricting arm movements is a relatively simple task manipulation that can be implemented in a flexible and implicit way. If the goal is to quantify maximal balance performance, then arm movements should be permitted, particularly when performing balance tasks with a high level of difficulty. Allowing arm movements is functionally more relevant, as it reflects how individuals naturally counter destabilization in real-world scenarios. However, it is important to acknowledge that the variability and dynamic nature of arm usage may complicate the interpretability of results. Conversely, if the goal is to provide a more challenging balance task and/or maximize standardization, restricting arm movements is recommended. This can be achieved by employing standardized positions, such as placing the hands on the hips, crossing the arms over the chest, or clasping the hands in front of the body at the waist. These restrictions isolate the contributions of the lower limb mechanisms, such as the ankle, knee, and hip, providing a clearer picture of their role in postural control. Additionally, combining both approaches in a dual-protocol assessment framework could enhance balance testing further. By measuring performance differences between free and restricted arm movement conditions, quantified as “Arm Restriction Cost” (ARC), it is possible to evaluate the extent to which individuals rely on arm movements to maintain stability.

From a training perspective, the observed improvements in balance performance with free arm movements suggest that permitting arm movements may serve as a valuable starting point in a continuum of balance training. Free arm movements allow individuals, particularly those with limited balance control, to establish baseline stability by utilizing natural compensatory mechanisms. As balance proficiency increases, progressively introducing arm restrictions can elevate task difficulty by reducing the moment of inertia and encouraging greater reliance on proximal-to-distal coordination strategies involving the ankle, knee, and hip. This structured progression not only enhances postural control but also strengthens anticipatory and reactive balance mechanisms. Such an approach offers a clear and systematic framework for practitioners to design and implement balance training programs that are adaptable to the needs and capabilities of different populations.

Beyond testing and training, these findings also have meaningful implications for daily life. Promoting free arm movements during balance-demanding activities, such as walking on uneven surfaces, climbing stairs, or recovering from sudden destabilizations, supports functional balance and reduces the risk of falls. This recommendation is particularly relevant for populations such as older adults and children, whose postural control systems are often less developed. Moreover, integrating constraint-based strategies into everyday tasks such as carrying objects or performing activities with restricted arm movements can enhance lower-body stability by promoting reliance on core and lower limb mechanisms. Such strategies may also improve adaptability and strengthen postural responses over time.

Regardless of whether the focus is on testing, training, or daily life, it is essential to clearly define and describe arm movement instructions during balance assessments. This practice helps to avoid misinterpretation of performance outcomes and facilitates experimental replication. Future research should aim to explore the long-term effects of dual-protocol assessments and progressive training strategies, particularly in populations at higher risk of falls, to expand our understanding of how arm movements contribute to balance performance and functional outcomes.

### Limitations

This systematic review with meta-analysis has a few limitations. Firstly, the applied methodology varied across the included studies in terms of participant characteristics (e.g., age group, gender distribution), assessment methods (e.g., test type, outcome measures), and test modalities (e.g., type of sensory manipulation). These differences resulted in substantial to considerable heterogeneity between studies, which may partially hinder the interpretability of findings. The application of age-specific subgroup analyses and the Leave-One-Out method reduced heterogeneity in some areas. Therefore, future studies should provide more precise information on participant age. Additionally, direct comparisons between multiple age groups within a single study are necessary to allow age-related conclusions about the role of arm movements on balance performance. Secondly, while the sample size was sufficient to conduct a basic meta-analysis addressing our primary research questions, it was not large enough to allow for comparisons between potential moderators such as age and task difficulty in the context of proactive and reactive balance. Thirdly, our meta-analysis does not provide insights into how reduced balance performance under restricted arm movement conditions translates into functional outcomes, such as an increased risk of falls or a higher incidence of injuries.

Fourthly, the observed publication bias suggests that studies with small sample sizes, but large effects were published more frequently. This may limit the generalizability of our findings and highlights the need for a more balanced consideration of the available evidence in future research.

## Conclusions

The present systematic review and meta-analysis characterised, aggregated, and quantified differences in balance performance between free and restricted arm movement conditions in healthy individuals. The analysis revealed positive, moderate effects of free arm movements on balance performance. The results additionally indicate that for static and dynamic tasks, the positive influence of free arm movement was more pronounced in high-difficulty compared to low-difficulty tasks. These findings underscore the highly adaptive nature of balance control, where the use of arm movements is modulated based on the demands of the task at hand. The synthesis of this evidence serves as a benchmark to enhance the reporting practices and standardisation of arm movement assessments in postural control literature. These findings will also be influential in guiding future efforts to develop training interventions to improve the use of the arms in activities of daily living.

## Supporting information

S1 Table Included and excluded studies. (PDF)

S2 Table PRISMA Checklist: transparent reporting of systematic reviews and meta-analyses [Moher et al., 2009]. (DOC)

S3 Table Table of all data extracted from the primary research. (XLSX)

S4 Table Quality assessment of included studies using the appraisal tool for cross-sectional studies [Downes et al., 2016]. (XLSX)
