# Supplementary material for: Help! – you need your hands: Contribution of arm movements on balance performance in healthy individuals: A systematic review with meta-analysis
Source: PLoS One. 2025 May 8;20(5):e0323309. doi: 10.1371/journal.pone.0323309 (PMC12061164; doi:10.1371/journal.pone.0323309)
Supplement: S1 Table — (PDF) [file pone.0323309.s001.pdf]

	Title	Authors	Spalte1	Journal/Book	Publication Year	DOI	Inclusion / Exclusion	Reason for Exclusion
1	Upper-Extremity Functional Performance Tests: Reference Values for Overhead Athletes	Borms D, Cools A.	PubMed	Int J Sports Med	2018	10.1055/a-0573-1388	excluded	No comparison of arm movement in balance or postural control was conducted
2	Upper Quarter Y Balance Test: reliability and performance comparison between genders in active adults	Gorman PP, Butler RJ, Plisky PJ, Kiesel KB.	PubMed	J Strength Cond Res	2012	10.1519/JSC.0b013e3182472fdb	excluded	No comparison of arm movement in balance or postural control was conducted
3	Association of Dance-Based Mind-Motor Activities With Falls and Physical Function Among Healthy Older Adults: A Systematic Review and Meta-analysis	Mattle M, Chocano-Bedoya PO, Fischbacher M, Meyer U, Abderhalden LA, Lang W, Mansky R, Kressig RW, Steurer J, Orav EJ, Bischoff-Ferrari HA.	PubMed	JAMA Netw Open	2020	10.1001/jamanetworkopen.2020.17688	excluded	No comparison of arm movement in balance or postural control was conducted
4	Correlation between Core Stability and Upper-Extremity Performance in Male Collegiate Athletes	Nuhmani S.	PubMed	Medicina (Kaunas)	2022	10.3390/medicina58080982	excluded	No comparison of arm movement in balance or postural control was conducted
5	Efficacy of core stability training on upper extremity performance in collegiate athletes	Jha P, Nuhmani S, Kapoor G, Al Muslem WH, Joseph R, Kachanathu SJ, Alsaadi SM.	PubMed	J Musculoskelet Neuronal Interact	2022		excluded	No comparison of arm movement in balance or postural control was conducted
6	Management of Upper Extremity Injury in Divers	Haase SC.	PubMed	Hand Clin	2017	10.1016/j.hcl.2016.08.017	excluded	No comparison of arm movement in balance or postural control was conducted

7	Does postural stability affect grasping?	Voudouris D, Radhakrishnan S, Hatzitaki V, Brenner E.	PubMed	Gait Posture	2013	10.1016/j.gaitpost.2013.01.016	excluded	No comparison of arm movement in balance or postural control was conducted
8	Estimating the sources of motor errors for adaptation and generalization	Berniker M, Kording K.	PubMed	Nat Neurosci	2008	10.1038/nn.2229	excluded	No comparison of arm movement in balance or postural control was conducted
9	Postural control during gait termination and prehension	Jeong H, Cabiles N, van Emmerik REA.	PubMed	Gait Posture	2024	10.1016/j.gaitpost.2024.04.020	excluded	No comparison of arm movement in balance or postural control was conducted
10	Modified Functional Reach Test: Upper-Body Kinematics and Muscular Activity in Chronic Stroke Survivors	Marchesi G, Ballardini G, Barone L, Giannoni P, Lentino C, De Luca A, Casadio M.	PubMed	Sensors (Basel)	2021	10.3390/s22010230	excluded	No comparison of arm movement in balance or postural control was conducted
11	Beyond therapists: Technology-aided physical MS rehabilitation delivery	Feys P, Straudi S.	PubMed	Mult Scler	2019	10.1177/1352458519848968	excluded	No comparison of arm movement in balance or postural control was conducted
12	Age-related changes in mobility assessments correlate with repetitive goal-directed arm-movement performance	Walz ID, Waibel S, Kuhner A, Gollhofer A, Maurer C.	PubMed	BMC Geriatr	2023	10.1186/s12877-023-04150-3	excluded	No comparison of arm movement in balance or postural control was conducted
13	Effect of Standing on a Standardized Measure of Upper Extremity Function	Stewart JC, Saba A, Baird JF, Kolar MB, O'Donnell M, Schaefer SY.	PubMed	OTJR (Thorofare N J)	2021	10.1177/1539449220937058	excluded	No comparison of arm movement in balance or postural control was conducted
14	Test-retest reliability of upper-limb proprioception and balance tests in older nursing home residents	Galhardas L, Raimundo A, Marmeleira J.	PubMed	Arch Gerontol Geriatr	2020	10.1016/j.archger.2020.104079	excluded	No comparison of arm movement in balance or postural control was conducted

15	Postural balance and oculomotor control are influenced by neck kinaesthetic functions in elite ice hockey players	Majcen Rosker Z, Kristjansson E, Vodicar M, Rosker J.	PubMed	Gait Posture	2021	10.1016/j.gaitpost.2021.01.024	excluded	No comparison of arm movement in balance or postural control was conducted
16	Baseline Assessments of Strength and Balance Performance and Bilateral Asymmetries in Collegiate Athletes	Dai B, Layer J, Vertz C, Hinshaw T, Cook R, Li Y, Sha Z.	PubMed	J Strength Cond Res	2019	10.1519/JSC.0000000000002687	excluded	No comparison of arm movement in balance or postural control was conducted
17	Effect of active arm swing to local dynamic stability during walking	Wu Y, Li Y, Liu AM, Xiao F, Wang YZ, Hu F, Chen JL, Dai KR, Gu DY.	PubMed	Hum Mov Sci	2016	10.1016/j.humov.2015.10.005	excluded	No comparison of arm movement (free vs restricted)
18	Co-ordination of the upper and lower limbs for vestibular control of balance	Smith CP, Allsop JE, Mistry M, Reynolds RF.	PubMed	J Physiol	2017	10.1113/JP274272	excluded	No comparison of arm movement (free vs restricted)
19	**Role of arm motion in feet-in-place balance recovery**	**Cheng KB, Wang KM, Kuo SY.**	**PubMed**	**J Biomech**	**2015**	**10.1016/j.jbiomech.2015.07.008**	**included**	
20	Upper-Extremity physical performance tests in older adults: Reference values, reliability and measurement error	Novais MEO, Oliveira AS, Moreira RP, Barbosa GMP, Lemos TV, Matheus JPC, de Souza Júnior JR.	PubMed	J Bodyw Mov Ther	2024	10.1016/j.jbmt.2024.10.007	excluded	No comparison of arm movement in balance or postural control was conducted
21	Upper body and ankle strategies compensate for reduced lateral stability at very slow walking speeds	Best AN, Wu AR.	PubMed	Proc Biol Sci	2020	10.1098/rspb.2020.1685	excluded	No comparison of arm movement in balance or postural control was conducted

22	Postural control and physiological responses to a simulated match in U-20 judo competitors	Santos L, Fernández-Río J, Iglesias-Soler E, Blanco-Traba M, Jakobsen MD, González-Díez V, Franchini E, Gutiérrez C, Dopico-Calvo X, Carballeira-Fernández E, Amonette W, Suman O.	PubMed	Sports Biomech	2020	10.1080/14763141.2018.1461237	excluded	No comparison of arm movement in balance or postural control was conducted
23	Contribution of arm movements to balance recovery after tripping in older adults	Bruijn SM, Sloot LH, Kingma I, Pijnappels M.	PubMed	J Biomech	2022	10.1016/j.jbiomech.2022.110981	excluded	No comparison of arm movement (free vs restricted)
24	Upper body balance control strategy during continuous 3D postural perturbation in young adults	Amori V, Petrarca M, Patané F, Castelli E, Cappa P.	PubMed	Gait Posture	2015	10.1016/j.gaitpost.2014.08.003	excluded	No comparison of arm movement in balance or postural control was conducted
25	Variations in cricket players' upper body dynamic balance across different levels of competition	Singla D, Hussain ME.	PubMed	Int J Adolesc Med Health	2020	10.1515/ijamh-2019-0015	excluded	No comparison of arm movement in balance or postural control was conducted
26	Visual field motion during a body pull affects compensatory standing and stepping responses	St George RJ, Di Giulio I, Day BL.	PubMed	J Physiol	2020	10.1113/JP275436	excluded	No comparison of arm movement in balance or postural control was conducted
27	Ability and stability of running and walking in children with cerebral palsy	Iosa M, Morelli D, Marro T, Paolucci S, Fusco A.	PubMed	Neuropediatrics	2013	10.1055/s-0033-1336016	excluded	No comparison of arm movement in balance or postural control was conducted
28	**Effect of arm swing on single-step balance recovery**	**Cheng KB, Huang YC, Kuo SY.**	**PubMed**	**Hum Mov Sci**	**2014**	**10.1016/j.humov.2014.08.011**	**included**	

29	Targeted box and blocks test: Normative data and comparison to standard tests	Kontson K, Marcus I, Myklebust B, Civillico E.	PubMed	PLoS One	2017	10.1371/journal.pone.0177965	excluded	No comparison of arm movement in balance or postural control was conducted
30	Reduced gait and postural stability under challenging conditions in fallers with upper limb fracture	Langeard A, Pothier K, Chastan N, Marcelli C, Chavoix C, Bessot N.	PubMed	Aging Clin Exp Res	2019	10.1007/s40520-018-0992-z	excluded	No comparison of arm movement in balance or postural control was conducted
31	Are the shoulder joint function, stability, and mobility tests predictive of handstand execution?	Malíř R, Chrudimský J, Provazník A, Třebický V.	PubMed	PLoS One	2024	10.1371/journal.pone.0302922	excluded	No comparison of arm movement in balance or postural control was conducted
32	**Arm movement improves performance in clinical balance and mobility tests**	**Milosevic M, McConville KM, Masani K.**	**PubMed**	**Gait Posture**	**2011**	**10.1016/j.gaitpost.2010.12.005**	**included**	
33	Effects of inter-limb asymmetries on physical and sports performance: a systematic review	Bishop C, Turner A, Read P.	PubMed	J Sports Sci	2018	10.1080/02640414.2017.1361894	excluded	No comparison of arm movement in balance or postural control was conducted
34	A balance and proprioception intervention programme to enhance combat performance in military personnel	Funk S, Jacob T, Ben-Dov D, Yanovich E, Tirosh O, Steinberg N.	PubMed	J R Army Med Corps	2018	10.1136/jramc-2017-000809	excluded	No comparison of arm movement in balance or postural control was conducted
35	Adaptation in motor strategies for postural control associated with sensory reweighting	Dewan BM, James CR, Kumar NA, Burgess N, Zupancic S, Sawyer SF.	PubMed	Hum Mov Sci	2023	10.1016/j.humov.2023.103098	excluded	No comparison of arm movement in balance or postural control was conducted

36	Relationships between balance and physical fitness variables in firefighter recruits	Marciniak RA, Ebersole KT, Cornell DJ.	PubMed	Work	2021	10.3233/WOR-203401	excluded	No comparison of arm movement in balance or postural control was conducted
37	Quantification of reactive arm responses to a slip perturbation	Lee-Confer JS, Bradley NS, Powers CM.	PubMed	J Biomech	2022	10.1016/j.jbiomech.2022.110967	excluded	no comparsion between free and restricted arm movement
38	International vs. national female tennis players: a comparison of upper and lower extremity functional asymmetries	Chapelle L, Bishop C, Clarys P, D'Hondt E.	PubMed	J Sports Med Phys Fitness	2022	10.23736/S0022-4707.21.12482-X	excluded	No comparison of arm movement in balance or postural control was conducted
39	Effects of upper body strength, hand placement and foot placement on ladder fall severity	Pliner EM, Seo NJ, Ramakrishnan V, Beschorner KE.	PubMed	Gait Posture	2019	10.1016/j.gaitpost.2018.10.035	excluded	No comparison of arm movement in balance or postural control was conducted
40	Do postural constraints affect eye, head, and arm coordination?	Stamenkovic A, Stapley PJ, Robins R, Hollands MA.	PubMed	J Neurophysiol	2018	10.1152/jn.00200.2018	excluded	No comparison of arm movement in balance or postural control was conducted
41	Role of active joint position sense on the upper extremity functional performance tests in college volleyball players	Mendez-Rebolledo G, Ager AL, Ledezma D, Montanez J, Guerrero-Henriquez J, Cruz-Montecinos C.	PubMed	PeerJ	2022	10.7717/peerj.13564	excluded	No comparison of arm movement in balance or postural control was conducted
42	**Active arm swing and asymmetric walking leads to increased variability in trunk kinematics in young adults**	**Siragy T, Mezher C, Hill A, Nantel J.**	**PubMed**	**J Biomech**	**2020**	**10.1016/j.jbiomech.2019.109529**	**included**	

43	Role of angular position of the seat in control of posture in response to external perturbation	Ademiluyi A, Liang H, Aruin AS.	PubMed	Exp Brain Res	2022	10.1007/s00221-021-06270-x	excluded	No comparison of arm movement in balance or postural control was conducted
44	Postural adaptation during arm raising in children with and without unilateral cerebral palsy	Ledebt A, Savelsbergh GJ.	PubMed	Res Dev Disabil	2014	10.1016/j.ridd.2014.02.007	excluded	No comparison of arm movement in balance or postural control was conducted
45	The Relationship Between Functional Movement, Dynamic Stability, and Athletic Performance Assessments in Baseball and Softball Athletes	Stapleton DT, Boergers RJ, Rodriguez J, Green G, Johnson K, Williams P, Leelum N, Jackson L, Vallorosi J.	PubMed	J Strength Cond Res	2021	10.1519/JSC.0000000000003781	excluded	No comparison of arm movement in balance or postural control was conducted
46	**Exploring how arm movement moderates the effect of task difficulty on balance performance in young and older adults**	**Johnson E, Ellmers TJ, Muehlbauer T, Lord SR, Hill MW.**	**PubMed**	**Hum Mov Sci**	**2023**	**10.1016/j.humov.2023.103093**	**included**	
47	Seated postural organization during bilateral upper limb symmetric and asymmetric pushing tasks in individuals after stroke compared to healthy controls	Akremi H, Higgins J, Guediri A, Aissaoui R, Nadeau S.	PubMed	Gait Posture	2023	10.1016/j.gaitpost.2023.06.011	excluded	No comparison of arm movement in balance or postural control was conducted
48	Anthropometric and demographic properties affect balance in healthy adults: an observational study	Kurt M, Tatlici E, Tarsuslu Simsek T.	PubMed	J Sports Med Phys Fitness	2021	10.23736/S0022-4707.20.11422-1	excluded	No comparison of arm movement in balance or postural control was conducted

49	Relationship between upper limb physical performance tests and muscle strength of scapular, shoulder and spine stabilizers: A cross-sectional study	Guirelli AR, Dos Santos JM, Cabral EMG, Pinto JPC, De Lima GA, Felicio LR.	PubMed	J Bodyw Mov Ther	2021	10.1016/j.jbmt.2021.05.014	excluded	No comparison of arm movement in balance or postural control was conducted
50	Usage Position and Virtual Keyboard Design Affect Upper-Body Kinematics, Discomfort, and Usability during Prolonged Tablet Typing	Lin MI, Hong RH, Chang JH, Ke XM.	PubMed	PLoS One	2015	10.1371/journal.pone.0143585	excluded	No comparison of arm movement in balance or postural control was conducted
51	Rapid and flexible whole body postural responses are evoked from perturbations to the upper limb during goal-directed reaching	Lowrey CR, Nashed JY, Scott SH.	PubMed	J Neurophysiol	2017	10.1152/jn.01004.2015	excluded	grip context
52	The effect of increased cognitive processing on reactive balance control following perturbations to the upper limb	Trotman M, Kennefick M, Coughlin S, van Donkelaar P, Dalton BH.	PubMed	Exp Brain Res	2022	10.1007/s00221-022-06326-6	excluded	grip context
53	Automation of the Timed Up and Go Test Using a Doppler Radar System for Gait and Balance Analysis in Elderly People	Soubra R, Mourad-Chehade F, Chkeir A.	PubMed	J Healthc Eng	2023	10.1155/2023/2016262	excluded	No comparison of arm movement in balance or postural control was conducted
54	Effects of gait pattern and arm swing on intergirdle coordination	Dedieu P, Zanone PG.	PubMed	Hum Mov Sci	2012	10.1016/j.humov.2011.07.009	excluded	No comparison of arm movement (free vs restricted)

55	Unconstrained slip mechanics and stepping reactions depend on slip onset timing	Rasmussen CM, Hunt NH.	PubMed	J Biomech	2021	10.1016/j.jbiomech.2021.110572	excluded	No comparison of arm movement in balance or postural control was conducted
56	Investigation of Motor Activity, Movement Kinematics and Forward-Backwards Gait in Children with Cerebral Palsy	Özden F, Uysal İ, Tümtürk İ, Özkeskin M.	PubMed	Percept Mot Skills	2023	10.1177/00315125231191152	excluded	No comparison of arm movement in balance or postural control was conducted
57	Control and calibration of multi-segment reaching movements	Lackner JR, DiZio P.	PubMed	Adv Exp Med Biol	2009	10.1007/978-0-387-77064-2_37	excluded	No comparison of arm movement in balance or postural control was conducted
58	Sagittal-plane balance perturbations during very slow walking: Strategies for recovering linear and angular momentum	van Mierlo M, Vlutters M, van Asseldonk EHF, van der Kooij H.	PubMed	J Biomech	2023	10.1016/j.jbiomech.2023.111580	excluded	No comparison of arm movement in balance or postural control was conducted
59	Accelerometry: a technique for quantifying movement patterns during walking	Kavanagh JJ, Menz HB.	PubMed	Gait Posture	2008	10.1016/j.gaitpost.2007.10.010	excluded	No comparison of arm movement in balance or postural control was conducted
60	Skill level constrains the coordination of posture and upper-limb movement in a pistol-aiming task	Ko JH, Han DW, Newell KM.	PubMed	Hum Mov Sci	2017	10.1016/j.humov.2017.08.017	excluded	No comparison of arm movement in balance or postural control was conducted
61	Influence of base of support on early postural adjustments and fencing lunge performance	Akbaş A, Marszałek W, Bacik B, Juras G.	PubMed	Sports Biomech	2024	10.1080/14763141.2021.1987510	excluded	No comparison of arm movement in balance or postural control was conducted

62	Emergence of postural patterns as a function of vision and translation frequency	Buchanan JJ, Horak FB.	PubMed	J Neurophysiol	1999	10.1152/jn.1999.81.5.2325	excluded	No comparison of arm movement in balance or postural control was conducted
63	Postural control during upper body locomotor-like movements: similar synergies based on dissimilar muscle modes	Danna-Dos-Santos A, Shapkova EY, Shapkova AL, Degani AM, Latash ML.	PubMed	Exp Brain Res	2009	10.1007/s00221-008-1659-3	excluded	No comparison of arm movement in balance or postural control was conducted
64	Age-related differences in postural adjustments during limb movement and motor imagery in young and older adults	Wider C, Mitra S, Andrews M, Boulton H.	PubMed	Exp Brain Res	2020	10.1007/s00221-020-05751-9	excluded	No comparison of arm movement in balance or postural control was conducted
65	An evaluation of upper-body muscle activation during coupled and uncoupled instability resistance training	Campbell BM, Kutz MR, Morgan AL, Fullenkamp AM, Ballenger R.	PubMed	J Strength Cond Res	2014	10.1519/JSC.0000000000000349	excluded	No comparison of arm movement in balance or postural control was conducted
66	Estimating Gait Stability: Asymmetrical Loading Effects Measured Using Margin of Stability and Local Dynamic Stability	Worden TA, Beaudette SM, Brown SH, Vallis LA.	PubMed	J Mot Behav	2016	10.1080/00222895.2015.1134433	excluded	No comparison of arm movement in balance or postural control was conducted
67	Immediate Effects of Limb Rotational Kinesio Tape Application on Upper Quarter Y-Balance Test Scores	Dittmer A, Tomchuk D, Fontenot DR.	PubMed	J Sport Rehabil	2020	10.1123/jsr.2019-0145	excluded	No comparison of arm movement in balance or postural control was conducted

68	Human push capability	Barnett RL, Liber T.	PubMed	Ergonomics	2006	10.1080/00140130500122516	excluded	No comparison of arm movement in balance or postural control was conducted
69	Age-related differences in dynamic balance control during stair descent and effect of varying step geometry	Novak AC, Komisar V, Maki BE, Fernie GR.	PubMed	Appl Ergon	2016	10.1016/j.apergo.2015.07.027	excluded	No comparison of arm movement in balance or postural control was conducted
70	Anticipatory postural control associated with bilateral arm flexion and event-related potential in a Kanji Stroop-like task	Shen X, Fujiwara K, Tomita H.	PubMed	Clin Neurophysiol	2009	10.1016/j.clinph.2009.02.163	excluded	No comparison of arm movement in balance or postural control was conducted
71	Arm crank ergometer "spin" training improves seated balance and aerobic capacity in people with spinal cord injury	Williams AMM, Chisholm AE, Lynn A, Malik RN, Eginyan G, Lam T.	PubMed	Scand J Med Sci Sports	2020	10.1111/sms.13580	excluded	No comparison of arm movement in balance or postural control was conducted
72	Protective balance and startle responses to sudden freefall in standing humans	Sanders OP, Savin DN, Creath RA, Rogers MW.	PubMed	Neurosci Lett	2015	10.1016/j.neulet.2014.11.034	excluded	No comparison of arm movement in balance or postural control was conducted
73	Practice-related improvements in postural control during rapid arm movement in older adults: a preliminary study	Kubicki A, Petrement G, Bonnetblanc F, Ballay Y, Mourey F.	PubMed	J Gerontol A Biol Sci Med Sci	2012	10.1093/gerona/glr148	excluded	No comparison of arm movement in balance or postural control was conducted
74	Constraining the arms during a slip perturbation results in a higher fall frequency in young adults	Lee-Confer JS, Kulig K, Powers CM.	PubMed	Hum Mov Sci	2022	10.1016/j.humov.2022.103016	excluded	between subject design

75	Effect of perturbation timing on recovering whole-body angular momentum during very slow walking	van Mierlo M, Abma M, Vlutters M, van Asseldonk EHF, van der Kooij H.	PubMed	Hum Mov Sci	2023	10.1016/j.humov.2023.103138	excluded	No comparison of arm movement in balance or postural control was conducted
76	Side differences in upper quarter mobility/stability are not related to serve velocity in tennis players with different levels of training experience	Lambrich J, Panzer S, Muehlbauer T.	PubMed	BMC Res Notes	2024	10.1186/s13104-024-06944-z	excluded	No comparison of arm movement in balance or postural control was conducted
77	Physical and Physiological Determinants of Rock Climbing	MacKenzie R, Monaghan L, Masson RA, Werner AK, Caprez TS, Johnston L, Kemi OJ.	PubMed	Int J Sports Physiol Perform	2020	10.1123/ijspp.2018-0901	excluded	No comparison of arm movement in balance or postural control was conducted
78	Control of head stability during gait initiation in young and older women	Laudani L, Casabona A, Perciavalle V, Macaluso A.	PubMed	J Electromyogr Kinesiol	2006	10.1016/j.jelekin.2006.08.001	excluded	No comparison of arm movement in balance or postural control was conducted
79	The impact of a total knee arthroplasty on jaw movements, upper body posture, plantar pressure distribution, and postural control	Heil L, Maltry L, Lehmann S, Heil D, Lehmann C, Kopp S, Wanke EM, Bendels MHK, Groneberg DA, Ohlendorf D.	PubMed	Cranio	2021	10.1080/08869634.2019.1574999	excluded	No comparison of arm movement in balance or postural control was conducted
80	Validity, Reliability and Reproducibility of OctoBalance Test as Tool to Measure the Upper Limb Compared to Modified-Upper Quarter Y-Balance Test	Velarde-Sotres Á, Bores-Cerezal A, Mecías-Calvo M, Carvajal-Altamiranda S, Calleja-González J.	PubMed	Int J Environ Res Public Health	2021	10.3390/ijerph18105057	excluded	No comparison of arm movement in balance or postural control was conducted

81	Repeated Exposure to Forward Support-Surface Perturbation During Overground Walking Alters Upper-Body Kinematics and Step Parameters	Inkol KA, Huntley AH, Vallis LA.	PubMed	J Mot Behav	2019	10.1080/00222895.2018.1474336	excluded	No comparison of arm movement in balance or postural control was conducted
82	Impact of Extremity Manipulation on Postural Sway Characteristics: A Preliminary, Randomized Crossover Study	Malaya CA, Haworth J, Pohlman KA, Powell C, Smith DL.	PubMed	J Manipulative Physiol Ther	2020	10.1016/j.jmpt.2019.02.014	excluded	No comparison of arm movement in balance or postural control was conducted
83	Internal models in sensorimotor integration: perspectives from adaptive control theory	Tin C, Poon CS.	PubMed	J Neural Eng	2005	10.1088/1741-2560/2/3/S01	excluded	No comparison of arm movement in balance or postural control was conducted
84	Nordic Walking Can Be Incorporated in the Exercise Prescription to Increase Aerobic Capacity, Strength, and Quality of Life for Elderly: A Systematic Review and Meta-Analysis	Bullo V, Gobbo S, Vendramin B, Duregon F, Cugusi L, Di Blasio A, Bocalini DS, Zaccaria M, Bergamin M, Ermolao A.	PubMed	Rejuvenation Res	2018	10.1089/rej.2017.1921	excluded	No comparison of arm movement in balance or postural control was conducted
85	Trunk posture affects upper extremity function of adults	Gillen G, Boiangiu C, Neuman M, Reinstein R, Schaap Y.	PubMed	Percept Mot Skills	2007	10.2466/pms.104.2.371-380	excluded	No comparison of arm movement in balance or postural control was conducted
86	Influences of arm proprioception and degrees of freedom on postural control with light touch feedback	Rabin E, DiZio P, Ventura J, Lackner JR.	PubMed	J Neurophysiol	2008	10.1152/jn.00504.2007	excluded	No comparison of arm movement in balance or postural control was conducted

87	Is angular momentum in the horizontal plane during gait a controlled variable?	Thielemans V, Meyns P, Bruijn SM.	PubMed	Hum Mov Sci	2014	10.1016/j.humov.2014.03.003	excluded	No comparison of arm movement in balance or postural control was conducted
88	Coordination of hip and spine to maintain equilibrium in unstable sitting revealed by spectral analysis	Alshehri MA, van den Hoorn W, Klyne DM, Hodges PW.	PubMed	J Neurophysiol	2021	10.1152/jn.00555.2020	excluded	No comparison of arm movement in balance or postural control was conducted
89	The contribution of counter-rotation movements during fall recovery: A validation study	Rapp van Roden EA, Petersen DA, Pigman J, Conner BC, Tyler Richardson R, Crenshaw JR.	PubMed	J Biomech	2018	10.1016/j.jbiomech.2018.07.025	excluded	no balance parameter
90	Are simultaneous postural adjustments (SPA) programmed as a function of pointing velocity?	Fourcade P, Le Bozec S, Bouisset S.	PubMed	Exp Brain Res	2016	10.1007/s00221-016-4683-8	excluded	No comparison of arm movement in balance or postural control was conducted
91	Body mass index and physical function in older women	Apovian CM, Frey CM, Wood GC, Rogers JZ, Still CD, Jensen GL.	PubMed	Obes Res	2002	10.1038/oby.2002.101	excluded	No comparison of arm movement in balance or postural control was conducted
92	Controller synthesis and clinical exploration of wearable gyroscopic actuators to support human balance	Lemus D, Berry A, Jabeen S, Jayaraman C, Hohl K, van der Helm FCT, Jayaraman A, Vallery H.	PubMed	Sci Rep	2020	10.1038/s41598-020-66760-w	excluded	No comparison of arm movement in balance or postural control was conducted
93	Influence of age on functional capacity and work ability in Chilean workers: a cross-sectional study	Marzuca-Nassr GN, Soto-Rodríguez FJ, Bascour-Sandoval C, Cofré-Obando V, Hermosilla C, Sepúlveda P, Muñoz-Poblete C.	PubMed	Int Arch Occup Environ Health	2021	10.1007/s00420-021-01674-2	excluded	No comparison of arm movement in balance or postural control was conducted

94	High-Speed Cycling Intervention Improves Rate-Dependent Mobility in Older Adults	Bellumori M, Uygur M, Knight CA.	PubMed	Med Sci Sports Exerc	2017	10.1249/MSS.0000000000001069	excluded	No comparison of arm movement in balance or postural control was conducted
95	Anticipatory postural adjustments modify the movement-related potentials of upper extremity voluntary movement	Yoshida S, Nakazawa K, Shimizu E, Shimoyama I.	PubMed	Gait Posture	2008	10.1016/j.gaitpost.2007.02.006	excluded	No comparison of arm movement in balance or postural control was conducted
96	Effect of crutch and walking-boot use on whole-body angular momentum during gait	Wiederien RC, Gari WJ, Wilken JM.	PubMed	Assist Technol	2024	10.1080/10400435.2023.2229879	excluded	No comparison of arm movement in balance or postural control was conducted
97	Reliability and relationships among handgrip strength, leg extensor strength and power, and balance in older men	Jenkins ND, Buckner SL, Bergstrom HC, Cochrane KC, Goldsmith JA, Housh TJ, Johnson GO, Schmidt RJ, Cramer JT.	PubMed	Exp Gerontol	2014	10.1016/j.exger.2014.07.007	excluded	No comparison of arm movement in balance or postural control was conducted
98	The degree of postural automaticity influences the prime movement and the anticipatory postural adjustments during standing in healthy young individuals	Sakamoto S, Iguchi M.	PubMed	Hum Mov Sci	2018	10.1016/j.humov.2018.06.002	excluded	No comparison of arm movement in balance or postural control was conducted
99	Constancy of Preparatory Postural Adjustments for Reaching to Virtual Targets across Different Postural Configurations	Stamenkovic A, Hollands MA, Stapley PJ.	PubMed	Neuroscience	2021	10.1016/j.neuroscience.2020.11.009	excluded	No comparison of arm movement in balance or postural control was conducted

100	Magnitude, symmetry and attenuation of upper body accelerations during walking in women: The role of age, fall history and walking surface	Soleimanifar M, Mazaheri M, van Schooten KS, Asgari M, Mosallanezhad Z, Salavati M, Sedaghat-Nejad E, Parnianpour M.	PubMed	Maturitas	2020	10.1016/j.maturitas.2020.05.016	excluded	No comparison of arm movement in balance or postural control was conducted
101	The impact of a total hip replacement on jaw position, upper body posture and body sway	Ohlendorf D, Lehmann C, Heil D, Hörzer S, Kopp S.	PubMed	Cranio	2015	10.1179/2151090314Y.0000000012	excluded	No comparison of arm movement in balance or postural control was conducted
102	Effects of aging and arm swing on the metabolic cost of stability in human walking	Ortega JD, Fehlman LA, Farley CT.	PubMed	J Biomech	2008	10.1016/j.jbiomech.2008.06.039	excluded	no balance parameter
103	Asymmetrical load-carrying while stepping down a curb in young adults	Silva JJ, Rinaldi NM, Moraes R.	PubMed	Gait Posture	2019	10.1016/j.gaitpost.2019.07.306	excluded	No comparison of arm movement in balance or postural control was conducted
104	Fall arrest strategy training improves upper body response time compared to standard fall prevention exercise in older women: A randomized trial	Arnold CM, Lanovaz J, Farthing JP, Legg H, Weimer M, Kim S.	PubMed	Clin Rehabil	2022	10.1177/02692155221087963	excluded	No comparison of arm movement in balance or postural control was conducted
105	Effects of balance constraints during a double-step reaching task	Moya-Jofré C, Mariman JJ, Bruna-Melo T, Carrasco-Plaza J, Torres-Elgueta J, Aleitte-Leyton F, Muñoz-Puelman C, Horak FB, Mancini M, Burgos PI.	PubMed	Gait Posture	2024	10.1016/j.gaitpost.2024.05.018	excluded	No comparison of arm movement in balance or postural control was conducted

106	Potential contributing factors to upper limb associated reactions in people with acquired brain injury: an exploratory study	Kahn MB, Clark RA, Mentiplay BF, Bower KJ, Olver J, Williams G.	PubMed	Disabil Rehabil	2022	10.1080/09638288.2021.1887945	excluded	No comparison of arm movement in balance or postural control was conducted
107	The coupling between upper and lower extremity synergies during whole body reaching	Kaminski TR.	PubMed	Gait Posture	2007	10.1016/j.gaitpost.2006.09.006	excluded	No comparison of arm movement in balance or postural control was conducted
108	The associations of physical parameters with the Closed Kinetic Chain Upper Extremity Stability Test, the Upper Quarter Y Balance Test, and the Upper Limb Rotation Test in professional overhead athletes	Kara F, Ergin Gedik G, Şahinoğlu E.	PubMed	Phys Ther Sport	2024	10.1016/j.ptsp.2024.03.001	excluded	No comparison of arm movement in balance or postural control was conducted
109	Allocation of attention and dual-task effects on upper and lower limb task performance in healthy young adults	McIsaac TL, Benjapalakorn B.	PubMed	Exp Brain Res	2015	10.1007/s00221-015-4333-6	excluded	No comparison of arm movement in balance or postural control was conducted
110	Descriptive Profile of Lumbopelvic Control in Collegiate Baseball Pitchers	Laudner KG, Wong RH, Latal JR, Meister K.	PubMed	J Strength Cond Res	2018	10.1519/JSC.0000000000001946	excluded	No comparison of arm movement in balance or postural control was conducted
111	An integrated EMG/biomechanical model of upper body balance and posture during human gait	Winter DA, MacKinnon CD, Ruder GK, Wieman C.	PubMed	Prog Brain Res	1993	10.1016/s0079-6123(08)62295-5	excluded	No comparison of arm movement in balance or postural control was conducted

112	Anticipatory postural adjustments as a function of response complexity in simple reaction time tasks	Kennefick M, Wright AD, Smirl JD, van Donkelaar P.	PubMed	Neurosci Lett	2018	10.1016/j.neulet.2018.06.058	excluded	No comparison of arm movement in balance or postural control was conducted
113	Acquisition of upper body stability during walking in toddlers	Ledebt A, Bril B.	PubMed	Dev Psychobiol	2000	10.1002/(sici)1098-2302(200005)36:4<311::aid-dev6>3.0.co;2-v	excluded	No comparison of arm movement in balance or postural control was conducted
114	Effects of a body manipulation of Japanese martial arts on interpersonal correlation of postural sway	Watanabe Y, Sakaguchi Y.	PubMed	PLoS One	2022	10.1371/journal.pone.0274294	excluded	No comparison of arm movement in balance or postural control was conducted
115	Test-Retest Reliability of Movement Displacement during a 20-s Stepping-in-Place Test in Community-Dwelling Older Adults with and without Supportive Care	Fujita E, Takeshima N, Sato H, Kohama T, Kusunoki M, Oba Y, Brechue WF.	PubMed	Gerontology	2024	10.1159/000539748	excluded	No comparison of arm movement in balance or postural control was conducted
116	Influence of stance width on frontal plane postural dynamics and coordination in human balance control	Goodworth AD, Peterka RJ.	PubMed	J Neurophysiol	2010	10.1152/jn.00916.2009	excluded	No comparison of arm movement in balance or postural control was conducted
117	The effect of a neuromuscular-cognitive training program on postural stability, hop performance, and agility in Division-I Women's Tennis athletes: A pilot study	Porter KH, Ochoa L, Torp DM, Hoch MC.	PubMed	Musculoskelet Sci Pract	2024	10.1016/j.msksp.2024.103214	excluded	No comparison of arm movement in balance or postural control was conducted

118	Biomechanics in posture space: Properties and relevance of principal accelerations for characterizing movement control	Longo A, Haid T, Meulenbroek R, Federolf P.	PubMed	J Biomech	2019	10.1016/j.jbiomech.2018.11.031	excluded	No comparison of arm movement in balance or postural control was conducted
119	Constructing a Talent Identification Index System and Evaluation Model for Cross-Country Skiers	Huang X, Wang G, Chen C, Liu J, Kristiansen B, Hohmann A, Zhao K.	PubMed	J Sports Sci	2021	10.1080/02640414.2020.1823084	excluded	No comparison of arm movement in balance or postural control was conducted
120	Effects of Internal and External Attentional Focus on Postural Response to a Sliding Stance Surface	Kunimura H, Matsuoka M, Hamada N, Hiraoka K.	PubMed	Percept Mot Skills	2019	10.1177/0031512519838688	excluded	No comparison of arm movement in balance or postural control was conducted
121	Frontal plane standing balance with an ambulation aid: Upper limb biomechanics	Tung JY, Gage WH, Zabjek KF, Maki BE, McIlroy WE.	PubMed	J Biomech	2011	10.1016/j.jbiomech.2011.03.015	excluded	No comparison of arm movement in balance or postural control was conducted
122	Upper and lower extremity proprioceptive inputs modulate EMG activity of the trapezius	Tataroglu C, Kuçuk FK, Ozkul A.	PubMed	J Electromyogr Kinesiol	2011	10.1016/j.jelekin.2010.09.011	excluded	No comparison of arm movement in balance or postural control was conducted
123	Measuring methods for functional reach test: comparison of 1-arm reach and 2-arm reach	Kage H, Okuda M, Nakamura I, Kunitsugu I, Sugiyama S, Hobara T.	PubMed	Arch Phys Med Rehabil	2009	10.1016/j.apmr.2009.07.021	excluded	No comparison of arm movement in balance or postural control was conducted
124	Block-Periodized Training Improves Physiological and Tactically Relevant Performance in Naval Special Warfare Operators	Abt JP, Oliver JM, Nagai T, Sell TC, Lovalekar MT, Beals K, Wood DE, Lephart SM.	PubMed	J Strength Cond Res	2016	10.1519/JSC.0000000000001082	excluded	No comparison of arm movement in balance or postural control was conducted

125	Quantitative assessment of trunk movements in functional reaching in children and adolescents with dyskinetic cerebral palsy	Van Wonterghem E, Vanmechelen I, Haberfehlner H, Decrock B, Monbaliu E.	PubMed	Clin Biomech (Bristol)	2023	10.1016/j.clinbiomech.2023.105876	excluded	No comparison of arm movement in balance or postural control was conducted
126	Rapid adaptation to Coriolis force perturbations of voluntary body sway	Bakshi A, DiZio P, Lackner JR.	PubMed	J Neurophysiol	2019	10.1152/jn.00606.2018	excluded	No comparison of arm movement in balance or postural control was conducted
127	Control of the upper body accelerations in young and elderly women during level walking	Mazzà C, Iosa M, Pecoraro F, Cappozzo A.	PubMed	J Neuroeng Rehabil	2008	10.1186/1743-0003-5-30	excluded	No comparison of arm movement (free vs restricted)
128	Female Age-Related Differences in Biomechanics and Muscle Activity During Descents on the Outstretched Arms	Lattimer LJ, Lanovaz JL, Farthing JP, Madill S, Kim S, Robinovitch S, Arnold C.	PubMed	J Aging Phys Act	2017	10.1123/japa.2016-0102	excluded	No comparison of arm movement (free vs restricted)
129	The nature of arm movement in children with cerebral palsy when using computer-generated exercise games	Weightman A, Preston N, Levesley M, Bhakta B, Holt R, Mon-Williams M.	PubMed	Disabil Rehabil Assist Technol	2014	10.3109/17483107.2013.782576	excluded	No comparison of arm movement in balance or postural control was conducted
130	Role of muscle coactivation in adaptation of standing posture during arm reaching	Pienciak-Siewert A, Horan DP, Ahmed AA.	PubMed	J Neurophysiol	2020	10.1152/jn.00939.2017	excluded	No comparison of arm movement in balance or postural control was conducted
131	Functional fitness norms and trends of community-dwelling older adults in urban China	Zhao Y, Wang Z, Chung PK, Wang S.	PubMed	Sci Rep	2021	10.1038/s41598-021-97320-5	excluded	No comparison of arm movement in balance or postural control was conducted

132	The effect of upper limb casting on gait pattern	Dreyfuss D, Elbaz A, Mor A, Segal G, Calif E.	PubMed	Int J Rehabil Res	2016	10.1097/MRR.0000000000000155	excluded	gait and casting
133	A controller for walking derived from how humans recover from perturbations	Joshi V, Srinivasan M.	PubMed	J R Soc Interface	2019	10.1098/rsif.2019.0027	excluded	No free arm movement
134	A New Approach to Improve Cognition, Muscle Strength, and Postural Balance in Community-Dwelling Elderly with a 3-D Virtual Reality Kayak Program	Park J, Yim J.	PubMed	Tohoku J Exp Med	2016	10.1620/tjem.238.1	excluded	No comparison of arm movement in balance or postural control was conducted
135	Effect of vibration-induced postural illusion on anticipatory postural adjustment of voluntary arm movement in standing humans	Kasai T, Yahagi S, Shimura K.	PubMed	Gait Posture	2002	10.1016/s0966-6362(01)00177-1	excluded	No comparison of arm movement in balance or postural control was conducted
136	Influence of a passive lower-limb exoskeleton during simulated industrial work tasks on physical load, upper body posture, postural control and discomfort	Luger T, Seibt R, Cobb TJ, Rieger MA, Steinhilber B.	PubMed	Appl Ergon	2019	10.1016/j.apergo.2019.05.018	excluded	No comparison of arm movement in balance or postural control was conducted
137	A research on the postural stability of a person wearing the lower limb exoskeletal robot by the HAT model	Chang M, Kim Y, Lee Y, Jeon D.	PubMed	IEEE Int Conf Rehabil Robot	2017	10.1109/ICORR.2017.8009275	excluded	No comparison of arm movement in balance or postural control was conducted

138	The Effects of Virtual Reality Nonphysical Mental Training on Coordination and Skill Transfer in Healthy Adults	Köyağasıoğlu O, Özgürbüz C.	PubMed	J Sport Rehabil	2022	10.1123/jsr.2021-0198	excluded	No comparison of arm movement in balance or postural control was conducted
139	Synthesis of natural arm swing motion in human bipedal walking	Park J.	PubMed	J Biomech	2008	10.1016/j.jbiomech.2008.02.031	excluded	No comparison of arm movement in balance or postural control was conducted
140	Effects of age and gender on maximum voluntary range of motion of the upper body joints	Doriot N, Wang X.	PubMed	Ergonomics	2006	10.1080/00140130500489873	excluded	No comparison of arm movement in balance or postural control was conducted
141	Effects of visual focus and gait speed on walking balance in the frontal plane	Goodworth A, Perrone K, Pillsbury M, Yargeau M.	PubMed	Hum Mov Sci	2015	10.1016/j.humov.2015.04.004	excluded	No comparison of arm movement in balance or postural control was conducted
142	Coordination between postural and movement controls: effect of changes in body mass distribution on postural and focal component characteristics	Robert G, Blouin J, Ruget H, Mouchnino L.	PubMed	Exp Brain Res	2007	10.1007/s00221-007-0916-1	excluded	No comparison of arm movement in balance or postural control was conducted
143	The effect of Otago exercises on fear of falling, balance, empowerment and functional mobility in the older people: Randomized controlled trial	Genç FZ, Bilgili N.	PubMed	Int J Nurs Pract	2023	10.1111/ijn.13194	excluded	No comparison of arm movement in balance or postural control was conducted
144	Reactive responses of the arms increase the Margins of Stability and decrease center of mass dynamics during a slip perturbation	Lee-Confer JS, Finley JM, Kulig K, Powers CM.	PubMed	J Biomech	2023	10.1016/j.jbiomech.2023.111737	excluded	between subject design

145	Visual-vestibular interactions in postural control during the execution of a dynamic task	Bent LR, McFadyen BJ, Inglis JT.	PubMed	Exp Brain Res	2002	10.1007/s00221-002-1204-8	excluded	No comparison of arm movement in balance or postural control was conducted
146	Reproducibility and validity of the Nintendo Wii Balance Board for measuring shoulder sensorimotor control in prone lying	Eshoj H, Juul-Kristensen B, Jørgensen RGB, Søgaard K.	PubMed	Gait Posture	2017	10.1016/j.gaitpost.2016.12.003	excluded	No comparison of arm movement in balance or postural control was conducted
147	Testing the acceptability of motorcycle-AEB system: Use of unanticipated interventions as a reliable surrogate of genuinely unexpected events	Huertas-Leyva P, Savino G, Baldanzini N.	PubMed	Traffic Inj Prev	2023	10.1080/15389588.2023.2165881	excluded	No comparison of arm movement in balance or postural control was conducted
148	Effects of variation in external pulling force magnitude, elevation, and orientation on trunk muscle forces, spinal loads and stability	El Ouaaid Z, Shirazi-Adl A, Plamondon A.	PubMed	J Biomech	2016	10.1016/j.jbiomech.2015.09.036	excluded	No comparison of arm movement in balance or postural control was conducted
149	Identifying Individuals Who Currently Report Feelings of Anxiety Using Walking Gait and Quiet Balance: An Exploratory Study Using Machine Learning	Stark M, Huang H, Yu LF, Martin R, McCarthy R, Locke E, Yager C, Torad AA, Kadry AM, Elwan MA, Smith ML, Bradley D, Boolani A.	PubMed	Sensors (Basel)	2022	10.3390/s22093163	excluded	No comparison of arm movement in balance or postural control was conducted

150	The effects of walking speed and prosthetic ankle adapters on upper extremity dynamics and stability-related parameters in bilateral transtibial amputee gait	Major MJ, Stine RL, Gard SA.	PubMed	Gait Posture	2013	10.1016/j.gaitpost.2013.04.012	excluded	No comparison of arm movement in balance or postural control was conducted
151	The energetic cost of maintaining lateral balance during human running	Arellano CJ, Kram R.	PubMed	J Appl Physiol (1985)	2012	10.1152/japplphysiol.00554.2011	excluded	No comparison of arm movement in balance or postural control was conducted
152	Modeling the postural disturbances caused by upper extremity movements	Triolo RJ, Werner KN, Kirsch RF.	PubMed	IEEE Trans Neural Syst Rehabil Eng	2001	10.1109/7333.928573	excluded	No comparison of arm movement in balance or postural control was conducted
153	Effects of upper limb positions and weight support roles on quasi-static seated postural stability in individuals with spinal cord injury	Grangeon M, Gagnon D, Gauthier C, Jacquemin G, Masani K, Popovic MR.	PubMed	Gait Posture	2012	10.1016/j.gaitpost.2012.05.021	excluded	not representative of a healthy population
154	Haptic touch reduces sway by increasing axial tone	Franzén E, Gurfinkel VS, Wright WG, Cordo PJ, Horak FB.	PubMed	Neuroscience	2011	10.1016/j.neuroscience.2010.11.017	excluded	No comparison of arm movement in balance or postural control was conducted
155	Comparison of Walking, Muscle Strength, Balance, and Fear of Falling Between Repeated Fall Group, One-time Fall Group, and Nonfall Group of the Elderly Receiving Home Care Service	Jeon M, Gu MO, Yim J.	PubMed	Asian Nurs Res (Korean Soc Nurs Sci)	2017	10.1016/j.anr.2017.11.003	excluded	No comparison of arm movement in balance or postural control was conducted

156	A pointing task to improve reaching performance in older adults	Engler SA, Lilly KA, Perkins J, Ustinova KI.	PubMed	Am J Phys Med Rehabil	2011	10.1097/PHM.0b013e31820b1367	excluded	No comparison of arm movement in balance or postural control was conducted
157	Hand dominance and multi-finger synergies	Zhang W, Sainburg RL, Zatsiorsky VM, Latash ML.	PubMed	Neurosci Lett	2006	10.1016/j.neulet.2006.09.048	excluded	No comparison of arm movement in balance or postural control was conducted
158	A complete, non-lumped, and verifiable set of upper body segment parameters for three-dimensional dynamic modeling	Vette AH, Yoshida T, Thrasher TA, Masani K, Popovic MR.	PubMed	Med Eng Phys	2011	10.1016/j.medengphy.2010.09.008	excluded	No comparison of arm movement in balance or postural control was conducted
159	Contribution of sensorimotor integration to spinal stabilization in humans	Goodworth AD, Peterka RJ.	PubMed	J Neurophysiol	2009	10.1152/jn.00118.2009	excluded	No comparison of arm movement in balance or postural control was conducted
160	Directional control of planar human arm movement	Gottlieb GL, Song Q, Almeida GL, Hong DA, Corcos D.	PubMed	J Neurophysiol	1997	10.1152/jn.1997.78.6.2985	excluded	No balance or postural control
161	Incomplete posture adjustment during rapid arm movement	Yamasaki H, Fujisawa H, Hoshi F, Nagasaki H.	PubMed	Percept Mot Skills	2009	10.2466/PMS.108.3.915-932	excluded	No balance or postural control
162	Stabilizing function of the diaphragm: dynamic MRI and synchronized spirometric assessment	Kolar P, Sulc J, Kyncl M, Sanda J, Neuwirth J, Bokarius AV, Kriz J, Kobesova A.	PubMed	J Appl Physiol (1985)	2010	10.1152/japplphysiol.01216.2009	excluded	No comparison of arm movement in balance or postural control was conducted
163	Stance width changes how sensory feedback is used for multisegmental balance control	Goodworth AD, Mellodge P, Peterka RJ.	PubMed	J Neurophysiol	2014	10.1152/jn.00490.2013	excluded	No comparison of arm movement in balance or postural control was conducted

164	Effects of regular heel-raise training aimed at the soleus muscle on dynamic balance associated with arm movement in elderly women	Fujiwara K, Toyama H, Asai H, Yaguchi C, Irei M, Naka M, Kaida C.	PubMed	J Strength Cond Res	2011	10.1519/JSC.0b013e3181fb4947	excluded	No comparison of arm movement (free vs restricted)
165	Center of mass acceleration feedback control of standing balance by functional neuromuscular stimulation against external postural perturbations	Nataraj R, Audu ML, Triolo RJ.	PubMed	IEEE Trans Biomed Eng	2013	10.1109/TBME.2012.2218601	excluded	No comparison of arm movement in balance or postural control was conducted
166	Effects of anticipation certainty on preparatory brain activity and anticipatory postural adjustments associated with voluntary unilateral arm movement while standing	Tomita H, Fujiwara K, Mori E, Sakurai A.	PubMed	Hum Mov Sci	2012	10.1016/j.humov.2011.07.013	excluded	No comparison of arm movement in balance or postural control was conducted
167	Physiological consequences of using an upper limb exoskeleton during manual handling tasks	Theurel J, Desbrosses K, Roux T, Savescu A.	PubMed	Appl Ergon	2018	10.1016/j.apergo.2017.10.008	excluded	No comparison of arm movement in balance or postural control was conducted
168	A mathematical model of the stability control of human thorax and pelvis movements during walking	Wu Q, Swain R.	PubMed	Comput Methods Biomech Biomed Engin	2002	10.1080/1025584021000001632	excluded	No comparison of arm movement in balance or postural control was conducted

169	Associations between gait coordination, variability and motor cortex inhibition in young and older adults	Swanson CW, Fling BW.	PubMed	Exp Gerontol	2018	10.1016/j.exger.2018.10.002	excluded	No comparison of arm movement in balance or postural control was conducted
170	Reliability of the Star Excursion Balance Test and Two New Similar Protocols to Measure Trunk Postural Control	López-Plaza D, Juan-Recio C, Barbado D, Ruiz-Pérez I, Vera-Garcia FJ.	PubMed	PM R	2018	10.1016/j.pmrj.2018.05.012	excluded	No comparison of arm movement in balance or postural control was conducted
171	The Benefits of Custom Exergames for Fitness, Balance, and Health-Related Quality of Life: A Randomized Controlled Trial with Community-Dwelling Older Adults	Gonçalves A, Muñoz J, Cameirão MS, Gouveia ÉR, Sousa H, Bermúdez I Badia S.	PubMed	Games Health J	2021	10.1089/g4h.2020.0092	excluded	No comparison of arm movement in balance or postural control was conducted
172	Age and task differences in functional fitness in older women: comparisons with Senior Fitness Test normative and criterion-referenced data	Adamo DE, Talley SA, Goldberg A.	PubMed	J Aging Phys Act	2015	10.1123/japa.2012-0317	excluded	No comparison of arm movement in balance or postural control was conducted
173	Efficacy of instability resistance training	Cowley PM, Swensen T, Sforzo GA.	PubMed	Int J Sports Med	2007	10.1055/s-2007-964893	excluded	No comparison of arm movement in balance or postural control was conducted
174	Effects of allocation of visuo-spatial attention to visual stimuli triggering unilateral arm abduction on anticipatory postural control	Tomita H, Fujiwara K.	PubMed	Clin Neurophysiol	2008	10.1016/j.clinph.2008.05.001	excluded	No comparison of arm movement in balance or postural control was conducted

175	Foot placement alters the mechanisms of postural control while standing and reaching	Gillette JC, Abbas JJ.	PubMed	IEEE Trans Neural Syst Rehabil Eng	2003	10.1109/TNSRE.2003.819790	excluded	No comparison of arm movement in balance or postural control was conducted
176	Identification of Risk Factors for Injury in Women's Collegiate Gymnastics With the Gymnastics Functional Measurement Tool	Ling D, Sleeper M, Casey E.	PubMed	PM R	2020	10.1002/pmrj.12184	excluded	No comparison of arm movement in balance or postural control was conducted
177	The effects of strength exercise on hippocampus volume and functional fitness of older women	Kim YS, Shin SK, Hong SB, Kim HJ.	PubMed	Exp Gerontol	2017	10.1016/j.exger.2017.07.007	excluded	No comparison of arm movement in balance or postural control was conducted
178	Physical fitness differences between prepubescent boys and girls	Marta CC, Marinho DA, Barbosa TM, Izquierdo M, Marques MC.	PubMed	J Strength Cond Res	2012	10.1519/JSC.0b013e31825bb4aa	excluded	No comparison of arm movement in balance or postural control was conducted
179	Recovery from perturbations during paced walking	Oddsson LI, Wall C, McPartland MD, Krebs DE, Tucker CA.	PubMed	Gait Posture	2004	10.1016/s0966-6362(03)00008-0	excluded	No comparison of arm movement in balance or postural control was conducted
180	Upper Limb Strength and Muscle Volume in Healthy Middle-Aged Adults	Saul KR, Vidt ME, Gold GE, Murray WM.	PubMed	J Appl Biomech	2015	10.1123/jab.2014-0177	excluded	No comparison of arm movement in balance or postural control was conducted
181	Effect of lower limb muscle fatigue induced by high-level isometric contractions on postural maintenance and postural adjustments associated with bilateral forward-reach task	Yiou E, Heugas AM, Mezaour M, Le Bozec S.	PubMed	Gait Posture	2009	10.1016/j.gaitpost.2008.07.003	excluded	No comparison of arm movement in balance or postural control was conducted

182	Support stability influences postural responses to muscle vibration in humans	Ivanenko YP, Talis VL, Kazennikov OV.	PubMed	Eur J Neurosci	1999	10.1046/j.1460-9568.1999.00471.x	excluded	No comparison of arm movement in balance or postural control was conducted
183	Comparative electromyography analysis of the upper extremity between inexperienced and elite water polo players during an overhead shot	Yaghoubi M, Esfehani MM, Hosseini HA, Alikhajeh Y, Shultz SP.	PubMed	J Appl Biomech	2015	10.1123/jab.2014-0068	excluded	No comparison of arm movement in balance or postural control was conducted
184	Effects of a cognitive dual task on variability and local dynamic stability in sustained repetitive arm movements using principal component analysis: a pilot study	Longo A, Federolf P, Haid T, Meulenbroek R.	PubMed	Exp Brain Res	2018	10.1007/s00221-018-5241-3	excluded	No comparison of arm movement in balance or postural control was conducted
185	Postural responses in the upper limbs evoked by axial impulses: a role for reticulospinal projections	Teng B, Govender S, Colebatch JG.	PubMed	Exp Brain Res	2017	10.1007/s00221-017-4963-y	excluded	No comparison of arm movement in balance or postural control was conducted
186	Lumbar and cervical erector spinae fatigue elicit compensatory postural responses to assist in maintaining head stability during walking	Kavanagh JJ, Morrison S, Barrett RS.	PubMed	J Appl Physiol (1985)	2006	10.1152/japplphysiol.00165.2006	excluded	No comparison of arm movement in balance or postural control was conducted
187	The falling risk and physical fitness in older people	Toraman A, Yildirim NU.	PubMed	Arch Gerontol Geriatr	2010	10.1016/j.archger.2009.10.012	excluded	No comparison of arm movement in balance or postural control was conducted

188	Locomotor skills and balance strategies in children with internal rotations of the lower limbs	Mallau S, Mesure S, Viehweger E, Jacquemier M, Bollini G, Assaiante C.	PubMed	J Orthop Res	2008	10.1002/jor.20476	excluded	No comparison of arm movement in balance or postural control was conducted
189	Effects of visual deprivation on gait dynamic stability	Iosa M, Fusco A, Morone G, Paolucci S.	PubMed	ScientificWorldJournal	2012	10.1100/2012/974560	excluded	No comparison of arm movement in balance or postural control was conducted
190	Changes in the limits of stability induced by weight-shifting training in elderly women	Gouglidis V, Nikodelis T, Hatzitaki V, Amiridis IG.	PubMed	Exp Aging Res	2011	10.1080/0361073X.2010.507431	excluded	No comparison of arm movement in balance or postural control was conducted
191	Adaptation of postural orientation to changes in surface inclination	Kluzik J, Peterka RJ, Horak FB.	PubMed	Exp Brain Res	2007	10.1007/s00221-006-0715-0	excluded	No comparison of arm movement in balance or postural control was conducted
192	The influence of handrail predictability on compensatory arm reactions in response to a loss of balance	Weaver TB, Tokuno CD.	PubMed	Gait Posture	2013	10.1016/j.gaitpost.2012.12.003	excluded	No comparison of arm movement in balance or postural control was conducted
193	Strength, flexibility, and balance characteristics of highly proficient golfers	Sell TC, Tsai YS, Smoliga JM, Myers JB, Lephart SM.	PubMed	J Strength Cond Res	2007	10.1519/R-21826.1	excluded	No comparison of arm movement in balance or postural control was conducted
194	Effects of ventilation on body sway during human standing	Caron O, Fontanari P, Cremieux J, Joulia F.	PubMed	Neurosci Lett	2004	10.1016/j.neulet.2004.04.085	excluded	No comparison of arm movement in balance or postural control was conducted
195	Movement of the upper body and muscle activity patterns following a rapidly applied load: the influence of pre-load alterations	Andersen TB, Essendrop M, Schibye B.	PubMed	Eur J Appl Physiol	2004	10.1007/s00421-004-1040-6	excluded	No comparison of arm movement in balance or postural control was conducted

196	Prefrontal activation when suppressing an automatic balance recovery step	Abugu EU, Harper SA, Kim Y, Bolton DAE.	PubMed	Gait Posture	2024	10.1016/j.gaitpost.2023.10.016	excluded	No comparison of arm movement in balance or postural control was conducted
197	Effect of long-duration spaceflight on postural control during self-generated perturbations	Layne CS, Mulavara AP, McDonald PV, Pruett CJ, Kozlovskaya IB, Bloomberg JJ.	PubMed	J Appl Physiol (1985)	2001	10.1152/jappl.2001.90.3.997	excluded	No comparison of arm movement in balance or postural control was conducted
198	A comparison of minimum segment models for the estimation of centre of mass position and velocity for slip recovery during a bathtub transfer task	Collins M, Levine IC, Gosine PC, Montgomery RE, Nirmalanathan K, Novak AC.	PubMed	Gait Posture	2024	10.1016/j.gaitpost.2024.01.025	excluded	No comparison of arm movement in balance or postural control was conducted
199	Human body-segment tilts induced by galvanic stimulation: a vestibularly driven balance protection mechanism	Day BL, Séverac Cauquil A, Bartolomei L, Pastor MA, Lyon IN.	PubMed	J Physiol	1997	10.1113/jphysiol.1997.sp022051	excluded	No comparison of arm movement in balance or postural control was conducted
200	Continuous-scale physical functional performance in healthy older adults: a validation study	Cress ME, Buchner DM, Questad KA, Esselman PC, deLateur BJ, Schwartz RS.	PubMed	Arch Phys Med Rehabil	1996	10.1016/s0003-9993(96)90187-2	excluded	No comparison of arm movement in balance or postural control was conducted
201	Recruitment order of the abdominal muscles varies with postural task	Tokuno CD, Cresswell AG, Thorstensson A, Carpenter MG.	PubMed	Scand J Med Sci Sports	2013	10.1111/j.1600-0838.2011.01394.x	excluded	No comparison of arm movement in balance or postural control was conducted
202	Trunk stabilization during sagittal pelvic tilt: from trunk-on-pelvis to trunk-in-space due to vestibular and visual feedback	van Drunen P, van der Helm FC, van Dieën JH, Happee R.	PubMed	J Neurophysiol	2016	10.1152/jn.00867.2015	excluded	No comparison of arm movement in balance or postural control was conducted

203	Analysis of human postural responses to recoverable falls	Bortolami SB, DiZio P, Rabin E, Lackner JR.	PubMed	Exp Brain Res	2003	10.1007/s00221-003-1481-x	excluded	No comparison of arm movement in balance or postural control was conducted
204	Development of upper body coordination during sitting in typically developing infants	Kyvelidou A, Stuberg WA, Harbourne RT, Deffeyes JE, Blanke D, Stergiou N.	PubMed	Pediatr Res	2009	10.1203/PDR.0b013e31819d9051	excluded	No comparison of arm movement in balance or postural control was conducted
205	Influence of floor inclination on handle push and pull forces production of the upper limb	Abautret M, Monsabert BG, Maïs C, Rao G.	PubMed	Appl Ergon	2024	10.1016/j.apergo.2024.104322	excluded	No comparison of arm movement in balance or postural control was conducted
206	The effects of 12 weeks of step aerobics training on functional fitness of elderly women	Hallage T, Krause MP, Haile L, Miculis CP, Nagle EF, Reis RS, Da Silva SG.	PubMed	J Strength Cond Res	2010	10.1519/JSC.0b013e3181ddacc6	excluded	No comparison of arm movement in balance or postural control was conducted
207	Sensorimotor integration for multisegmental frontal plane balance control in humans	Goodworth AD, Peterka RJ.	PubMed	J Neurophysiol	2012	10.1152/jn.00670.2010	excluded	No comparison of arm movement in balance or postural control was conducted
208	Motor performance following a mild traumatic brain injury in children: an exploratory study	Gagnon I, Forget R, Sullivan SJ, Friedman D.	PubMed	Brain Inj	1998	10.1080/026990598122070	excluded	No comparison of arm movement in balance or postural control was conducted
209	Pilates for improvement of muscle endurance, flexibility, balance, and posture	Kloubec JA.	PubMed	J Strength Cond Res	2010	10.1519/JSC.0b013e3181c277a6	excluded	No comparison of arm movement in balance or postural control was conducted
210	How do children complete a seated combined cognitive and motor multi-tasking paradigm?	Hinton DC, Vallis LA.	PubMed	Hum Mov Sci	2015	10.1016/j.humov.2015.03.001	excluded	No comparison of arm movement in balance or postural control was conducted

211	Reliability and validity of physical fitness field tests for adults aged 55 to 70 years	Ritchie C, Trost SG, Brown W, Armit C.	PubMed	J Sci Med Sport	2005	10.1016/s1440-2440(05)80025-8	excluded	No comparison of arm movement in balance or postural control was conducted
212	Corset hypothesis rebutted--transversus abdominis does not co-contract in unison prior to rapid arm movements	Morris SL, Lay B, Allison GT.	PubMed	Clin Biomech (Bristol)	2012	10.1016/j.clinbiomech.2011.09.007	excluded	No comparison of arm movement in balance or postural control was conducted
213	Control of aperture closure initiation during trunk-assisted reach-to-grasp movements	Rand MK, Van Gemmert AW, Hossain AB, Shimansky YP, Stelmach GE.	PubMed	Exp Brain Res	2012	10.1007/s00221-012-3088-6	excluded	No comparison of arm movement in balance or postural control was conducted
214	Movement of finger joints induced by synergistic wrist motion	Su FC, Chou YL, Yang CS, Lin GT, An KN.	PubMed	Clin Biomech (Bristol)	2005	10.1016/j.clinbiomech.2005.01.002	excluded	No comparison of arm movement in balance or postural control was conducted
215	Muscle forces analysis in the shoulder mechanism during wheelchair propulsion	Lin HT, Su FC, Wu HW, An KN.	PubMed	Proc Inst Mech Eng H	2004	10.1243/0954411041561027	excluded	No comparison of arm movement in balance or postural control was conducted
216	Frontal plane dynamic margins of stability in individuals with and without transtibial amputation walking on a loose rock surface	Gates DH, Scott SJ, Wilken JM, Dingwell JB.	PubMed	Gait Posture	2013	10.1016/j.gaitpost.2013.01.024	excluded	No comparison of arm movement (free vs restricted)
217	Armed against falls: the contribution of arm movements to balance recovery after tripping	Pijnappels M, Kingma I, Wezenberg D, Reurink G, van Dieën JH.	PubMed	Exp Brain Res	2010	10.1007/s00221-009-2088-7	excluded	No comparison of arm movement in balance or postural control was conducted

218	Discrepancies in anthropometric parameters between different models affect intervertebral rotations when loading finite element models with muscle forces from inverse static analyses	Zhu R, Rohlmann A.	PubMed	Biomed Tech (Berl)	2014	10.1515/bmt-2013-0121	excluded	No comparison of arm movement in balance or postural control was conducted
219	How do children aged 6 to 11 stabilize themselves on an unstable sitting device? The progressive development of axial segment control	Pierret J, Beyaert C, Paysant J, Caudron S.	PubMed	Hum Mov Sci	2020	10.1016/j.humov.2020.102624	excluded	No comparison of arm movement in balance or postural control was conducted
220	The effects of split keyboard geometry on upper body postures	Rempel D, Nathan-Roberts D, Chen BY, Odell D.	PubMed	Ergonomics	2009	10.1080/00140130802481040	excluded	No comparison of arm movement in balance or postural control was conducted
221	A new biarticular actuator design facilitates control of leg function in BioBiped3	Sharbafi MA, Rode C, Kurowski S, Scholz D, Möckel R, Radkhah K, Zhao G, Rashty AM, Stryk Ov, Seyfarth A.	PubMed	Bioinspir Biomim	2016	10.1088/1748-3190/11/4/046003	excluded	No comparison of arm movement in balance or postural control was conducted
222	Directional postural responses induced by vibrotactile stimulations applied to the torso	Lee BC, Martin BJ, Sienko KH.	PubMed	Exp Brain Res	2012	10.1007/s00221-012-3233-2	excluded	No comparison of arm movement in balance or postural control was conducted
223	Effect of local leg cooling on upper limb trajectories and muscle function and whole body dynamic balance	Piedrahita H, Oksa J, Rintamäki H, Malm C.	PubMed	Eur J Appl Physiol	2009	10.1007/s00421-008-0920-6	excluded	No comparison of arm movement in balance or postural control was conducted

224	Role of the unperturbed limb and arms in the reactive recovery response to an unexpected slip during locomotion	Marigold DS, Bethune AJ, Patla AE.	PubMed	J Neurophysiol	2003	10.1152/jn.00683.2002	excluded	No comparison of arm movement (free vs restricted)
225	The effects of differential and variable training on the quality parameters of a handball throw	Wagner H, Müller E.	PubMed	Sports Biomech	2008	10.1080/14763140701689822	excluded	No comparison of arm movement in balance or postural control was conducted
226	Right-left sEMG burst synchronization of the lumbar erector spinae muscles of seated violin players	Khorrami Chokami A, Merletti R.	PubMed	Sci Rep	2024	10.1038/s41598-024-69531-z	excluded	No comparison of arm movement in balance or postural control was conducted
227	Functional fitness norms for community-dwelling older adults in Hong Kong	Chung PK, Zhao Y, Liu JD, Quach B.	PubMed	Arch Gerontol Geriatr	2016	10.1016/j.archger.2016.03.006	excluded	No comparison of arm movement in balance or postural control was conducted
228	The relationship between peak height velocity and physical performance in youth soccer players	Philippaerts RM, Vaeyens R, Janssens M, Van Renterghem B, Matthys D, Craen R, Bourgois J, Vrijens J, Beunen G, Malina RM.	PubMed	J Sports Sci	2006	10.1080/02640410500189371	excluded	No comparison of arm movement in balance or postural control was conducted
229	Health-related fitness test battery for adults: aspects of reliability	Suni JH, Oja P, Laukkanen RT, Miilunpalo SI, Pasanen ME, Vuori IM, Vartiainen TM, Bös K.	PubMed	Arch Phys Med Rehabil	1996	10.1016/s0003-9993(96)90092-1	excluded	No comparison of arm movement in balance or postural control was conducted

230	Effects of neck flexion on discriminative and cognitive processing in anticipatory postural control during bilateral arm movement	Fujiwara K, Yaguchi C, Kunita K, Mammadova A.	PubMed	Neurosci Lett	2012	10.1016/j.neulet.2012.04.073	excluded	No comparison of arm movement in balance or postural control was conducted
231	Effects of exercise on bone density and falls risk factors in post-menopausal women with osteopenia: a randomised controlled trial	Bolton KL, Egerton T, Wark J, Wee E, Matthews B, Kelly A, Craven R, Kantor S, Bennell KL.	PubMed	J Sci Med Sport	2012	10.1016/j.jsams.2011.08.007	excluded	No comparison of arm movement in balance or postural control was conducted
232	Assessing the influence of a passive, upper extremity exoskeletal vest for tasks requiring arm elevation: Part II - "Unexpected" effects on shoulder motion, balance, and spine loading	Kim S, Nussbaum MA, Mokhlespour Esfahani MI, Alemi MM, Jia B, Rashedi E.	PubMed	Appl Ergon	2018	10.1016/j.apergo.2018.02.024	excluded	No comparison of arm movement in balance or postural control was conducted
233	Late-cueing of gait tasks on an uneven brick surface impacts coordination and center of mass control in older adults	Dixon PC, Jacobs JV, Dennerlein JT, Schiffman JM.	PubMed	Gait Posture	2018	10.1016/j.gaitpost.2018.07.168	excluded	No comparison of arm movement in balance or postural control was conducted
234	A closed-loop self-righting controller for seated balance in the coronal and diagonal planes following spinal cord injury	Bheemreddy A, Lombardo LM, Miller ME, Foglyano KM, Nogan-Bailey S, Triolo RJ, Audu ML.	PubMed	Med Eng Phys	2020	10.1016/j.medengphy.2020.10.010	excluded	No comparison of arm movement in balance or postural control was conducted
235	The effects of dual-tasking on arm muscle responses in young and older adults	Laing JM, Tokuno CD.	PubMed	Hum Mov Sci	2016	10.1016/j.humov.2016.01.003	excluded	No comparison of arm movement in balance or postural control was conducted

236	Age-related differences in head and trunk coordination during walking	Kavanagh JJ, Barrett RS, Morrison S.	PubMed	Hum Mov Sci	2005	10.1016/j.humov.2005.07.003	excluded	No comparison of arm movement in balance or postural control was conducted
237	Multicomponent Physical Program: Effects on Physical Fitness of Older Women of Different Age Groups	Gonçalves AK, Silva PCD, Griebler EM, Silva WAD, Sant Helena DP, Possamai VD, Martins VF.	PubMed	Res Q Exerc Sport	2024	10.1080/02701367.2024.2306285	excluded	No comparison of arm movement in balance or postural control was conducted
238	Comparing joint kinematics and center of mass acceleration as feedback for control of standing balance by functional neuromuscular stimulation	Nataraj R, Audu ML, Triolo RJ.	PubMed	J Neuroeng Rehabil	2012	10.1186/1743-0003-9-25	excluded	No comparison of arm movement in balance or postural control was conducted
239	Effects of swimming on eye hand coordination and balance in the elderly	Hsu HC, Chou SW, Chen CP, Wong AM, Chen CK, Hong JP.	PubMed	J Nutr Health Aging	2010	10.1007/s12603-010-0134-6	excluded	No comparison of arm movement in balance or postural control was conducted
240	Transversus abdominis: changes in thickness during the unsupported upper limb exercise test in older adults	Fulton I, McEvoy M, Pieterse J, Williams M, Thoirs K, Petkov J.	PubMed	Physiother Theory Pract	2009	10.3109/09593980802665023	excluded	No comparison of arm movement in balance or postural control was conducted
241	Training unsupported sitting in people with chronic spinal cord injuries: a randomized controlled trial	Boswell-Ruys CL, Harvey LA, Barker JJ, Ben M, Middleton JW, Lord SR.	PubMed	Spinal Cord	2010	10.1038/sc.2009.88	excluded	No comparison of arm movement in balance or postural control was conducted
242	Contraction of the human diaphragm during rapid postural adjustments	Hodges PW, Butler JE, McKenzie DK, Gandevia SC.	PubMed	J Physiol	1997	10.1111/j.1469-7793.1997.539bb.x	excluded	No comparison of arm movement in balance or postural control was conducted

243	Effects of the lateral amplitude and regularity of upper body fluctuation on step time variability evaluated using return map analysis	Chidori K, Yamamoto Y.	PubMed	PLoS One	2017	10.1371/journal.pone.0180898	excluded	No comparison of arm movement in balance or postural control was conducted
244	Maturation of set-modulation of lower extremity EMG responses to postural perturbations	Müller K, Hömberg V, Coppenrath P, Lenard HG.	PubMed	Neuropediatrics	1992	10.1055/s-2008-1071318	excluded	No comparison of arm movement in balance or postural control was conducted
245	Posture-related modulations in motor cortical excitability of the proximal and distal arm muscles	Kantak SS, Wittenberg GF, Liao WW, Magder LS, Rogers MW, Waller SM.	PubMed	Neurosci Lett	2013	10.1016/j.neulet.2012.10.048	excluded	No comparison of arm movement in balance or postural control was conducted
246	Scaling of plantarflexor muscle activity and postural time-to-contact in response to upper-body perturbations in young and older adults	Hasson CJ, Caldwell GE, Van Emmerik RE.	PubMed	Exp Brain Res	2009	10.1007/s00221-009-1865-7	excluded	No comparison of arm movement in balance or postural control was conducted
247	Tablet Keyboard Configuration Affects Performance, Discomfort and Task Difficulty for Thumb Typing in a Two-Handed Grip	Trudeau MB, Catalano PJ, Jindrich DL, Dennerlein JT.	PubMed	PLoS One	2013	10.1371/journal.pone.0067525	excluded	No comparison of arm movement in balance or postural control was conducted

248	A multidimensional assessment of physical performance for older Japanese people with community-based long-term care needs	Kim M, Tanaka K.	PubMed	Aging Clin Exp Res	2014	10.1007/s40520-014-0230-2	excluded	No comparison of arm movement in balance or postural control was conducted
249	Vertical torque allows recording of anticipatory postural adjustments associated with slow, arm-raising movements	Bleuse S, Cassim F, Blatt JL, Defebvre L, Derambure P, Guieu JD.	PubMed	Clin Biomech (Bristol)	2005	10.1016/j.clinbiomech.2005.03.005	excluded	No comparison of arm movement in balance or postural control was conducted
250	Challenging stability limits in old and young individuals with a functional reaching task	Spreitzer L, Perkins J, Ustinova KI.	PubMed	Am J Phys Med Rehabil	2013	10.1097/PHM.0b013e318269d8f9	excluded	No comparison of arm movement (free vs restricted)
251	The effects of stance configuration and target distance on reaching. I. Movement preparation	Kaminski TR, Simpkins S.	PubMed	Exp Brain Res	2001	10.1007/s002210000604	excluded	No comparison of arm movement in balance or postural control was conducted
252	The regulation of vestibular afferent information during monocular vision while standing	Jessop D, McFadyen BJ.	PubMed	Neurosci Lett	2008	10.1016/j.neulet.2008.06.043	excluded	No comparison of arm movement in balance or postural control was conducted
253	Effectiveness of fingertip light contact in reducing postural sway in older people	Baccini M, Rinaldi LA, Federighi G, Vannucchi L, Paci M, Masotti G.	PubMed	Age Ageing	2007	10.1093/ageing/afl072	excluded	No comparison of arm movement in balance or postural control was conducted
254	Head stabilization in children of both genders during level walking	Mazzà C, Zok M, Cappozzo A.	PubMed	Gait Posture	2010	10.1016/j.gaitpost.2010.01.012	excluded	No comparison of arm movement in balance or postural control was conducted

255	Lower trunk motion and speed-dependence during walking	Kavanagh JJ.	PubMed	J Neuroeng Rehabil	2009	10.1186/1743-0003-6-9	excluded	No comparison of arm movement in balance or postural control was conducted
256	NACOB presentation CSB New Investigator Award. Balance recovery from medio-lateral perturbations of the upper body during standing. North American Congress on Biomechanics	Rietdyk S, Patla AE, Winter DA, Ishac MG, Little CE.	PubMed	J Biomech	1999	10.1016/s0021-9290(99)00116-5	excluded	No comparison of arm movement in balance or postural control was conducted
257	Influence of posture on blink reflex prepulse inhibition induced by somatosensory inputs from upper and lower limbs	Versace V, Campostrini S, Sebastianelli L, Saltuari L, Valls-Solé J, Kofler M.	PubMed	Gait Posture	2019	10.1016/j.gaitpost.2019.07.194	excluded	No comparison of arm movement in balance or postural control was conducted
258	Motor proficiency relationships among siblings	Wrotniak BH, Salvy SJ, Lazarus L, Epstein LH.	PubMed	Percept Mot Skills	2009	10.2466/PMS.108.1.112-120	excluded	No comparison of arm movement in balance or postural control was conducted
259	Balance recovery schemes following mediolateral gyroscopic moment perturbations during walking	Mohseni O, Mahmoudi A, Firouzi V, Seyfarth A, Vallery H, A Sharbafi M.	PubMed	PLoS One	2024	10.1371/journal.pone.0315414	excluded	No comparison of arm movement in balance or postural control was conducted
260	Effects of medio-lateral postural perturbation induced by voluntary arm raising on the biomechanical organization of rapid step initiation	Yiou E, Do MC.	PubMed	Motor Control	2011	10.1123/mcj.15.4.507	excluded	No comparison of arm movement in balance or postural control was conducted

261	Kinetic analysis of arm reaching movements during voluntary and passive rotation of the torso	Bortolami SB, Pigeon P, Dizio P, Lackner JR.	PubMed	Exp Brain Res	2008	10.1007/s00221-008-1321-0	excluded	No comparison of arm movement in balance or postural control was conducted
262	The limits of aerial twisting techniques in the aerials event of freestyle skiing	Yeadon MR.	PubMed	J Biomech	2013	10.1016/j.jbiomech.2012.11.029	excluded	No comparison of arm movement in balance or postural control was conducted
263	Psychometric properties of the Brazilian version of the Tilburg frailty indicator (TFI)	Santiago LM, Luz LL, Mattos IE, Gobbens RJ, van Assen MA.	PubMed	Arch Gerontol Geriatr	2013	10.1016/j.archger.2013.03.001	excluded	No comparison of arm movement in balance or postural control was conducted
264	Effects of a short-term whole body vibration intervention on physical fitness in elderly people	Gómez-Cabello A, González-Agüero A, Ara I, Casajús JA, Vicente-Rodríguez G.	PubMed	Maturitas	2013	10.1016/j.maturitas.2012.12.008	excluded	No comparison of arm movement in balance or postural control was conducted
265	Effect of isometric upper-extremity exercises on the activation of core stabilizing muscles	Tarnanen SP, Ylinen JJ, Siekkinen KM, Mälkiä EA, Kautiainen HJ, Häkkinen AH.	PubMed	Arch Phys Med Rehabil	2008	10.1016/j.apmr.2007.08.160	excluded	No comparison of arm movement in balance or postural control was conducted
266	Vestibular contributions across the execution of a voluntary forward step	Bent LR, Inglis JT, McFadyen BJ.	PubMed	Exp Brain Res	2002	10.1007/s00221-001-0967-7	excluded	No comparison of arm movement in balance or postural control was conducted
267	Effects of 16-weeks of Pilates on functional autonomy and life satisfaction among elderly women	Curi VS, Haas AN, Alves-Vilaça J, Fernandes HM.	PubMed	J Bodyw Mov Ther	2018	10.1016/j.jbmt.2017.06.014	excluded	No comparison of arm movement in balance or postural control was conducted
268	Theoretical contribution of the upper extremities to reducing trunk extension following a laboratory-induced slip	Troy KL, Donovan SJ, Grabiner MD.	PubMed	J Biomech	2009	10.1016/j.jbiomech.2009.03.004	excluded	No comparison of arm movement (free vs restricted)

269	Effect of structured physical activity on prevention of serious fall injuries in adults aged 70-89: randomized clinical trial (LIFE Study)	Gill TM, Pahor M, Guralnik JM, McDermott MM, King AC, Buford TW, Strotmeyer ES, Nelson ME, Sink KM, Demons JL, Kashaf SS, Walkup MP, Miller ME; LIFE Study Investigators.	PubMed	BMJ	2016	10.1136/bmj.i245	excluded	No comparison of arm movement in balance or postural control was conducted
270	The effect of a task-oriented walking intervention on improving balance self-efficacy poststroke: a randomized, controlled trial	Salbach NM, Mayo NE, Robichaud-Ekstrand S, Hanley JA, Richards CL, Wood-Dauphinee S.	PubMed	J Am Geriatr Soc	2005	10.1111/j.1532-5415.2005.53203.x	excluded	No comparison of arm movement in balance or postural control was conducted
271	Superposition and modulation of muscle synergies for reaching in response to a change in target location	d'Avella A, Portone A, Lacquaniti F.	PubMed	J Neurophysiol	2011	10.1152/jn.00675.2010	excluded	No comparison of arm movement in balance or postural control was conducted
272	The defence technique in Tai Chi Push Hands: a case study	Chen HC, Cheng KY, Liu YJ, Chiu HT, Cheng KY.	PubMed	J Sports Sci	2010	10.1080/02640414.2010.515238	excluded	No comparison of arm movement in balance or postural control was conducted
273	The effects of a customized over-the-counter mouth guard on neuromuscular force and power production in trained men and women	Dunn-Lewis C, Luk HY, Comstock BA, Szivak TK, Hooper DR, Kupchak BR, Watts AM, Putney BJ, Hydren JR, Volek JS, Denegar CR, Kraemer WJ.	PubMed	J Strength Cond Res	2012	10.1519/JSC.0b013e31824b4d5b	excluded	No comparison of arm movement in balance or postural control was conducted

274	Standardizing Methodology for Research with Uneven Terrains Focused on Dynamic Balance During Gait	Coleman TD, Lawrence HJ, Childers WL.	PubMed	J Appl Biomech	2016	10.1123/jab.2016-0014	excluded	No comparison of arm movement in balance or postural control was conducted
275	Functional Fitness and Quality of Life among Women over 60 Years of Age Depending on Their Level of Objectively Measured Physical Activity	Nawrocka A, Polechoński J, Garbaciak W, Mynarski W.	PubMed	Int J Environ Res Public Health	2019	10.3390/ijerph16060972	excluded	No comparison of arm movement in balance or postural control was conducted
276	Quantitative assessment of postural alignment in young adults based on photographs of anterior, posterior, and lateral views	Ferreira EA, Duarte M, Maldonado EP, Bersanetti AA, Marques AP.	PubMed	J Manipulative Physiol Ther	2011	10.1016/j.jmpt.2011.05.018	excluded	No comparison of arm movement in balance or postural control was conducted
277	Human adaptation to interaction forces in visuo-motor coordination	Huang FC, Gillespie RB, Kuo AD.	PubMed	IEEE Trans Neural Syst Rehabil Eng	2006	10.1109/TNSRE.2006.881533	excluded	No comparison of arm movement in balance or postural control was conducted
278	Older adults exhibit altered motor coordination during an upper limb object transport task requiring a lateral change in support	Huntley AH, Zettel JL, Vallis LA.	PubMed	Hum Mov Sci	2017	10.1016/j.humov.2017.01.014	excluded	No comparison of arm movement in balance or postural control was conducted
279	Directional sensitivity of "first trial" reactions in human balance control	Oude Nijhuis LB, Allum JH, Borm GF, Honegger F, Overeem S, Bloem BR.	PubMed	J Neurophysiol	2009	10.1152/jn.90945.2008	excluded	No comparison of arm movement in balance or postural control was conducted

280	Surface electromyography activity of trunk muscles during wheelchair propulsion	Yang YS, Koontz AM, Triolo RJ, Mercer JL, Boninger ML.	PubMed	Clin Biomech (Bristol)	2006	10.1016/j.clinbiomech.2006.07.006	excluded	No comparison of arm movement in balance or postural control was conducted
281	The effects of transcranial static magnetic fields stimulation over the supplementary motor area on anticipatory postural adjustments	Tsuru D, Watanabe T, Chen X, Kubo N, Sunagawa T, Mima T, Kirimoto H.	PubMed	Neurosci Lett	2020	10.1016/j.neulet.2020.134863	excluded	No comparison of arm movement in balance or postural control was conducted
282	Unknown loads affect force production capacity in early phases of bench press throws	Hernández Davó JL, Sabido Solana R, Sarabia Marínm JM, Sánchez Martos Á, Moya Ramón M.	PubMed	J Sports Med Phys Fitness	2015		excluded	No comparison of arm movement in balance or postural control was conducted
283	Unraveling interlimb interactions underlying bimanual coordination	Ridderikhoff A, Peper CL, Beek PJ.	PubMed	J Neurophysiol	2005	10.1152/jn.01077.2004	excluded	No comparison of arm movement in balance or postural control was conducted
284	Independent walking as a major skill for the development of anticipatory postural control: evidence from adjustments to predictable perturbations	Cignetti F, Zedka M, Vaugoyeau M, Assaiante C.	PubMed	PLoS One	2013	10.1371/journal.pone.0056313	excluded	No comparison of arm movement in balance or postural control was conducted
285	Exercise intervention designed to improve strength and dynamic balance among community-dwelling older adults	DiBrezzo R, Shadden BB, Raybon BH, Powers M.	PubMed	J Aging Phys Act	2005	10.1123/japa.13.2.198	excluded	No comparison of arm movement in balance or postural control was conducted

286	Sign Chi Do and physical function: a pilot study	Rogers CE, Nseir S, Keller C.	PubMed	Geriatr Nurs	2013	10.1016/j.gerinurse.2012.05.004	excluded	No comparison of arm movement in balance or postural control was conducted
287	Implications of low mechanical impedance in upper limb reaching motion	Popescu FC, Rymeri WZ.	PubMed	Motor Control	2003	10.1123/mcj.7.4.323	excluded	No comparison of arm movement in balance or postural control was conducted
288	Perceived versus actual head-on-trunk orientation during arm movement control	Guerraz M, Navarro J, Ferrero F, Cremieux J, Blouin J.	PubMed	Exp Brain Res	2006	10.1007/s00221-005-0316-3	excluded	No comparison of arm movement in balance or postural control was conducted
289	Effects of limiting anterior displacement of the center of foot pressure on anticipatory postural control during bilateral shoulder flexion	Fujiwara K, Yaguchi C.	PubMed	J Electromyogr Kinesiol	2013	10.1016/j.jelekin.2013.07.015	excluded	No comparison of arm movement in balance or postural control was conducted
290	Time to disengage: holding an object influences the execution of rapid compensatory reach-to-grasp reactions for recovery from whole-body instability	Van Ooteghem K, Lakhani B, Akram S, Miyasike Da Silva V, McIlroy WE.	PubMed	Exp Brain Res	2013	10.1007/s00221-013-3682-2	excluded	No comparison of arm movement in balance or postural control was conducted
291	Kinematic synergy adaptation to an unstable support surface and equilibrium maintenance during forward trunk movement	Vernazza-Martin S, Martin N, Pellec-Muller AL, Tricon V, Massion J.	PubMed	Exp Brain Res	2006	10.1007/s00221-006-0364-3	excluded	No comparison of arm movement in balance or postural control was conducted

292	Human equilibrium on unstable support: the importance of feet-support interaction	Ivanenko YP, Levik YS, Talis VL, Gurfinkel VS.	PubMed	Neurosci Lett	1997	10.1016/s0304-3940(97)00721-0	excluded	No comparison of arm movement in balance or postural control was conducted
293	Differential control of abdominal muscles during multi-directional support-surface translations in man	Carpenter MG, Tokuno CD, Thorstensson A, Cresswell AG.	PubMed	Exp Brain Res	2008	10.1007/s00221-008-1377-x	excluded	No comparison of arm movement in balance or postural control was conducted
294	Trunk acceleration for neuroprosthetic control of standing: a pilot study	Nataraj R, Audu ML, Kirsch RF, Triolo RJ.	PubMed	J Appl Biomech	2012	10.1123/jab.28.1.85	excluded	No comparison of arm movement in balance or postural control was conducted
295	Slip and Trip Perturbations During an Object Transport Task Requiring a Lateral Change in Support	Huntley AH, Inkol KA, Vallis LA.	PubMed	J Mot Behav	2018	10.1080/00222895.2017.1363696	excluded	No comparison of arm movement in balance or postural control was conducted
296	Validation of net joint loads calculated by inverse dynamics in case of complex movements: application to balance recovery movements	Robert T, Chèze L, Dumas R, Verriest JP.	PubMed	J Biomech	2007	10.1016/j.jbiomech.2006.11.014	excluded	No comparison of arm movement in balance or postural control was conducted
297	Lifting over an obstacle: effects of one-handed lifting and hand support on trunk kinematics and low back loading	Kingma I, van Dieën JH.	PubMed	J Biomech	2004	10.1016/s0021-9290(03)00248-3	excluded	No comparison of arm movement in balance or postural control was conducted
298	Muscle modes during shifts of the center of pressure by standing persons: effect of instability and additional support	Krishnamoorthy V, Latash ML, Scholz JP, Zatsiorsky VM.	PubMed	Exp Brain Res	2004	10.1007/s00221-003-1812-y	excluded	No comparison of arm movement in balance or postural control was conducted

299	Unexperienced mechanical effects of muscular fatigue can be predicted by the Central Nervous System as revealed by anticipatory postural adjustments	Monjo F, Forestier N.	PubMed	Exp Brain Res	2014	10.1007/s00221-014-3975-0	excluded	No comparison of arm movement in balance or postural control was conducted
300	Closed-loop multivariable system identification for the characterization of the dynamic arm compliance using continuous force disturbances: a model study	de Vlugt E, Schouten AC, van der Helm FC.	PubMed	J Neurosci Methods	2003	10.1016/s0165-0270(02)00303-5	excluded	No comparison of arm movement in balance or postural control was conducted
301	Maintenance of upright standing posture during trunk rotation elicited by rapid and asymmetrical movements of the arms	Yamazaki Y, Suzuki M, Ohkuwa T, Itoh H.	PubMed	Brain Res Bull	2005	10.1016/j.brainresbull.2005.05.015	excluded	No comparison of arm movement in balance or postural control was conducted
302	Unintentional movements induced by sequential transient perturbations in a multi-joint positional task	Zhou T, Falaki A, Latash ML.	PubMed	Hum Mov Sci	2016	10.1016/j.humov.2015.12.002	excluded	No comparison of arm movement in balance or postural control was conducted
303	Explanations pertaining to the hip joint flexor moment during the stance phase of human walking	Simonsen EB, Cappelen KL, Skorini Rí, Larsen PK, Alkjær T, Dyhre-Poulsen P.	PubMed	J Appl Biomech	2012	10.1123/jab.28.5.542	excluded	No comparison of arm movement in balance or postural control was conducted
304	Interpreting physical and behavioral health scores from new work disability instruments	Marfeo EE, Ni P, Chan L, Rasch EK, McDonough CM, Brandt DE, Bogusz K, Jette AM.	PubMed	J Rehabil Med	2015	10.2340/16501977-1947	excluded	No comparison of arm movement in balance or postural control was conducted

305	What is balance?	Pollock AS, Durward BR, Rowe PJ, Paul JP.	PubMed	Clin Rehabil	2000	10.1191/0269215500cr342oa	excluded	No comparison of arm movement in balance or postural control was conducted
306	Effects of Balance Training on	Lesinski M, Hortobágyi T, Muehlbauer T, Gollhofer A, Granacher U.	PubMed	Sports Med	2015	10.1007/s40279-015-0375-y	excluded	No comparison of arm movement in balance or postural control was conducted
307	Static balance norms in childr	Condon C, Cremin K.	PubMed	Physiother Res Int	2014	10.1002/pri.1549	excluded	No comparison of arm movement in balance or postural control was conducted
308	Balance control, agility, eye-ha	Wong TKK, Ma AWW, Liu KPY, Chung LMY, Bae YH, Fong SSM, Ganesan B, Wang HK.	PubMed	Medicine (Baltimore)	2019	10.1097/MD.0000000000014134	excluded	No comparison of arm movement in balance or postural control was conducted
309	The effects of the Otago Exerc	Chiu HL, Yeh TT, Lo YT, Liang PJ, Lee SC.	PubMed	PLoS One	2021	10.1371/journal.pone.0255780	excluded	No comparison of arm movement in balance or postural control was conducted
310	**Star excursion balance test s**	**Sogut B, Harput G, Tunay VB.**	**PubMed**	**J Bodyw Mov Ther**	**2022**	**10.1016/j.jbmt.2021.10.011**	**included**	
311	Postural stability in strabismu	Nouraeinejad A.	PubMed	Strabismus	2023	10.1080/09273972.2023.2236138	excluded	No comparison of arm movement in balance or postural control was conducted
312	Maintenance of postural stabi	Dutt-Mazumder A, Challis J, Newell K.	PubMed	Hum Mov Sci	2016	10.1016/j.humov.2016.04.010	excluded	No comparison of arm movement in balance or postural control was conducted
313	Stability of Balance Performar	Blodgett JM, Cooper R, Pinto Pereira SM, Hamer M.	PubMed	Pediatrics	2022	10.1542/peds.2021-055861	excluded	No comparison of arm movement in balance or postural control was conducted
314	The dominant foot affects the	Yoshida T, Ikemiyagi F, Ikemiyagi Y, Tanaka T, Yamamoto M, Suzuki M.	PubMed	Acta Otolaryngol	2014	10.3109/00016489.2014.940556	excluded	No comparison of arm movement in balance or postural control was conducted

315	Kinect-based assessment of l	Eltoukhy M, Kuenze C, Oh J, Wooten S, Signorile J.	PubMed	Gait Posture	2017	10.1016/j.gaitpost.2017.09.010	excluded	No comparison of arm movement in balance or postural control was conducted
316	Postural Control, Dual Task Pe	Smith DL, Haworth JL, Brooks EK, Cousins JM.	PubMed	Percept Mot Skills	2021	10.1177/00315125211044351	excluded	No comparison of arm movement in balance or postural control was conducted
317	The Feasibility of Using the Vir	Lin CC, Kim S, DeVita P, Becker M, Meardon S.	PubMed	Motor Control	2022	10.1123/mc.2021-0076	excluded	No comparison of arm movement in balance or postural control was conducted
318	The test-retest reliability of ce	Ruhe A, Fejer R, Walker B.	PubMed	Gait Posture	2010	10.1016/j.gaitpost.2010.09.012	excluded	No comparison of arm movement in balance or postural control was conducted
319	Neural prosthesis control rest	Fleming A, Liu W, Huang HH.	PubMed	Sci Robot	2023	10.1126/scirobotics.adf5758	excluded	No comparison of arm movement in balance or postural control was conducted
320	Leg Muscle Activity and Joint M	Yamako G, Ito K, Muraoka T, Chosa E.	PubMed	Int J Environ Res Public Health	2023	10.3390/ijerph20020915	excluded	No comparison of arm movement in balance or postural control was conducted
321	Effects of supportive hand cor	Babič J, Petrič T, Peternel L, Šarabon N.	PubMed	Gait Posture	2014	10.1016/j.gaitpost.2014.05.012	excluded	No comparison of arm movement in balance or postural control was conducted
322	Effects of physical training on	Lelard T, Ahmaidi S.	PubMed	Neurophysiol Clin	2015	10.1016/j.neucli.2015.09.008	excluded	No comparison of arm movement in balance or postural control was conducted
323	Greater Star Excursion Balanc	Krityakiarana W, Jongkamonwiwat N.	PubMed	J Dance Med Sci	2022	10.12678/1089-313X.091522a	excluded	No comparison of arm movement in balance or postural control was conducted
324	Neurocognitive function influe	Porter KH, Quintana C, Morelli N, Heebner N, Winters J, Han DY, Hoch M.	PubMed	J Sci Med Sport	2022	10.1016/j.jsams.2021.07.012	excluded	No comparison of arm movement in balance or postural control was conducted

325	Association Between Plantarfl	Magalhães FH, Mello EM, Kohn AF.	PubMed	Somatosens Mot Res	2019	10.1080/08990220.2019.1673720	excluded	No comparison of arm movement in balance or postural control was conducted
326	Postural stability assessment	Piras A, Bertucco M, Del Santo F, Meoni A, Raffi M.	PubMed	J Electromyogr Kinesiol	2024	10.1016/j.jelekin.2023.102855	excluded	No comparison of arm movement in balance or postural control was conducted
327	Effect of motor and sensory nc	Cherif A, Loram I, Zenzeri J.	PubMed	Prog Brain Res	2019	10.1016/bs.pbr.2019.04.031	excluded	No comparison of arm movement in balance or postural control was conducted
328	Perturbation-Based Balance E	Yamamoto M, Shimatani K, Yoshikawa D, Washida T, Takemura H.	PubMed	IEEE J Transl Eng Health Med	2023	10.1109/JTEHM.2023.3310503	excluded	No comparison of arm movement in balance or postural control was conducted
329	Dynamic postural control in in	Zavala P, Vannatta CN, Kernozek TW, Rutherford DN.	PubMed	Gait Posture	2023	10.1016/j.gaitpost.2023.06.002	excluded	No comparison of arm movement in balance or postural control was conducted
330	Relationship between bow sta	Sarro KJ, Viana TC, De Barros RML.	PubMed	Eur J Sport Sci	2021	10.1080/17461391.2020.1754471	excluded	No comparison of arm movement in balance or postural control was conducted
331	The Relationship Between Mea	DeFeo C, Heebner N, Baker C, Hoch M, Morelli N.	PubMed	J Sport Rehabil	2022	10.1123/jsr.2021-0291	excluded	No comparison of arm movement in balance or postural control was conducted
332	Effects of balance exercises d	Muehlbauer T, Waldermann F.	PubMed	Gait Posture	2022	10.1016/j.gaitpost.2021.12.020	excluded	No comparison of arm movement in balance or postural control was conducted
333	Y balance test has no correlati	Almeida GPL, Monteiro IO, Marizeiro DF, Maia LB, de Paula Lima PO.	PubMed	Musculoskelet Sci Pract	2017	10.1016/j.msksp.2016.11.008	excluded	No comparison of arm movement in balance or postural control was conducted
334	Balance capacity influences tl	Kal EC, Young WR, Ellmers TJ.	PubMed	Hum Mov Sci	2022	10.1016/j.humov.2022.102933	excluded	No comparison of arm movement in balance or postural control was conducted

335	Analysis of postural stability fc	Bustamante Valles KD, Long JT, Riedel SA, Graf A, Krzak J, Hassani S, Riordan M, Zaharski K, Sturm PF, Harris GF.	PubMed	Stud Health Technol Inform	2010		excluded	No comparison of arm movement in balance or postural control was conducted
336	**Can arm movements improve**	**Objero CN, Wdowski MM, Hill MW.**	**PubMed**	**Gait Posture**	**2019**	**10.1016/j.gaitpost.2019.08.010**	**included**	
337	The Impact of Viewing Distanc	Teaford M, Mularczyk ZJ, Gernon A, Merfeld DM.	PubMed	Multisens Res	2024	10.1163/22134808-bja10131	excluded	No comparison of arm movement in balance or postural control was conducted
338	Effect of dual-task interaction	Nishimoto R, Fujiwara S, Kutoku Y, Ogata T, Mihara M.	PubMed	Neuroimage	2023	10.1016/j.neuroimage.2023.120352	excluded	No comparison of arm movement in balance or postural control was conducted
339	The influence of age and overv	Andreato LV, de Oliveira DV, Follmer B, Bertolini SMMG.	PubMed	Australas J Ageing	2020	10.1111/ajag.12782	excluded	No comparison of arm movement in balance or postural control was conducted
340	Assessment of dynamic balan	Eltoukhy M, Kuenze C, Jun HP, Asfour S, Travascio F.	PubMed	Sports Biomech	2015	10.1080/14763141.2015.1025238	excluded	No comparison of arm movement in balance or postural control was conducted
341	Can a Balance Wristband Influ	Eichhorn S, Foerster S, Friemert B, Willy C, Riesner HJ, Palm HG.	PubMed	J Strength Cond Res	2020	10.1519/JSC.0000000000002091	excluded	No comparison of arm movement in balance or postural control was conducted
342	Healthy aging reduces dynami	Segal AD, Vargas BL, Richards FG, Shelley CJ, Silverman AK.	PubMed	Gait Posture	2023	10.1016/j.gaitpost.2023.05.020	excluded	No comparison of arm movement in balance or postural control was conducted
343	Dual-Task Conditions on Stati	Petrigna L, Gentile A, Mani D, Pajaujiene S, Zanotto T, Thomas E, Paoli A, Palma A, Bianco A.	PubMed	J Aging Phys Act	2021	10.1123/japa.2019-0474	excluded	No comparison of arm movement in balance or postural control was conducted

344	Changes in Postural Control A	Yu DS, Kim SY.	PubMed	Int J Environ Res Public Health	2022	10.3390/ijerph19116643	excluded	No comparison of arm movement in balance or postural control was conducted
345	Validation of Dance-Specific E	Johari MR, Kee YH, Kong PW.	PubMed	Motor Control	2022	10.1123/mc.2021-0116	excluded	No comparison of arm movement in balance or postural control was conducted
346	Ankle muscles activation and	Karagiannakis DN, Iatridou KI, Mandalidis DG.	PubMed	Hum Mov Sci	2020	10.1016/j.humov.2019.102563	excluded	No comparison of arm movement in balance or postural control was conducted
347	The effect of latin dance on dy	Kiliç M, Nalbant SS.	PubMed	Gait Posture	2022	10.1016/j.gaitpost.2021.11.037	excluded	No comparison of arm movement in balance or postural control was conducted
348	Determination of the Predicto	Chung CM, Shin S, Lee Y, Lee DY.	PubMed	Medicina (Kaunas)	2022	10.3390/medicina58111640	excluded	No comparison of arm movement in balance or postural control was conducted
349	Determinants of sport-specifi	Chow GC, Fong SS, Chung JW, Chung LM, Ma AW, Macfarlane DJ.	PubMed	J Sci Med Sport	2016	10.1016/j.jsams.2016.02.016	excluded	No comparison of arm movement in balance or postural control was conducted
350	Postural control strategies are	Moreno FJ, Caballero C, Barbado D.	PubMed	J Neurophysiol	2022	10.1152/jn.00426.2021	excluded	No comparison of arm movement in balance or postural control was conducted
351	The impact of anxiety on postu	Taylor ANW, Low DC, Walsh GS, Holt N.	PubMed	Psychophysiology	2023	10.1111/psyp.14192	excluded	No comparison of arm movement in balance or postural control was conducted
352	Maturation of the postural con	Błaszczyk JW, Fredyk A.	PubMed	Gait Posture	2021	10.1016/j.gaitpost.2020.10.036	excluded	No comparison of arm movement in balance or postural control was conducted
353	Optimal exercise parameters (	Wang LC, Ye MZ, Xiong J, Wang XQ, Wu JW, Zheng GH.	PubMed	J Am Geriatr Soc	2021	10.1111/jgs.17094	excluded	No comparison of arm movement in balance or postural control was conducted

354	Effect of gaze-stabilization exe	Matsugi A, Ueta Y, Oku K, Okuno K, Tamaru Y, Nomura S, Tanaka H, Mori N.	PubMed	Neuroreport	2017	10.1097/WNR.0000000000000776	excluded	No comparison of arm movement in balance or postural control was conducted
355	Postural Control and Psychopl	Polak E, Ślugaj R, Gardzińska A.	PubMed	Front Public Health	2022	10.3389/fpubh.2022.788612	excluded	No comparison of arm movement in balance or postural control was conducted
356	Cross-Education Balance Effe	Lawry-Popelka B, Chung S, McCann RS.	PubMed	J Athl Train	2022	10.4085/1062-6050-625-21	excluded	No comparison of arm movement in balance or postural control was conducted
357	Postural control and functiona	Rein S, Fabian T, Zwipp H, Rammelt S, Weindel S.	PubMed	Clin Neurophysiol	2011	10.1016/j.clinph.2011.01.004	excluded	No comparison of arm movement in balance or postural control was conducted
358	Effect of balance training on st	Muehlbauer T, Grundmann A, Vortkamp L, Schedler S.	PubMed	BMC Res Notes	2022	10.1186/s13104-022-06177-y	excluded	No comparison of arm movement in balance or postural control was conducted
359	Children's head movements a	Flatters I, Mushtaq F, Hill LJ, Rossiter A, Jarrett-Peet K, Culmer P, Holt R, Wilkie RM, Mon-Williams M.	PubMed	Exp Brain Res	2014	10.1007/s00221-014-3886-0	excluded	No comparison of arm movement in balance or postural control was conducted
360	Effect of Exergame Interventio	Zhang C, Han T, Tan X, Yu C, Li S, Zheng H, Zhu D, Zhang Y, Shen T.	PubMed	Games Health J	2023	10.1089/g4h.2022.0182	excluded	No comparison of arm movement in balance or postural control was conducted
361	How can the stimulation of pla	Viseux F, Lemaire A, Barbier F, Charpentier P, Leteneur S, Villeneuve P.	PubMed	Neurophysiol Clin	2019	10.1016/j.neucli.2018.12.006	excluded	No comparison of arm movement in balance or postural control was conducted

362	The Effect of Balance and San	Sebastia-Amat S, Ardigò LP, Jimenez-Olmedo JM, Pueo B, Penichet-Tomas A.	PubMed	Int J Environ Res Public Health	2020	10.3390/ijerph17238981	excluded	No comparison of arm movement in balance or postural control was conducted
363	The role of spatial alignment ir	Mitra S, Vernon M, Boulton H.	PubMed	Gait Posture	2022	10.1016/j.gaitpost.2022.01.011	excluded	No comparison of arm movement in balance or postural control was conducted
364	External load training does not	Simpson JD, Miller BL, O'Neal EK, Chander H, Knight AC.	PubMed	Sports Biomech	2018	10.1080/14763141.2017.1341546	excluded	No comparison of arm movement in balance or postural control was conducted
365	Assessment of Balance Contr	Ren P, Huang S, Feng Y, Chen J, Wang Q, Guo Y, Yuan Q, Yao D, Ma D.	PubMed	IEEE Trans Neural Syst Rehabil Eng	2020	10.1109/TNSRE.2020.2966784	excluded	No comparison of arm movement in balance or postural control was conducted
366	Improvement of anticipatory p	Kanekar N, Aruin AS.	PubMed	J Electromyogr Kinesiol	2015	10.1016/j.jelekin.2014.11.002	excluded	No comparison of arm movement in balance or postural control was conducted
367	Slackline Training (Balancing (	Donath L, Roth R, Zahner L, Faude O.	PubMed	Sports Med	2017	10.1007/s40279-016-0631-9	excluded	No comparison of arm movement in balance or postural control was conducted
368	Effects of age and surface inst	van den Bogaart M, Bruijn SM, Spildooren J, van Dieën JH, Meyns P.	PubMed	Hum Mov Sci	2022	10.1016/j.humov.2022.102930	excluded	No comparison of arm movement in balance or postural control was conducted
369	Learning effect of dynamic pos	Keklicek H, Kırdı E, Yalcin A, Yuce D, Topuz S.	PubMed	J Back Musculoskelet Rehabil	2019	10.3233/BMR-181172	excluded	No comparison of arm movement in balance or postural control was conducted
370	Y-Balance Test Performance F	Hoch MC, Welsch LA, Hartley EM, Powden CJ, Hoch JM.	PubMed	J Sport Rehabil	2017	10.1123/jsr.2017-0004	excluded	No comparison of arm movement in balance or postural control was conducted
371	Computerized dynamic postu	Palm HG, Lang P, Strobel J, Riesner HJ, Friemert B.	PubMed	Am J Phys Med Rehabil	2014	10.1097/PHM.0b013e3182a39019	excluded	No comparison of arm movement in balance or postural control was conducted

372	Sample entropy discriminates	Workman CD, Sosnoff JJ, Rudroff T.	PubMed	Clin Biomech (Bristol)	2022	10.1016/j.clinbiomech.2022.105593	excluded	No comparison of arm movement in balance or postural control was conducted
373	Driving evaluation methods for able-bodied persons and individuals with lower extremity disabilities: a review of assessment modalities	Greve JM, Santos L, Alonso AC, Tate DG.	SPORTDiscus	Clinics (Sao Paulo)	2015	10.6061/clinics/2015(09)08	excluded	No comparison of arm movement in balance or postural control was conducted
374	Electronic Patient-Reported Outcome Validation: Disablement in the Physically Active Scale	Stankevitz D, Larkins L, Baker RT.	SPORTDiscus	J Athl Train	2019	10.4085/1062-6050-420-17	excluded	No comparison of arm movement in balance or postural control was conducted
375	A 10-year population-based study of people with multiple sclerosis in Stockholm, Sweden: use of and satisfaction with care and the value of different factors in predicting use of care	Chruzander C, Johansson S, Gottberg K, Einarsson U, Hillert J, Holmqvist LW, Ytterberg C.	SPORTDiscus	BMC Health Serv Res	2015	10.1186/s12913-015-1144-1	excluded	No comparison of arm movement in balance or postural control was conducted
376	Longitudinal Trends of Participation in Relation to Mental Health in Children with and without Physical Difficulties	Hwang AW, Chang CH, Granlund M, Imms C, Chen CL, Kang LJ.	SPORTDiscus	Int J Environ Res Public Health	2020	10.3390/ijerph17228551	excluded	No comparison of arm movement in balance or postural control was conducted
377	Do personal assistance activities promote participation for persons with disabilities in Sweden?	von Granitz H, Reine I, Sonnander K, Winblad U.	SPORTDiscus	Disabil Rehabil	2017	10.1080/09638288.2016.1236405	excluded	No comparison of arm movement in balance or postural control was conducted

378	Common impairments and functional limitations of HIV sequelae that require physiotherapy rehabilitation in the medical wards at Queen Elizabeth Central Hospital, Malawi: A cross sectional study	Banda GT, Mwale G, Chimwala M, Malimusi L, Chisati E.	SPORTDiscus	Malawi Med J	2019	10.4314/mmj.v31i3.2	excluded	No comparison of arm movement in balance or postural control was conducted
379	Effects of Disability Type on the Association between Age and Non-Communicable Disease Risk Factors among Elderly Persons with Disabilities in Shanghai, China	Wang X, Sun M, Li X, Lu J, Chen G.	SPORTDiscus	Int J Environ Res Public Health	2020	10.3390/ijerph17155426	excluded	No comparison of arm movement in balance or postural control was conducted
380	Eligibility for Supplemental Service-Disabled Veterans' Insurance. Final rule	Department of Veterans Affairs.	SPORTDiscus	Fed Regist	2018		excluded	No comparison of arm movement in balance or postural control was conducted
381	Deconstruction of physical fitness assessment system and medical rehabilitation countermeasures for physically disabled teenagers with natural language processing technology	Wang D, Sun P.	SPORTDiscus	Front Public Health	2022	10.3389/fpubh.2022.964030	excluded	No comparison of arm movement in balance or postural control was conducted
382	Effect of COVID-19 on Internet Usage of People with Disabilities: A Secondary Data Analysis	Park EY.	SPORTDiscus	Int J Environ Res Public Health	2022	10.3390/ijerph19137813	excluded	No comparison of arm movement in balance or postural control was conducted

383	Shut up, or Set Free: Poetic Inquiry into Disabled Students' Experiences of Differential Attainment	Brown MEL, Finn G.	SPORTDiscus	Perspect Med Educ	2024	10.5334/pme.1392	excluded	No comparison of arm movement in balance or postural control was conducted
384	The first world war drives rehabilitation toward the modern concepts of disability and participation	Bonfiglioli Stagni S, Tomba P, Viganò A, Zati A, Benedetti MG.	SPORTDiscus	Eur J Phys Rehabil Med	2015		excluded	No comparison of arm movement in balance or postural control was conducted
385	The multidimensional attitudes scale towards persons with disabilities (MAS) - a Polish adaptation (MAS-PL)	Radlińska I, Starkowska A, Kożybska M, Flaga-Gieruszyńska K, Karakiewicz B.	SPORTDiscus	Ann Agric Environ Med	2020	10.26444/aaem/114531	excluded	No comparison of arm movement in balance or postural control was conducted
386	Use of Vocational Rehabilitation Supports for Postsecondary Education Among Transition-Age Youth on the Autism Spectrum	Rast JE, Roux AM, Shattuck PT.	SPORTDiscus	J Autism Dev Disord	2020	10.1007/s10803-019-03972-8	excluded	No comparison of arm movement in balance or postural control was conducted
387	Improving static balance ability with trainings supported by somatosensory-based feedback system.	Wang, Wei ; Wang, Wenjing ; Shadiev, Rustam	SPORTDiscus	Smart Learning Environments	2022	10.1186/s40561-022-00216-8	excluded	No comparison of arm movement in balance or postural control was conducted
388	Effects of Nontraditional Division III Lacrosse Participation on Movement Pattern Quality and Dynamic Postural Control.	Rosenborough, Christopher ; Collins, Sean M. ; Smith, Edward ; Bowman, Thomas G.	SPORTDiscus	International Journal of Sports Physical Therapy	2024	10.26603/001c.115423	excluded	No comparison of arm movement in balance or postural control was conducted

389	Subtalar joint pronation: Which is the real concern-presence or severity? A cross-sectional study.	Pişirici, Pelin ; Feyzioğlu, Özlem ; Kaygas, Nurefşan ; Mollaibrahimoğlu, Yahya Süleyman	SPORTDiscus	Turkish Journal of Kinesiology	2024	10.31459/turkjkin.1535023	excluded	No comparison of arm movement in balance or postural control was conducted
390	The effect of different jaw positions on upper extremity performance, core endurance, and postural stability: A cross-sectional study.	Demirdel, Senem ; Gül, Gülşah ; Gümüş, Öznur ; Kuz, Betül	SPORTDiscus	Turkish Journal of Kinesiology	2023	10.31459/turkjkin.1232047	excluded	No comparison of arm movement in balance or postural control was conducted
391	Neuromuscular but Not Technical Performance is Affected by Time-of-Day in Semiprofessional, Female Basketball Players.	Gaos, Sofía ; Sánchez-Jorge, Sandra ; Muñoz, Alejandro ; Vicente-Campos, Davinia ; Acebes-Sánchez, Jorge ; Esquius, Laura ; Scanlan, Aaron T. ; López-Samanes, Álvaro	SPORTDiscus	Research Quarterly for Exercise & Sport	2024	10.1080/02701367.2023.2265447	excluded	No comparison of arm movement in balance or postural control was conducted
392	Postural control during gait termination and prehension.	Jeong, Hwigeum ; Cabiles, Natalie ; van Emmerik, Richard E.A.	SPORTDiscus	Gait & Posture	2024	10.1016/j.gaitpost.2024.04.020	excluded	No comparison of arm movement in balance or postural control was conducted
393	IMU Data-Driven and PCA-Based Approach to Establish Quantifiable and Practically Applicable Measures for V2 Technique Elements in Cross-Country Skiing.	Debertin, Daniel ; Haag, Luisa ; Federolf, Peter	SPORTDiscus	Scandinavian Journal of Medicine & Science in Sports	2024	10.1111/sms.14691	excluded	No comparison of arm movement in balance or postural control was conducted

394	Association between core strength and dynamic balance of throwing hand in professional healthy cricket fast bowlers: A cross sectional study.	Krishna, Vidhya ; Noronha, Thrishala ; Pathak, Anupama Anand	SPORTDiscus	Journal of Bodywork & Movement Therapies	2024	10.1016/j.jbmt.2024.02.045	excluded	No comparison of arm movement in balance or postural control was conducted
395	Stability, performance and upper and lower extremities range of motion in elite beach handball athletes: A cross sectional study.	Barbosa, Germanna M. ; Saccol, Michele F. ; Pinheiro, Scheila M. ; Costa, Ítalo D.S. ; Camargo, Paula R. ; Scattone Silva, Rodrigo	SPORTDiscus	Journal of Bodywork & Movement Therapies	2023	10.1016/j.jbmt.2023.04.023	excluded	No comparison of arm movement in balance or postural control was conducted
396	Upper-Extremity physical performance tests in older adults: Reference values, reliability and measurement error.	Novais, Maria Eduarda Oliveira ; Oliveira, Anamaria Siriani de ; Moreira, Rayanne de Paula ; Barbosa, Glauber Marques Paraizo ; Lemos, Thiago Vilela ; Matheus, João Paulo Chieregato ; de Souza Júnior, José Roberto	SPORTDiscus	Journal of Bodywork & Movement Therapies	2024	10.1016/j.jbmt.2024.10.007	excluded	No comparison of arm movement in balance or postural control was conducted
397	Investigation of the Effect of Push-Up Exercises with and without Suspension on Some Motor Skills Applied to Young Volleyball Athletes.	ERİŞ, Fatih	SPORTDiscus	Journal of Education & Recreation Patterns (JERP)	2023	10.53016/jerp.v4i2.169	excluded	No comparison of arm movement in balance or postural control was conducted

398	Relationships between balance and physical fitness variables in firefighter recruits.	Marciniak, Rudi A. ; Ebersole, Kyle T. ; Cornell, David J.	SPORTDiscus	Work	2021	10.3233/WOR-203401	excluded	No comparison of arm movement in balance or postural control was conducted
399	Validity and reliability of upper extremity star excursion balance test in adolescent swimmers.	Xu, Hao-Ran ; Zhang, Yong-Hui ; Mao, Yuan ; Ngo, Thanh Luan ; Zhang, Qiong ; He, Gang ; Feng, Zhimin ; Sun, Wenjia ; Wang, Xue-Qiang	SPORTDiscus	Journal of Exercise Science & Fitness	2023	10.1016/j.jesf.2023.02.003	excluded	No comparison of arm movement in balance or postural control was conducted
400	Motor alterations along the kinetic chain in amateur volleyball and handball athletes with shoulder pain: An observational comparative study.	Silva Barros, Bianca Rodrigues da ; Barros, Alef Cavalcanti Matias de ; da Silva Júnior, Nilton ; Cavalcanti, Isadora Braga Silva ; Sousa, Catarina de Oliveira	SPORTDiscus	Journal of Bodywork & Movement Therapies	2024	10.1016/j.jbmt.2024.02.002	excluded	No comparison of arm movement in balance or postural control was conducted
401	Effect of Carrying Objects on Walking Characteristics and Language Abilities in 13- and 24-Month-Olds.	Arnold, Amanda J. ; Claxton, Laura J.	SPORTDiscus	Developmental Psychology	2023	10.1037/dev0001535	excluded	No comparison of arm movement in balance or postural control was conducted
402	Comparing shoulder proprioception, upper extremity dynamic stability, and hand grip strength in overhead athletes with and without scapular dyskinesis.	Reyhani, Fatemeh ; Meftahi, Narges ; Rojhani-Shirazi, Zahra	SPORTDiscus	Journal of Bodywork & Movement Therapies	2024	10.1016/j.jbmt.2024.03.001	excluded	No comparison of arm movement in balance or postural control was conducted

403	The associations of physical parameters with the Closed Kinetic Chain Upper Extremity Stability Test, the Upper Quarter Y Balance Test, and the Upper Limb Rotation Test in professional overhead athletes.	Kara, Fırat ; Ergin Gedik, Gülbin ; Şahinoğlu, Ertan	SPORTDiscus	Physical Therapy in Sport	2024	10.1016/j.ptsp.2024.03.001	excluded	No comparison of arm movement in balance or postural control was conducted
404	Effects of structured training on spinal posture and selective motor control in children with unilateral spastic cerebral palsy.	Taş, Seda Ayaz ; Çankaya, Tamer	SPORTDiscus	Gait & Posture	2024	10.1016/j.gaitpost.2024.01.007	excluded	No comparison of arm movement in balance or postural control was conducted
405	Effects of core stability and feedback music on upper body mediolateral movements during cycling.	Jeong, Siwoo ; Kim, Si-hyun ; Park, Kyue-nam	SPORTDiscus	BMC Sports Science, Medicine & Rehabilitation	2024	10.1186/s13102-024-00822-8	excluded	No comparison of arm movement in balance or postural control was conducted
406	The Impact of Dance-Specific Neuromuscular Conditioning and Injury Prevention Training on Motor Control, Stability, Balance, Function and Injury in Professional Ballet Dancers: A Mixed-Methods Quasi-Experimental Study.	Long, Katherine L. ; Milidonis, Mary K. ; Wildermuth, Veronica L. ; Kruse, Adam N. ; Parham, Uniqua T.	SPORTDiscus	International Journal of Sports Physical Therapy	2021	10.26603/001c.21150	excluded	No comparison of arm movement in balance or postural control was conducted

407	Transhumeral prosthesis use and disuse affects whole-body angular momentum.	Dunn, Julia A. ; Gomez, Nicholas G. ; Wong, Bob ; Sinclair, Sarina K. ; Foreman, K. Bo ; Bachus, Kent N. ; Henninger, Heath B.	SPORTDiscus	Clinical Biomechanics	2024	10.1016/j.clinbiomech.2024.106365	excluded	No comparison of arm movement in balance or postural control was conducted
408	Upper Lımb Related Factors In Determınıng Postural Control	Kodak, Muhammed Ihsan ; Ozudoğru, Anıl ; Ozsoy, Ismail	SPORTDiscus	Kinesiologia Slovenica	2021	10.52165/kinsi.27.1.121-134	excluded	No comparison of arm movement (free vs restricted)
409	Lower Extremity Musculoskeletal Injuries After Concussion in Collegiate Student-Athletes.	Buckley, Thomas A. ; Chandran, Avinash ; Mauntel, Timothy C. ; Kerr, Zachary Yukio ; Brown, Derek W. ; Boltz, Adrian J. ; Herman, Daniel C. ; Hall, Eric E. ; Lynall, Robert C.	SPORTDiscus	American Journal of Sports Medicine	2023	10.1177/03635465221125155	excluded	No comparison of arm movement in balance or postural control was conducted
410	The interactions between agonist-to-antagonist muscle strength performance and plantar pressure distribution, foot contact area, and impulse in novice ballet dancers.	Arinli, Yağmur ; Umutlu, Gökhan ; Pehlevan, Zekai	SPORTDiscus	Journal of Back & Musculoskeletal Rehabilitation	2023	10.3233/BMR-220406	excluded	No comparison of arm movement in balance or postural control was conducted
411	Enhancing motor fitness in the elderly: The impact of structured functional training program.	SARMAH, BHARGAV ; VIDHATE, S. S. ; AUTADE, SANDIPRAJ ; BALO, ABHISHEK ; SINGH, TANVI	SPORTDiscus	Journal of Physical Education & Sport	2024	10.7752/jpes.2024.05141	excluded	No comparison of arm movement in balance or postural control was conducted

412	Lower extremity movement quality in professional team sport athletes: Inter-rater agreement and relationships with quantitative results from the corresponding pattern.	Keller, Matthias ; Niederer, Daniel ; Schwesig, René ; Kurz, Eduard	SPORTDiscus	BMC Sports Science, Medicine & Rehabilitation	2024	10.1186/s13102-024-00886-6	excluded	No comparison of arm movement in balance or postural control was conducted
413	The Effects of a Novel Quadrupedal Movement Training Program on Functional Movement, Range of Motion, Muscular Strength, and Endurance.	Buxton, Jeffrey D. ; Prins, Philp J. ; Miller, Michael G. ; Moreno, Anthony ; Welton, Gary L. ; Atwell, Adam D. ; Talampas, Tirzah R. ; Elsey, Gretchen E.	SPORTDiscus	Journal of Strength & Conditioning Research	2022	10.1519/JSC.0000000000003818	excluded	No comparison of arm movement in balance or postural control was conducted
414	A Dual Inertial Measurement Unit System for Classifying Standard Overhead Drill Movements in Elite Women's Water Polo.	KING, MARGUERITE H. ; LEWIS, AMY ; WATSON, KATE ; COSTA, NATHALIA ; VICENZINO, BILL	SPORTDiscus	Medicine & Science in Sports & Exercise	2024	10.1249/MSS.0000000000003369	excluded	No comparison of arm movement in balance or postural control was conducted
415	Effects of grouped versus alternating functional training on the shoulder girdle and lumbar-pelvic girdle stability: a randomised controlled trial.	Silva-Grigoletto, Marzo E. Da ; Aragão-Santos, José C. ; Fontes, Alan S. ; Santos, Marta S. ; Resende-Neto, Antônio G. ; Monteiro, Marcos Raphael Pereira ; Cyrino, Edilson S. ; Marin, Pedro J. ; Behm, David G.	SPORTDiscus	Motricidade	2022	10.6063/motricidade.27292	excluded	No comparison of arm movement in balance or postural control was conducted

416	Female Collegiate Dancers' Physical Fitness across Their Four-Year Programs: A Prospective Analysis.	Ambegaonkar, Jatin P. ; Hansen-Honeycutt, Jena ; Wiese, Kelley R. ; Cavanagh, Catherine M. ; Caswell, Shane V. ; Ambegaonkar, Shruti J. ; Martin, Joel	SPORTDiscus	Journal of Functional Morphology & Kinesiology	2023	10.3390/jfmk8030098	excluded	No comparison of arm movement in balance or postural control was conducted
417	Immediate Effects of Limb Rotational Kinesio Tape Application on Upper Quarter Y-Balance Test Scores.	Dittmer, Alyssa ; Tomchuk, David ; Fontenot, David R.	SPORTDiscus	Journal of Sport Rehabilitation	2021	10.1123/jsr.2019-0145	excluded	No comparison of arm movement in balance or postural control was conducted
418	Association between the Upper Quarter Dynamic Balance, Anthropometrics, Kinematics, and Swimming Speed.	Bartolomeu, Raul F. ; Sampaio, Tatiana ; Oliveira, João P. ; Barbosa, Tiago M. ; Morais, Jorge E.	SPORTDiscus	Journal of Functional Morphology & Kinesiology	2023	10.3390/jfmk8030096	excluded	No comparison of arm movement in balance or postural control was conducted
419	Analysis of the movements of the upper extremities during gait: Their role for the dynamic balance.	Matuszewska, Agata ; Syczewska, Małgorzata	SPORTDiscus	Gait & Posture	2023	10.1016/j.gaitpost.2022.12.004	excluded	No comparison of arm movement (free vs restricted)
420	Intra- and inter-rater reliability of limb length measurement and trial error assessment of the Upper Quarter Y-Balance Test in healthy adults.	Williamson, Joshua D. ; Lawson, Braden L. ; Sigley, Daniel ; Nasypany, Alan ; Baker, Russell T.	SPORTDiscus	International Journal of Sports Physical Therapy	2019	10.26603/ijspt20190707	excluded	No comparison of arm movement in balance or postural control was conducted

421	The Relationship Between Functional Movement, Dynamic Stability, and Athletic Performance Assessments in Baseball and Softball Athletes.	Stapleton, Drue T. ; Boergers, Richard J. ; Rodriguez, Johnny ; Green, Gerard ; Johnson, Kiarrah ; Williams, Perry ; Leelum, Nicholas ; Jackson, Lomenee ; Vallorosi, Jessie	SPORTDiscus	Journal of Strength & Conditioning Research	2021	10.1519/JSC.0000000000003781	excluded	No comparison of arm movement in balance or postural control was conducted
422	Postural control and physiological responses to a simulated match in U-20 judo competitors.	Santos, Luis ; Fernández-Río, Javier ; Iglesias-Soler, Eliseo ; Blanco-Traba, Miguel ; Jakobsen, Markus Due ; González-Díez, Vicente ; Franchini, Emerson ; Gutiérrez, Carlos ; Dopico-Calvo, Xurxo ; Carballeira-Fernández, Eduardo ; Amonette, William ; Suman, Oscar	SPORTDiscus	Sports Biomechanics	2020	10.1080/14763141.2018.1461237	excluded	No comparison of arm movement in balance or postural control was conducted
423	Anthropometric and Physiological Predictors of Soccer Skills in Youth Soccer Players	Sanpasitt, Chanawat ; Yongtawee, Atcharat ; Noikhammueang, Thitiwat ; Likhitworasak, Daranee ; Minjung Woo	SPORTDiscus	Physical Education Theory & Methodology	2023	10.17309/tmfv.2023.5.04	excluded	No comparison of arm movement in balance or postural control was conducted
424	Biomechanics of core musculature on upper extremity performance in basketball players.	Arora, Chandrakala ; Singh, Piyush ; Varghese, Vicky	SPORTDiscus	Journal of Bodywork & Movement Therapies	2021	10.1016/j.jbmt.2021.02.023	excluded	No comparison of arm movement in balance or postural control was conducted

425	The Effect of Additional Leg Supports in Control of Posture in Sitting.	Ademiluyi, Adeolu ; Liang, Huaqing ; Aruin, Alexander S.	SPORTDiscus	Journal of Motor Behavior	2023	10.1080/00222895.2023.2181751	excluded	No comparison of arm movement in balance or postural control was conducted
426	Analysis of the level of swimming physical training based on limb strength	Zhang Xin	SPORTDiscus	Revista Brasileira de Medicina do Esporte	2023	10.1590/1517-8692202329012022_0549	excluded	No comparison of arm movement in balance or postural control was conducted
427	The Effects of Constraining Head Rotation on Eye and Whole-Body Coordination During Standing Turns at Different Speeds.	Hollands, Mark ; Khobkhun, Fuengfa ; Ajjimaporn, Amornpan ; Robins, Rebecca ; Richards, Jim	SPORTDiscus	Journal of Applied Biomechanics	2022	10.1123/jab.2021-0117	excluded	No comparison of arm movement in balance or postural control was conducted
428	Factors affecting the shoulder functional profile in elite judo athletes.	Delorme, Julien ; Blache, Yoann ; Degot, Matthieu ; Rogowski, Isabelle	SPORTDiscus	European Journal of Sport Science	2023	10.1080/17461391.2022.2069511	excluded	No comparison of arm movement in balance or postural control was conducted
429	The Immediate Effects of Self-Myofacial Release on Flexibility, Jump Performance and Dynamic Balance Ability.	Zhang, Qingshan ; Trama, Robin ; Fouré, Alexandre ; Hautier, Christophe A	SPORTDiscus	Journal of Human Kinetics	2021	10.2478/hukin-2020-0043	excluded	No comparison of arm movement in balance or postural control was conducted
430	Effects of upper limb loss and prosthesis use on proactive mechanisms of locomotor stability.	Major, Matthew J. ; McConn, Suzanne M. ; Zavaleta, José Luis ; Stine, Rebecca ; Gard, Steven A.	SPORTDiscus	Journal of Electromyography & Kinesiology	2019	10.1016/j.jelekin.2019.07.012	excluded	No comparison of arm movement in balance or postural control was conducted
431	Examining Fundamental Movement Competency and Closed-Chain Upper-Extremity Dynamic Balance in Swimmers	BULLOCK, GARRETT S. ; BROOKRESON, NATE ; KNAB, AMY M. ; BUTLER, ROBERT J.	SPORTDiscus	Journal of Strength & Conditioning Research	2017	10.1519/JSC.0000000000001627	excluded	No comparison of arm movement in balance or postural control was conducted

432	**The effect of various arm and walking conditions on postural dynamic stability when recovering from a trip perturbation.**	**Gholizadeh, Hossein ; Hill, Allen ; Nantel, Julie**	**SPORTDiscus**	**Gait & Posture**	**2020**	**10.1016/j.gaitpost.2019.11.010**	**included**	
433	Normative reference values for handgrip strength, shoulder and ankle range of motion and upper-limb and lower limb stability for 137 youth judokas of both sexes.	Madaleno, Fernanda O. ; Verhagen, Evert ; Ferreira, Thiago V. ; Ribeiro, Tainá ; Ocarino, Juliana M. ; Resende, Renan A.	SPORTDiscus	Journal of Science & Medicine in Sport	2021	10.1016/j.jsams.2020.06.008	excluded	No comparison of arm movement in balance or postural control was conducted
434	Postural stability, clicker reaction time and bow draw force predict performance in elite recurve archery.	Spratford, Wayne ; Campbell, Rhiannon	SPORTDiscus	European Journal of Sport Science	2017	10.1080/17461391.2017.1285963	excluded	No comparison of arm movement in balance or postural control was conducted
435	Influence of typical handball characteristics on upper body posture and postural control in male handball players.	Ohlendorf, D. ; Salzer, S. ; Haensel, R. ; Rey, J. ; Maltry, L. ; Holzgreve, F. ; Lampe, J. ; Wanke, E. M. ; Groneberg, D. A.	SPORTDiscus	BMC Sports Science, Medicine & Rehabilitation	2020	10.1186/s13102-020-0156-2	excluded	No comparison of arm movement in balance or postural control was conducted
436	Learning to balance on a slackline: Development of coordinated multi-joint synergies.	Mildren, R. L. ; Zaback, M. ; Adkin, A. L. ; Bent, L. R. ; Frank, J. S.	SPORTDiscus	Scandinavian Journal of Medicine & Science in Sports	2018	10.1111/sms.13208	excluded	No comparison of arm movement in balance or postural control was conducted
437	Center of pressure velocity-related measures characterize differences in postural control during reaching.	Galgon, Anne K. ; Shewokis, Patricia A. ; Tucker, Carole A.	SPORTDiscus	Journal of Sport & Exercise Psychology	2007		excluded	No comparison of arm movement in balance or postural control was conducted

438	Use of unstable exercises in periscapular muscle activity: A systematic review and meta-analysis of electromyographic studies.	Cappato de Araújo, Rodrigo ; Andrade da Silva, Hítalo ; Pereira dos Passos, Muana Hiandra ; Alves de Oliveira, Valéria Mayaly ; Rodarti Pitangui, Ana Carolina	SPORTDiscus	Journal of Bodywork & Movement Therapies	2021	10.1016/j.jbmt.2020.12.010	excluded	No comparison of arm movement in balance or postural control was conducted
439	Upper Quarter Y-Balance Test in Collegiate Softball Players: Bilateral Arm Comparison and Influence of Reach Order.	Christian, Katelyn M. ; Moran, Matthew F.	SPORTDiscus	International Journal of Athletic Therapy & Training	2021	10.1123/ijatt.2020-0064	excluded	No comparison of arm movement in balance or postural control was conducted
440	Postural balance and oculomotor control are influenced by neck kinaesthetic functions in elite ice hockey players.	Majcen Rosker, Ziva ; Kristjansson, Eythor ; Vodicar, Miha ; Rosker, Jernej	SPORTDiscus	Gait & Posture	2021	10.1016/j.gaitpost.2021.01.024	excluded	No comparison of arm movement in balance or postural control was conducted
441	The effects of short-term hypoxia on upper body isometric strength and reaction time	Assaf, Girgis Kalim ; Kuzmanović, Jovan ; Vučković, Vojko ; Ružić, Lana	SPORTDiscus	Croatian Sports Medicine Journal / Hrvatski sportskome diciniski vjesnik	2021		excluded	No comparison of arm movement in balance or postural control was conducted

442	Effects of strength training with elastic band programme on fitness components in young female handball players: a randomized controlled trial.	Hammami, Mehrez ; Gaamouri, Nawel ; Wagner, Herbert ; Pagaduan, Jeffrey C. ; Hill, Lee ; Nikolaidis, Pantelis T. ; Knechtle, Beat ; Chelly, Mohamed Souhaiel	SPORTDiscus	Biology of Sport	2022	10.5114/biolsport.2022.106390	excluded	No comparison of arm movement in balance or postural control was conducted
443	The Effect of Fatigue on Upper Quarter Y-Balance Test Scores in Recreational Weightlifters: A Randomized Controlled Trial	Salo, Trenton D. ; Chaconas, Eric	SPORTDiscus	International Journal of Sports Physical Therapy	2017		excluded	No comparison of arm movement in balance or postural control was conducted
444	Repeated Exposure to Forward Support-Surface Perturbation During Overground Walking Alters Upper-Body Kinematics and Step Parameters.	Inkol, Keaton A. ; Huntley, Andrew H. ; Vallis, Lori Ann	SPORTDiscus	Journal of Motor Behavior	2019	10.1080/00222895.2018.1474336	excluded	No comparison of arm movement in balance or postural control was conducted
445	Muscle force adaptation to changes in upper body position during seated sprint cycling.	Bini, Rodrigo Rico ; Daly, Luke ; Kingsley, Michael	SPORTDiscus	Journal of Sports Sciences	2019	10.1080/02640414.2019.1627983	excluded	No comparison of arm movement in balance or postural control was conducted
446	Quantifying Segmental Contributions to Center-of-Mass Motion During Dynamic Continuous Support Surface Perturbations Using Simplified Estimation Models.	Schinkel-Ivy, Alison ; Komisar, Vicki ; Duncan, Carolyn A.	SPORTDiscus	Journal of Applied Biomechanics	2020	10.1123/jab.2019-0239	excluded	No comparison of arm movement (free vs restricted)

447	Comparing the effects of akimbo and bent-in-front arm positions on jump metrics: Validity and reliability of a modified 10/5 repeated jump test.	Celik, Huseyin ; Bulut, Suleyman	SPORTDiscus	Journal of Biomechanics	2024	10.1016/j.jbiomech.2024.111945	excluded	No comparison of arm movement in balance or postural control was conducted
448	The feasibility and efficacy of a serial reaction time task that measures motor learning of anticipatory stepping.	Olivier, Geneviève N. ; Paul, Serene S. ; Walter, Christopher S. ; Hayes, Heather A. ; Foreman, K. Bo ; Duff, Kevin ; Schaefer, Sydney Y. ; Dibble, Leland E.	SPORTDiscus	Gait & Posture	2021	10.1016/j.gaitpost.2021.04.002	excluded	No comparison of arm movement in balance or postural control was conducted
449	Control of standing balance at leaning postures with functional neuromuscular stimulation following spinal cord injury.	Audu, Musa L. ; Odle, Brooke M. ; Triolo, Ronald J.	SPORTDiscus	Medical & Biological Engineering & Computing	2018	10.1007/s11517-017-1687-x	excluded	No comparison of arm movement in balance or postural control was conducted
450	Reliability of assessing postural control during seated balancing using a physical human-robot interaction.	Ramadan, Ahmed ; Cholewicki, Jacek ; Radcliffe, Clark J. ; Jr.Popovich, John M. ; Reeves, N. Peter ; Choi, Jongeun	SPORTDiscus	Journal of Biomechanics	2017	10.1016/j.jbiomech.2017.09.036	excluded	No comparison of arm movement in balance or postural control was conducted
451	Postural regulatory strategies during quiet sitting are affected in individuals with thoracic spinal cord injury.	Milosevic, Matija ; Gagnon, Dany H. ; Gourdou, Philippe ; Nakazawa, Kimitaka	SPORTDiscus	Gait & Posture	2017	10.1016/j.gaitpost.2017.08.032	excluded	not representative of a healthy population

452	Assessment of the gait-related acceleration patterns in adults with autism spectrum disorder.	Armitano, C.N. ; Bennett, H.J. ; Haegele, J.A. ; Morrison, S.	SPORTDiscus	Gait & Posture	2020	10.1016/j.gaitpost.2019.09.002	excluded	No comparison of arm movement in balance or postural control was conducted
453	Upper Quarter Y Balance Test: reliability and performance comparison between genders in active adults	GORMAN, PAUL P. ; BUTLER, ROBERT J. ; PLISKY, PHILLIP J. ; KIESEL, KYLE B.	SPORTDiscus	Journal of Strength & Conditioning Research	2012	10.1519/JSC.0b013e3182472fdb	excluded	No comparison of arm movement in balance or postural control was conducted
454	Exploration of the y-balance test for assessment of upper quarter closed kinetic chain performance	Westrick, Richard B. ; Miller, Joseph M. ; Carow, Scott D. ; Gerber, J. Parry	SPORTDiscus	International Journal of Sports Physical Therapy	2012		excluded	No comparison of arm movement in balance or postural control was conducted
455	The influence of upper body fatigue on dynamic standing balance	Wassinger, Craig A. ; McKinney, Hayley ; Roane, Stephanie ; Davenport, Mary Jo ; Owens, Bea ; Breese, Ute ; Sokell, Geri Ann	SPORTDiscus	International Journal of Sports Physical Therapy	2014		excluded	No comparison of arm movement in balance or postural control was conducted
456	The effect of a shoulder injury prevention programme on proprioception and dynamic stability of young volleyball players; a randomized controlled trial.	Zarei, Mostafa ; Eshghi, Saeed ; Hosseinzadeh, Mahdi	SPORTDiscus	BMC Sports Science, Medicine & Rehabilitation	2021	10.1186/s13102-021-00300-5	excluded	No comparison of arm movement in balance or postural control was conducted
457	Strengthen Your Core and Watch Your Massage Improve.	Lehman, Angela	SPORTDiscus	Massage Magazine	2022		excluded	No comparison of arm movement in balance or postural control was conducted

458	Effect of Postural Control Position and Blood-Sampling Arm Position on Change in Plasma Volume from Supine Rest through a Cycling Bout.	Sullivan, William ; Fisher, Michele M.	SPORTDiscus	Journal of Exercise Physiology Online	2018		excluded	No comparison of arm movement in balance or postural control was conducted
459	Comparison of Seated Limits of Stability in Wheelchair Users and Able Body Individuals.	Garner, Tyler ; Ricard, Mark ; Yilla, Abu ; Wilson, Judy	SPORTDiscus	Archives of Physical Medicine & Rehabilitation	2024	10.1016/j.apmr.2024.02.666	excluded	No comparison of arm movement in balance or postural control was conducted
460	Arm training in standing also improves postural control in participants with chronic stroke	Waller, Sandy McCombe ; Prettyman, Michelle G.	SPORTDiscus	Gait & Posture	2012	10.1016/j.gaitpost.2012.03.025	excluded	No comparison of arm movement in balance or postural control was conducted
461	Attention Is Required to Coordinate Reaching and Postural Stability during Upper Limb Movements Generated While Standing.	Dierijck, Jill ; Kennefick, Michael ; Smirl, Jonathan ; Dalton, Brian H. ; van Donkelaar, Paul	SPORTDiscus	Journal of Motor Behavior	2020	10.1080/00222895.2019.1587351	excluded	No comparison of arm movement in balance or postural control was conducted
462	Relationship between upper limb physical performance tests and muscle strength of scapular, shoulder and spine stabilizers: A cross-sectional study.	Guirelli, Agnes Ramos ; dos Santos, Júlia Maria ; Cabral, Estêvão Mállon Gomes ; Pinto, João Pedro Camilo ; De Lima, Gabriel Alves ; Felicio, Lilian Ramiro	SPORTDiscus	Journal of Bodywork & Movement Therapies	2021	10.1016/j.jbmt.2021.05.014	excluded	No comparison of arm movement in balance or postural control was conducted
463	Upper body balance control strategy during continuous 3D postural perturbation in young adults.	Amori, V. ; Petrarca, M. ; Patané, F. ; Castelli, E. ; Cappa, P.	SPORTDiscus	Gait & Posture	2015	10.1016/j.gaitpost.2014.08.003	excluded	No comparison of arm movement (free vs restricted)

464	Effects of an Offshore Sailing Competition on Anthropometry, Muscular Performance, Subjective Wellness, and Salivary Cortisol in Professional Sailors.	Philippe, Kilian ; Paillard, Thierry ; Maurelli, Olivier ; Moody, Jeremy ; Prioux, Jacques	SPORTDiscus	International Journal of Sports Physiology & Performance	2022	10.1123/ijspp.2021-0575	excluded	No comparison of arm movement in balance or postural control was conducted
465	Upper-Extremity Functional Performance Tests: Reference Values for Overhead Athletes.	Borms, Dorien ; Cools, Ann	SPORTDiscus	International Journal of Sports Medicine	2015	10.1055/a-0573-1388	excluded	No comparison of arm movement in balance or postural control was conducted
466	Age-related changes of arm movements in dual task condition when walking on different surfaces	Hsieh, Yao-Jen ; Cho, Chiung-Yu	SPORTDiscus	Human Movement Science	2012	10.1016/j.humov.2011.01.005	excluded	No comparison of arm movement in balance or postural control was conducted
467	Fatiguing upper body aerobic exercise impairs balance	Douris, Peter C. ; Handrakis, John P. ; Gendy, Joseph ; Salama, Mina ; Kwon, Dae ; Brooks, Richard ; Salama, Nardine ; Southard, Veronica	SPORTDiscus	Journal of Strength & Conditioning Research	2011	10.1519/JSC.0b013e318215fa07	excluded	No comparison of arm movement in balance or postural control was conducted
468	Upper body accelerations during planned gait termination in young and older women.	Rum, Lorenzo ; Laudani, Luca ; Macaluso, Andrea ; Vannozzi, Giuseppe	SPORTDiscus	Journal of Biomechanics	2017	10.1016/j.jbiomech.2017.10.019	excluded	No comparison of arm movement in balance or postural control was conducted
469	Transmissibility and waveform purity of whole-body vibrations in older adults.	Lam, Freddy Man Hin ; Tang, Chak-yin ; Kwok, Timothy Chi Yui ; Pang, Marco Yiu Chung	SPORTDiscus	Clinical Biomechanics	2018	10.1016/j.clinbiomech.2017.12.007	excluded	No comparison of arm movement in balance or postural control was conducted

470	Bilateral balance and ratio of shoulder rotators in strength training practitioners and non-practitioners.	Santos, Karini ; Rodacki, André	SPORTDiscus	Sport Sciences for Health	2017	10.1007/s11332-016-0323-8	excluded	No comparison of arm movement in balance or postural control was conducted
471	Female Age-Related Differences in Biomechanics and Muscle Activity During Descents on the Outstretched Arms.	Lattimer, Lauren J. ; Lanovaz, Joel L. ; Farthing, Jonathan P. ; Madill, Stéphanie ; Soo Kim ; Robinovitch, Stephen ; Arnold, Cathy	SPORTDiscus	Journal of Aging & Physical Activity	2017	10.1123/japa.2016-0102	excluded	No comparison of arm movement in balance or postural control was conducted
472	Sensory contributions to stabilization of trunk posture in the sagittal plane.	van Dieën, Jaap H. ; van Drunen, Paul ; Happee, Riender	SPORTDiscus	Journal of Biomechanics	2018	10.1016/j.jbiomech.2017.07.016	excluded	No comparison of arm movement in balance or postural control was conducted
473	Anthropometric factors and body composition and their relationship with dynamic balance tests	Aparecida da Silva Ferreira, Brenda ; Antico Benetti, Fernanda ; Silva Luna, Natália Mariana ; Brech, Guilherme Carlos ; Sales Bocalini, Danilo ; Mesiano Maifrino, Laura Beatriz ; Magaldi, Fernanda ; D' Andrea Greve, Júlia Maria ; Castilho Alonso, Angélica	SPORTDiscus	Revista Brasileira de Medicina do Esporte	2020	10.1590/1517-869220202605190218	excluded	No comparison of arm movement in balance or postural control was conducted
474	Upper limb and trunk muscle activation during an unexpected descent on the outstretched hands in young and older women.	Lattimer, Lauren J. ; Lanovaz, Joel L. ; Farthing, Jonathan P. ; Madill, Stéphanie ; Kim, Soo ; Arnold, Cathy	SPORTDiscus	Journal of Electromyography & Kinesiology	2016	10.1016/j.jelekin.2016.08.001	excluded	No comparison of arm movement in balance or postural control was conducted

475	The effect of fixation for superior limb on upright posture in healthy adult men: evaluations by a stabilometer and a moire topography.	GYODA, Naoto ; ITOH, Yuzuru ; HAYASHI, Tomoya ; FUSHIKI, Satoshi ; YAMATSU, Takashi ; NAKAMURA, Tatsuzo	SPORTDiscus	Japanese Journal of Judo Therapy	2004		excluded	No comparison of arm movement in balance or postural control was conducted
476	Influence of Different Sitting Positions on Healthy Infants' Reaching Movements.	Moreira da Silva, Erika Shirley ; Lopes dos Santos, Gabriela ; Righetto Greco, Ana Luiza ; Tudella, Eloisa	SPORTDiscus	Journal of Motor Behavior	2017	10.1080/00222895.2016.1247034	excluded	No comparison of arm movement in balance or postural control was conducted
477	Skill level constrains the coordination of posture and upper-limb movement in a pistol-aiming task.	Ko, Ji Hynu ; Han, Dong Wook ; Newell, Karl M.	SPORTDiscus	Human Movement Science	2017	10.1016/j.humov.2017.08.017	excluded	No comparison of arm movement in balance or postural control was conducted
478	Functional Fitness Gain Varies in Older Adults Depending on Exercise Mode.	Takeshima, Nobuo ; Rogers, Nicole L. ; Rogers, Michael E.	SPORTDiscus	Medicine & Science in Sports & Exercise	2007	10.1249/mss.0b013e31814844b7	excluded	No comparison of arm movement in balance or postural control was conducted
479	The relative importance of selected physical fitness parameters in Olympic clay target shooting.	PELJHA, ZVONKO ; MICHAELIDES, MARCOS ; COLLINS, DAVE	SPORTDiscus	Journal of Human Sport & Exercise	2018	10.14198/jhse.2018.133.06	excluded	No comparison of arm movement in balance or postural control was conducted
480	Slip and Trip Perturbations During an Object Transport Task Requiring a Lateral Change in Support.	Huntley, Andrew H. ; Inkol, Keaton A. ; Vallis, Lori Ann	SPORTDiscus	Journal of Motor Behavior	2018	10.1080/00222895.2017.1363696	excluded	No comparison of arm movement in balance or postural control was conducted

481	Relationship between hand grip strength and postural static balance among undergraduates of a Nigerian university.	Ojoawo, Adesola O. ; Afolabi, Aduralere P. ; Arayombo, Babatunde E. ; Akinola, Odunayo T.	SPORTDiscus	Sports Medicine Journal / Medicina Sportiva	2018		excluded	No comparison of arm movement in balance or postural control was conducted
482	Teaching Balance Training to Improve Stability and Cognition for Children.	Shim, AndrewL. ; Norman, ShannonP. ; Kim, YoungAe	SPORTDiscus	JOPERD: The Journal of Physical Education, Recreation & Dance	2013	10.1080/07303084.2013.827076	excluded	No comparison of arm movement in balance or postural control was conducted
483	Upper-Extremity Physical-Performance Tests in College Athletes.	Taylor, Jeffrey B. ; Wright, Alexis A. ; Smoliga, James M. ; DePew, J. Tyler ; Hegedus, Eric J.	SPORTDiscus	Journal of Sport Rehabilitation	2016	10.1123/jsr.2014-0296	excluded	No comparison of arm movement in balance or postural control was conducted
484	Effects of constrained trunk movement on frontal plane gait kinematics.	Arvin, Mina ; van Dieën, Jaap H. ; Bruijn, Sjoerd M.	SPORTDiscus	Journal of Biomechanics	2016	10.1016/j.jbiomech.2016.07.015	excluded	No comparison of arm movement (free vs restricted)
485	Upper extremity sensorimotor control among collegiate football players	LAUDNER, KEVIN G.	SPORTDiscus	Journal of Strength & Conditioning Research	2012	10.1519/JSC.0b013e31822a69c8	excluded	No comparison of arm movement in balance or postural control was conducted
486	The effects of step width and arm swing on energetic cost and lateral balance during running	Arellano, Christopher J. ; Kram, Rodger	SPORTDiscus	Journal of Biomechanics	2011	10.1016/j.jbiomech.2011.01.002	excluded	No comparison of arm movement in balance or postural control was conducted
487	Effects of visual focus and gait speed on walking balance in the frontal plane.	Goodworth, Adam ; Perrone, Kathryn ; Pillsbury, Mark ; Yargeau, Michelle	SPORTDiscus	Human Movement Science	2015	10.1016/j.humov.2015.04.004	excluded	No comparison of arm movement in balance or postural control was conducted

488	Positive effects of 1-year football and strength training on mechanical muscle function and functional capacity in elderly men.	Sundstrup, Emil ; Jakobsen, Markus ; Andersen, Lars ; Andersen, Thomas ; Randers, Morten ; Helge, Jørn ; Suetta, Charlotte ; Schmidt, Jakob ; Bangsbo, Jens ; Krustrup, Peter ; Aagaard, Per ; Jakobsen, Markus Due ; Andersen, Lars Louis ; Andersen, Thomas Rostgaard ; Randers, Morten Bredsgaard ; Helge, Jørn Wulff ; Schmidt, Jakob Friis	SPORTDiscus	European Journal of Applied Physiology	2016	10.1007/s00421-016-3368-0	excluded	No comparison of arm movement in balance or postural control was conducted
489	Upper body postures effect on neuromuscular activities of the lower limb during a squat: musculoskeletal modeling.	Golfeshan, N. ; Barnamehei, H. ; Torabigoudarzi, M. ; Karimidastjerdi, M. ; Panahi, A. ; Darman, A. ; Razaghi, M. ; Kharazi, M.R. ; Barnamehei, M. ; Azad Jafarloo, S.	SPORTDiscus	Gait & Posture	2020	10.1016/j.gaitpost.2020.07.087	excluded	No comparison of arm movement in balance or postural control was conducted
490	Asymmetry of mass and motion affects the regulation of whole-body angular momentum in individuals with upper limb absence.	Kent, Jenny A. ; Major, Matthew J.	SPORTDiscus	Clinical Biomechanics	2020	10.1016/j.clinbiomech.2020.105015	excluded	not representative of a healthy population

491	Normal postural responses preceding shoulder flexion: Co-activation or asymmetric activation of transverse abdominis?	Davarian, Sanaz ; Maroufi, Nader ; Ebrahimi, Esmaeil ; Parnianpour, Mohammad ; Farahmand, Farzam	SPORTDiscus	Journal of Back & Musculoskeletal Rehabilitation	2014	10.3233/BMR-140480	excluded	No comparison of arm movement in balance or postural control was conducted
492	How does knee pain affect trunk and knee motion during badminton forehand lunges?	Huang, Ming-Tung ; Lee, Hsing-Hsan ; Lin, Cheng-Feng ; Tsai, Yi-Ju ; Liao, Jen-Chieh	SPORTDiscus	Journal of Sports Sciences	2014	10.1080/02640414.2013.848998	excluded	No comparison of arm movement in balance or postural control was conducted
493	Transversus abdominis is part of a global not local muscle synergy during arm movement.	Morris, S.L. ; Lay, B. ; Allison, G.T.	SPORTDiscus	Human Movement Science	2013	10.1016/j.humov.2012.12.011	excluded	No comparison of arm movement in balance or postural control was conducted
494	Effects of regular heel-raise training targeting the soleus muscle on dynamic balance associated with arm movement in elderly women	Fujiwara, Katsuo ; Toyama, Hiroshi ; Asai, Hitoshi ; Yaguchi, Chie ; Irei, Mariko ; Naka, Masami ; Kaida, Chizuru	SPORTDiscus	Journal of Strength & Conditioning Research	2011	10.1519/JSC.0b013e3181fb4947	excluded	No comparison of arm movement in balance or postural control was conducted
495	Tai Chi Practice Improves Senior Citizens' Balance and Arm Movement Control.	Yan, Jin H.	SPORTDiscus	Journal of Aging & Physical Activity	1998	10.1123/japa.6.3.271	excluded	No comparison of arm movement in balance or postural control was conducted
496	The Relations between Core Stability and Tennis-Related Performance Determinants.	SÖĞÜT, MUSTAFA	SPORTDiscus	Biology of Exercise	2016	10.4127/jbe.2016.0107	excluded	No comparison of arm movement in balance or postural control was conducted
497	The influence of handrail predictability on compensatory arm reactions in response to a loss of balance.	Weaver, Tyler B. ; Tokuno, Craig D.	SPORTDiscus	Gait & Posture	2013	10.1016/j.gaitpost.2012.12.003	excluded	No comparison of arm movement in balance or postural control was conducted

498	Recruitment order of the abdominal muscles varies with postural task.	Tokuno, C. D. ; Cresswell, A. G. ; Thorstensson, A. ; Carpenter, M. G.	SPORTDiscus	Scandinavian Journal of Medicine & Science in Sports	2013	10.1111/j.1600-0838.2011.01394.x	excluded	No comparison of arm movement in balance or postural control was conducted
499	Injury risk factors in junior tennis players: a prospective 2-year study.	Hjelm, N. ; Werner, S. ; Renstrom, P.	SPORTDiscus	Scandinavian Journal of Medicine & Science in Sports	2012	10.1111/j.1600-0838.2010.01129.x	excluded	No comparison of arm movement in balance or postural control was conducted
500	Trajectory Planning for the Walking Biped "Lucy".	Vermeulen, J. ; Verrelst, B. ; Vanderborght, B. ; Lefeber, D. ; Guillaume, P.	SPORTDiscus	International Journal of Robotics Research	2006	10.1177/0278364906069343	excluded	No comparison of arm movement in balance or postural control was conducted
501	Toddlers actively reorganize their whole body coordination to maintain walking stability while carrying an object.	Hsu, Wen-Hao ; Miranda, Daniel L. ; Chistolini, Trevor L. ; Goldfield, Eugene C.	SPORTDiscus	Gait & Posture	2016	10.1016/j.gaitpost.2016.08.023	excluded	No comparison of arm movement in balance or postural control was conducted
502	The effects of dual-tasking on arm muscle responses in young and older adults.	Laing, Justin M. ; Tokuno, Craig D.	SPORTDiscus	Human Movement Science	2016	10.1016/j.humov.2016.01.003	excluded	No comparison of arm movement in balance or postural control was conducted
503	Bilateral differences in upper quarter function of high school-aged baseball and softball players.	Butler, Robert J. ; Myers, Heather S. ; Black, Douglass ; Kiesel, Kyle B. ; Plisky, Phillip J. ; Moorman, Claude T. ; Queen, Robin M.	SPORTDiscus	International Journal of Sports Physical Therapy	2014		excluded	No comparison of arm movement in balance or postural control was conducted

504	Lumbar and cervical erector spinae fatigue elicit compensatory postural responses to assist in maintaining head stability during walking.	Kavanagh, Justin J. ; Morrison, Steven ; Barrett, Rod S.	SPORTDiscus	Journal of Applied Physiology	2006	10.1152/japplphysiol.00165.2006	excluded	No comparison of arm movement in balance or postural control was conducted
505	Manual movement coordination adapted to spinal cord injury and low back pain	Kim, K. Han ; Martin, Bernard J.	SPORTDiscus	International Journal of Industrial Ergonomics	2013	10.1016/j.ergon.2012.10.002	excluded	No comparison of arm movement in balance or postural control was conducted
506	Effectiveness of different visual biofeedback signals for human balance improvement.	Halická, Zuzana ; Lobotková, Jana ; Bučková, Kristína ; Hlavačka, František	SPORTDiscus	Gait & Posture	2014	10.1016/j.gaitpost.2013.08.005	excluded	No comparison of arm movement in balance or postural control was conducted
507	Timing of muscle activity during reaching while standing: systematic changes with target distance	Tyler, Amy E. ; Karst, Gregory M.	SPORTDiscus	Gait & Posture	2004	10.1016/j.gaitpost.2003.07.001	excluded	No comparison of arm movement in balance or postural control was conducted
508	Identifying intrinsic and reflexive contributions to low-back stabilization.	van Drunen, P. ; Maaswinkel, E. ; van der Helm, F. C. T. ; van Dieën, J. H. ; Happee, R.	SPORTDiscus	Journal of Biomechanics	2013	10.1016/j.jbiomech.2013.03.007	excluded	No comparison of arm movement in balance or postural control was conducted
509	Arm yourself	Crandell, Jason	SPORTDiscus	Yoga Journal	2017		excluded	No comparison of arm movement in balance or postural control was conducted

510	Movement patterns and muscular demands during posterior transfers toward an elevated surface in individuals with spinal cord injury.	Gagnon, D. ; Nadeau, S. ; Gravel, D. ; Noreau, L. ; Larivière, C. ; McFadyen, B.	SPORTDiscus	Spinal Cord	2005	10.1038/sj.sc.3101660	excluded	No comparison of arm movement in balance or postural control was conducted
511	Balance control during walking in the older adult: Research and its implications.	Woollacott, Marjorie H. ; Tang, Pei-Fang	SPORTDiscus	Physical Therapy	1997	10.1093/ptj/77.6.646	excluded	No comparison of arm movement in balance or postural control was conducted
512	how fit are you?	Everett, Jenny	SPORTDiscus	Runner's World (Australia & New Zealand Edition)	2010		excluded	No comparison of arm movement in balance or postural control was conducted
513	The Clinic PHOTO CRITIQUES.	von Dietze, Susanne	SPORTDiscus	Dressage Today	2016		excluded	No comparison of arm movement in balance or postural control was conducted
514	Lesson plan for a balance class.	Bovre, Susan	SPORTDiscus	Functional U	2005		excluded	No comparison of arm movement in balance or postural control was conducted
515	Evidence of nonlocal muscle fatigue in male youth.	Ben Othman, Aymen ; Chaouachi, Anis ; Hammami, Raouf ; Chaouachi, Mehdi M. ; Kasmi, Sofien ; Behm, David G.	SPORTDiscus	Applied Physiology, Nutrition & Metabolism	2017	10.1139/apnm-2016-0400	excluded	No comparison of arm movement in balance or postural control was conducted

516	Reduction of neuromuscular redundancy for postural force generation using an intrinsic stability criterion	Bunderson, Nathan E. ; Burkholder, Thomas J. ; Ting, Lena H.	SPORTDiscus	Journal of Biomechanics	2008	10.1016/j.jbiomech.2008.02.004	excluded	No comparison of arm movement in balance or postural control was conducted
517	Anticipatory postural control in children.	Riach, C.L. ; Hayes, K.C.	SPORTDiscus	Journal of Motor Behavior	1990		excluded	No comparison of arm movement in balance or postural control was conducted
518	Relationship between Upper Quarter Y Balance Test performance and throwing proficiency in adolescent Olympic handball players.	Bauer, Julian ; Schedler, Simon ; Fischer, Stephan ; Muehlbauer, Thomas	SPORTDiscus	BMC Sports Science, Medicine & Rehabilitation	2020	10.1186/s13102-020-00199-4	excluded	No comparison of arm movement in balance or postural control was conducted
519	Physical characteristics of experienced and junior open-wheel car drivers.	Raschner, Christian ; Platzer, Hans-Peter ; Patterson, Carson	SPORTDiscus	Journal of Sports Sciences	2013	10.1080/02640414.2012.720703	excluded	No comparison of arm movement in balance or postural control was conducted
520	Effects of medio-lateral postural perturbation induced by voluntary arm raising on the biomechanical organization of rapid step initiation.	Yiou E ; Do MC ; Yiou, Eric ; Do, Manh-Cuong	SPORTDiscus	Motor Control	2011		excluded	No comparison of arm movement in balance or postural control was conducted
521	Reliability and validity of physical fitness field tests for adults aged 55 to 70 years.	Ritchie, C. ; Trost, S.G. ; Brown, W. ; Armit, C.	SPORTDiscus	Journal of Science & Medicine in Sport	2005		excluded	No comparison of arm movement in balance or postural control was conducted

522	Quantitative assessment of trunk movements in functional reaching in children and adolescents with dyskinetic cerebral palsy.	Van Wonterghem, Ellen ; Vanmechelen, Inti ; Haberfehlner, Helga ; Decrock, Bieke ; Monbaliu, Elegast	SPORTDiscus	Clinical Biomechanics	2023	10.1016/j.clinbiomech.2023.105876	excluded	No comparison of arm movement in balance or postural control was conducted
523	Effects of cognitive task execution on stable and unstable surface balance.	Quesada, Peter M. ; Geiger, Jeffery T.	SPORTDiscus	Cogent Engineering	2017	10.1080/23311916.2017.1311440	excluded	No comparison of arm movement in balance or postural control was conducted
524	Does the “eyes lead the hand” principle apply to reach-to-grasp movements evoked by unexpected balance perturbations?	King, Emily C. ; Lee, Tracy A. ; McKay, Sandra M. ; Scovil, Carol Y. ; Peters, Amy L. ; Pratt, Jay ; Maki, Brian E.	SPORTDiscus	Human Movement Science	2011	10.1016/j.humov.2010.07.005	excluded	No comparison of arm movement (free vs restricted)
525	Sex Differences in Dynamic Closed Kinetic Chain Upper Quarter Function in Collegiate Swimmers.	Butler, Robert ; Arms, Jennifer ; Reiman, Michael ; Plisky, Phillip ; Kiesel, Kyle ; Taylor, Dean ; Queen, Robin	SPORTDiscus	Journal of Athletic Training (Allen Press)	2014	10.4085/1062-6050-49.3.17	excluded	No comparison of arm movement in balance or postural control was conducted
526	Postural Adjustments in Catching: On the Interplay Between Segment Stabilization and Equilibrium Control.	Tijtgat, Pieter ; Vanrenterghem, Jos ; Bennett, Simon J. ; De Clercq, Dirk ; Savelsbergh, Geert J. P. ; Lenoir, Matthieu	SPORTDiscus	Motor Control	2013	10.1123/mcj.17.1.48	excluded	No comparison of arm movement in balance or postural control was conducted
527	Physical fitness differences between prepubescent boys and girls	Marta, Carlos C. ; Marinho, Daniel A. ; Barbosa, Tiago M. ; Izquierdo, Mikel ; Marques, Mário C.	SPORTDiscus	Journal of Strength & Conditioning Research	2012	10.1519/JSC.0b013e31825bb4aa	excluded	No comparison of arm movement in balance or postural control was conducted

528	Analysis of vertical ground reaction force variables during a Sit to Stand task in participants recovering from a hip fracture	Houck, Jeff ; Kneiss, Janet ; Bukata, Susan V. ; Puzas, J. Edward	SPORTDiscus	Clinical Biomechanics	2011	10.1016/j.clinbiomech.2010.12.004	excluded	No comparison of arm movement in balance or postural control was conducted
529	Frontal plane standing balance with an ambulation aid:Upper limb biomechanics	Tung, James Y. ; Gage, William H. ; Zabjek, Karl F. ; Maki, Brian E. ; McIlroy, William E.	SPORTDiscus	Journal of Biomechanics	2011	10.1016/j.jbiomech.2011.03.015	excluded	No comparison of arm movement in balance or postural control was conducted
530	The role of strategy selection, limb force capacity and limb positioning in successful trip recovery	Roos, Paulien E. ; McGuigan, M. Polly ; Trewartha, Grant	SPORTDiscus	Clinical Biomechanics	2010	10.1016/j.clinbiomech.2010.06.016	excluded	No comparison of arm movement (free vs restricted)
531	Difference in the metabolic cost of postural actions during iso- and antidirectional coupled oscillations of the upper limbs in the horizontal plane.	Esposti, Roberto ; Esposito, Fabio ; Cé, Emiliano ; Baldissera, Fausto ; Cé, Emiliano	SPORTDiscus	European Journal of Applied Physiology	2010	10.1007/s00421-009-1193-4	excluded	No comparison of arm movement in balance or postural control was conducted
532	Does symmetrical upper limb task involve symmetrical postural adjustments?	Mezaour, M. ; Yiou, E. ; Le Bozec, S.	SPORTDiscus	Gait & Posture	2009	10.1016/j.gaitpost.2009.05.007	excluded	No comparison of arm movement (free vs restricted)
533	The Influence of Age and Physical Activity on Upper Limb Proprioceptive Ability.	Adamo, Diane E. ; Alexander, Neil B. ; Susan H. Brown	SPORTDiscus	Journal of Aging & Physical Activity	2009	10.1123/japa.17.3.272	excluded	No comparison of arm movement in balance or postural control was conducted

534	Anticipatory Postural Adjustments and Focal Performance During Bilateral Forward-Reach Task Under Different Stance Conditions.	Yiou, Eric ; Mezaour, Malha ; Le Bozec, Serge	SPORTDiscus	Motor Control	2009	10.1123/mcj.13.2.142	excluded	No comparison of arm movement in balance or postural control was conducted
535	mission possible.	Crandell, Jason	SPORTDiscus	Yoga Journal	2014		excluded	No comparison of arm movement in balance or postural control was conducted
536	Balance is everything	FINLAYSON, TRENT	SPORTDiscus	WaterSki	2013		excluded	No comparison of arm movement in balance or postural control was conducted
537	A biomechanical comparison of powered robotic exoskeleton gait with normal and slow walking: An investigation with able-bodied individuals.	Hayes, Stephen Clive ; White, Matthew ; White, Hollie Samantha Forbes ; Vanicek, Natalie	SPORTDiscus	Clinical Biomechanics	2020	10.1016/j.clinbiomech.2020.105133	excluded	No comparison of arm movement in balance or postural control was conducted
538	How do step width and arm swing affect energetic cost and lateral balance during running?	Arellano, Christopher J. ; Kram, Rodger	SPORTDiscus	Conference Proceedings of the Annual Meeting of the American Society of Biomechanics	2010		excluded	No comparison of arm movement in balance or postural control was conducted

539	Activation of transversus abdominis varies with postural demand in standing	Crommert, M. Eriksson ; Ekblom, M.M. ; Thorstensson, A.	SPORTDiscus	Gait & Posture	2011	10.1016/j.gaitpost.2010.12.028	excluded	No comparison of arm movement in balance or postural control was conducted
540	Posture Control and Complex Arm Coordination: Analysis of Multijoint Coordinative Movements and Stability of Stance.	Forner-Cordero, A. ; Levin, O. ; Li, Y. ; Swinnen, S. P.	SPORTDiscus	Journal of Motor Behavior	2007	10.3200/JMBR.39.3.215-226	excluded	No comparison of arm movement (free vs restricted)
541	Ready, Set, Hike.	McDowell, Dimity	SPORTDiscus	Joe Weider's Muscle & Fitness Hers	2004		excluded	No comparison of arm movement in balance or postural control was conducted
542	Effectiveness of Traditional and Sling Exercise Strength Training in Novice Women.	Dannelly, B D ; Otey, S C ; Croy, T ; Harrison, B ; Rynders, C ; Hertel, J ; Weltman, A	SPORTDiscus	Journal of Strength & Conditioning Research	2011	10.1097/01.JSC.0000395709.90084.44	excluded	No comparison of arm movement in balance or postural control was conducted
543	Tele Falling Leaf.	McGee, J. Scott	SPORTDiscus	Skitrax	2014		excluded	No comparison of arm movement in balance or postural control was conducted
544	Triple A - Abdominals, Arms, Awareness.	Meyers, Lori	SPORTDiscus	Skitrax	2014		excluded	No comparison of arm movement in balance or postural control was conducted
545	**Does outstretching the arms improve postural stability?**	**Mitesh Patel, David Buckwell, Malcolm Hawken, Adolfo M. Bronstein**		**Neuroscience Letters**	**2014**	**https://doi.org/10.1016/j.neulet.2014.07.010**	**included**	
546	A Matter of Balance	Woods, Tiger ; McDaniel, Pete ; Soltau, Mark	SPORTDiscus	Golf Digest	2008		excluded	No comparison of arm movement in balance or postural control was conducted

547	Armed and Dangerous	Libby, Brian	SPORTDiscus	Men's Fitness	2005		excluded	No comparison of arm movement in balance or postural control was conducted
548	A study of infant posture: what is the contribution of arm movements to control of the center of mass during the perturbation of reaching?	McGovern-Zlotek, C. M.	SPORTDiscus		1998		excluded	No comparison of arm movement in balance or postural control was conducted
549	Balance adjustments in perturbed human walking: neuromuscular control mechanisms and effects of aging.	Tang, P.	SPORTDiscus		1997		excluded	No comparison of arm movement in balance or postural control was conducted
550	Acute Knee Crutch Use Provokes Changes to Postural Strategy.	Maron, Christian ; Jendre, Aron ; Goble, Daniel ; Marks, Charles ; Haworth, Joshua	SPORTDiscus	Perceptual & Motor Skills	2024	10.1177/00315125241246390	excluded	No comparison of arm movement in balance or postural control was conducted
551	Proprioceptive Reweighting and Postural Control are Impaired Among Elite Athletes Following Anterior Cruciate Ligament Reconstruction.	Attalin, Benoit ; Sagnard, Telma ; Laboute, Eric ; Forestier, Nicolas ; Rémy-Néris, Olivier ; Picot, Brice	SPORTDiscus	International Journal of Sports Physical Therapy	2024	10.26603/001c.124802	excluded	No comparison of arm movement in balance or postural control was conducted
552	Deficits in recovery of postural stability after stepping are limb- and phase-specific in children with unilateral cerebral palsy.	Campbell, Katelyn S. ; Whitten, Sydni V.W. ; Newell, Karl M. ; Li, Li ; Singh, Tarkeshwar ; Khan, Owais A. ; Modlesky, Christopher M.	SPORTDiscus	Gait & Posture	2024	10.1016/j.gaitpost.2024.09.013	excluded	No comparison of arm movement in balance or postural control was conducted

553	Intersession reliability of center of pressure measurement during bipedal standing with different count-back orders.	Saberi, Shirin ; Mosharaf, Mahshid ; Yeowell, Gillian ; Sadeghi-Demneh, Ebrahim	SPORTDiscus	Journal of Bodywork & Movement Therapies	2024	10.1016/j.jbmt.2024.05.026	excluded	No comparison of arm movement in balance or postural control was conducted
554	**Effect of arm motion on postural stability when recovering from a slip perturbation**	**Gholizadeh, H; Hill, A; Nantel, J**	**Web of Science**	**JOURNAL OF BIOMECHANICS**	**2019**	**10.1016/j.jbiomech.2019.07.013**	**included**	
555	**Increased Arm Swing and Rocky Surfaces Reduces Postural Control in Healthy Young Adults**	**Mezher, C; Siragy, T; Nantel, J**	**Web of Science**	**FRONTIERS IN BIOENGINEERING AND BIOTECHNOLOGY**	**2021**	**10.3389/fbioe.2021.645581**	**included**	
556	Effect of upper extremity constraints on functional and dynamic postural control in children with hemiplegic cerebral palsy	Roostaei, M; Raji, P; Kalantari, KK; Faghihzadeh, E; Fragala-Pinkham, M	Web of Science	DEVELOPMENTAL NEUROREHABILITATION	2022	10.1080/17518423.2021.2020351	excluded	not representative of a healthy population
557	Compensatory mechanisms of balance to the scaling of arm-swing frequency	Ko, JH; Wang, Z; Challis, JH; Newell, KM	Web of Science	JOURNAL OF BIOMECHANICS	2015	10.1016/j.jbiomech.2015.09.008	excluded	No comparison of arm movement in balance or postural control was conducted
558	**Effect of Arm Movement and Task Difficulty on Balance Performance in Children, Adolescents, and Young Adults**	**Muehlbauer, T; Hill, MW; Heise, J; Abel, L; Schumann, I; Brueckner, D; Schedler, S**	**Web of Science**	**FRONTIERS IN HUMAN NEUROSCIENCE**	**2022**	**10.3389/fnhum.2022.854823**	**included**	

559	Effect of Cardiovascular Fatigue on Postural Stability	Bateni, H; Leno, G; Manjarres, R; Ouellette, B; Wolber, M	Web of Science	INTERNATIONAL JOURNAL OF ATHLETIC THERAPY & TRAINING	2013	10.1123/ijatt.18.5.38	excluded	No comparison of arm movement in balance or postural control was conducted
560	**Effect of arm movement on balance performance in children: role of expertise in gymnastics**	**Muehlbauer, T; Heise, J; Hill, MW**	**Web of Science**	**BMC RESEARCH NOTES**	**2022**	**10.1186/s13104-022-06182-1**	**included**	
561	**Dynamic Postural Control in Children: Do the Arms Lend the Legs a Helping Hand?**	**Hill, MW; Wdowski, MM; Pennell, A; Stodden, DF; Duncan, MJ**	**Web of Science**	**FRONTIERS IN PHYSIOLOGY**	**2019**	**10.3389/fphys.2018.01932**	**included**	
562	Upper limb-related factors in determining postural control	Kodak, MI; Ozudogru, A; Ozsoy, I	Web of Science	KINESIOLOGIA SLOVENICA	2021		excluded	No comparison of arm movement in balance or postural control was conducted
563	**Effect of arm movement and task difficulty level on balance performance in healthy children: are there sex differences?**	**Muehlbauer, T; Hill, MW; Schedler, S**	**Web of Science**	**BMC RESEARCH NOTES**	**2022**	**10.1186/s13104-022-06195-w**	**included**	
564	**Effects of arm movement strategies on emotional state and balance control during height-induced postural threat in young adults**	**Hill, MW; Russel, K; Wdowski, M; Lord, SR; Muehlbauer, T; Ellmers, T**	**Web of Science**	**GAIT & POSTURE**	**2023**	**10.1016/j.gaitpost.2023.04.020**	**included**	

565	Validity and reliability of upper extremity star excursion balance test in adolescent swimmers	Xu, HR; Zhang, YH; Mao, Y; Ngo, TL; Zhang, Q; He, G; Feng, ZM; Sun, WJ; Wang, XQ	Web of Science	JOURNAL OF EXERCISE SCIENCE & FITNESS	2023	10.1016/j.jesf.2023.02.003	excluded	No comparison of arm movement in balance or postural control was conducted
566	Hand reach star excursion balance test: An alternative test for dynamic postural control and functional mobility	Eriksrud, O; Federolf, P; Anderson, P; Cabri, J	Web of Science	PLOS ONE	2018	10.1371/journal.pone.0196813	excluded	No comparison of arm movement in balance or postural control was conducted
567	Improving static balance ability with trainings supported by somatosensory-based feedback system	Wang, W; Wang, WJ; Shadiev, R	Web of Science	SMART LEARNING ENVIRONMENTS	2022	10.1186/s40561-022-00216-8	excluded	No comparison of arm movement in balance or postural control was conducted
568	Upper extremity function: What's posture got to do with it?	Harbourne, R; Kamm, K	Web of Science	JOURNAL OF HAND THERAPY	2015	10.1016/j.jht.2015.01.008	excluded	No comparison of arm movement in balance or postural control was conducted
569	Influence of protocol variables on outcomes of the star excursion balance test group (SEBT, mSEBT, YBT-LQ) in healthy individuals: a systematic review	Zajac, B; Olszewski, M; Mika, A	Web of Science	FRONTIERS IN PHYSIOLOGY	2024	10.3389/fphys.2024.1415887	excluded	No comparison of arm movement in balance or postural control was conducted
570	Upper-Extremity Physical-Performance Tests in College Athletes	Taylor, JB; Wright, AA; Smoliga, JM; Depew, JT; Hegedus, EJ	Web of Science	JOURNAL OF SPORT REHABILITATION	2016	10.1123/jsr.2014-0296	excluded	No comparison of arm movement in balance or postural control was conducted

571	Does Lymphedema Affect the Postural Stability in Women After Breast Cancer?	Basar, S; Bakar, Y; Keser, I; Kaba, H; Güzel, NA; Özdemir, ÖÇ; Düzgün, I	Web of Science	TOPICS IN GERIATRIC REHABILITATION	2012	10.1097/TGR.0b013e318270c89b	excluded	No comparison of arm movement in balance or postural control was conducted
572	Influence of typical handball characteristics on upper body posture and postural control in male handball players	Ohlendorf, D; Salzer, S; Haensel, R; Rey, J; Maltry, L; Holzgreve, F; Lampe, J; Wanke, EM; Groneberg, DA	Web of Science	BMC SPORTS SCIENCE MEDICINE AND REHABILITATION	2020	10.1186/s13102-020-0156-2	excluded	No comparison of arm movement in balance or postural control was conducted
573	Impact of Diaphragm-Strengthening Core Training on Postural Stability in High-Intensity Squats	Seo, H; Jeong, G; Chun, B	Web of Science	LIFE-BASEL	2024	10.3390/life14121612	excluded	No comparison of arm movement in balance or postural control was conducted
574	Effect of a Short-Term Combined Balance and Multidirectional Plyometric Training on Postural Balance and Explosive Performance in U-13 Male and Female Soccer Athletes	Ioannou, G; Kanioris, E; Nikolaidou, ME	Web of Science	APPLIED SCIENCES-BASEL	2024	10.3390/app14104141	excluded	No comparison of arm movement in balance or postural control was conducted
575	Relationship between postural alignment in sitting by photogrammetry and seated postural control in post-stroke subjects	Iyengar, YR; Vijayakumar, K; Abraham, JM; Misri, ZK; Suresh, BV; Unnikrishnan, B	Web of Science	NEUROREHABILITATION	2014	10.3233/NRE-141118	excluded	No comparison of arm movement in balance or postural control was conducted

576	Differences in postural control and movement performance during goal directed reaching in children with developmental coordination disorder	Johnston, LM; Burns, YR; Brauer, SG; Richardson, CA	Web of Science	HUMAN MOVEMENT SCIENCE	2002	10.1016/S0167-9457(02)00153-7	excluded	No comparison of arm movement in balance or postural control was conducted
577	The Influence of Treadmill on Postural Control	Tong, JG; Zhang, JC; Dong, EZ; Liu, C; Du, SZ	Web of Science	IEEE ACCESS	2020	10.1109/ACCESS.2020.3019442	excluded	No comparison of arm movement in balance or postural control was conducted
578	Accuracy of KinectOne to quantify kinematics of the upper body	Kuster, RP; Heinlein, B; Bauer, CM; Graf, ES	Web of Science	GAIT & POSTURE	2016	10.1016/j.gaitpost.2016.04.004	excluded	No comparison of arm movement in balance or postural control was conducted
579	Effects of Nontraditional Division III Lacrosse Participation on Movement Pattern Quality and Dynamic Postural Control	Rosenborough, C; Collins, SM; Smith, E; Bowman, TG	Web of Science	INTERNATIONAL JOURNAL OF SPORTS PHYSICAL THERAPY	2024	10.26603/001c.115423	excluded	No comparison of arm movement in balance or postural control was conducted
580	**Changes in joint kinematics and dynamic postural stability with free and restricted arm movements in children**	**Wdowski, MM; Duncan, MJ; Pennell, A; Hill, MW**	**Web of Science**	**GAIT & POSTURE**	**2021**	**10.1016/j.gaitpost.2021.05.010**	**included**	
581	The Relationship between Trunk Control and Upper Extremity Function in Children with Obstetric Brachial Plexus Palsy	Çelik, G; Delioglu, K; Firat, T	Web of Science	DEVELOPMENTAL NEUROREHABILITATION	2021	10.1080/17518423.2020.1800856	excluded	not representative of a healthy population

582	Examining Fundamental Movement Competency and Closed-Chain Upper-Extremity Dynamic Balance in Swimmers	Bullock, GS; Brookreson, N; Knab, AM; Butler, RJ	Web of Science	JOURNAL OF STRENGTH AND CONDITIONING RESEARCH	2017	10.1519/JSC.0000000000001627	excluded	No comparison of arm movement in balance or postural control was conducted
583	Postural stability, clicker reaction time and bow draw force predict performance in elite recurve archery	Spratford, W; Campbell, R	Web of Science	EUROPEAN JOURNAL OF SPORT SCIENCE	2017	10.1080/17461391.2017.1285963	excluded	No comparison of arm movement in balance or postural control was conducted
584	Effect of exercise performance by elderly women on balance ability and muscle function	Lee, HC; Lee, ML; Kim, SR	Web of Science	JOURNAL OF PHYSICAL THERAPY SCIENCE	2015	10.1589/jpts.27.989	excluded	No comparison of arm movement in balance or postural control was conducted
585	Associations between upper quarter Y-balance test performance and sport-related injuries in adolescent handball players	Bauer, J; Panzer, S; Gruber, M; Muehlbauer, T	Web of Science	FRONTIERS IN SPORTS AND ACTIVE LIVING	2023	10.3389/fspor.2023.1076373	excluded	No comparison of arm movement in balance or postural control was conducted
586	Does Orthotic Use Affect Upper Extremity Support During Upright Play in Infants With Down Syndrome?	Looper, J; Ulrich, D	Web of Science	PEDIATRIC PHYSICAL THERAPY	2011	10.1097/PEP.0b013e318208cdea	excluded	No comparison of arm movement in balance or postural control was conducted
587	Upper body accelerations during planned gait termination in young and older women	Rum, L; Laudani, L; Macaluso, A; Vannozzi, G	Web of Science	JOURNAL OF BIOMECHANICS	2017	10.1016/j.jbiomech.2017.10.019	excluded	No comparison of arm movement in balance or postural control was conducted

588	Effects of six weeks of stable versus unstable multi-dimensional surfaces balance training on passing skills and balance performance in young male basketball players	Fisek, T; Agopyan, A	Web of Science	JOURNAL OF MENS HEALTH	2021	10.31083/jomh.2021.073	excluded	No comparison of arm movement in balance or postural control was conducted
589	Interaction of the reactive moments and centre of mass displacement for postural control during voluntary arm movements	ENG, JJ; WINTER, DA; MACKINNON, CD; PATLA, AE	Web of Science	NEUROSCIENCE RESEARCH COMMUNICATIONS	1992		excluded	No comparison of arm movement (free vs restricted)
590	Seated reach distance and trunk excursion accurately reflect dynamic postural control in individuals with motor-incomplete spinal cord injury	Field-Fote, EC; Ray, SS	Web of Science	SPINAL CORD	2010	10.1038/sc.2010.11	excluded	not representative of a healthy population
591	Effects Of Seat-Surface Inclination On Postural Stability And Function Of The Upper Extremities Of Children With Cerebral Palsy	MCCLENAGHAN, BA; THOMBS, L; MILNER, M	Web of Science	DEVELOPMENTAL MEDICINE AND CHILD NEUROLOGY	1992		excluded	not representative of a healthy population
592	**The Contribution of Upper Body Movements to Dynamic Balance Regulation during Challenged Locomotion**	**Boström, KJ; Dirksen, T; Zentgraf, K; Wagner, H**	**Web of Science**	**FRONTIERS IN HUMAN NEUROSCIENCE**	**2018**	**10.3389/fnhum.2018.00008**	**included**	

593	Anticipatory control of center of mass and joint stability during voluntary arm movement from a standing posture: interplay between active and passive control	Patla, AE; Ishac, MG; Winter, DA	Web of Science	EXPERIMENTAL BRAIN RESEARCH	2002	10.1007/s00221-001-0968-6	excluded	No comparison of arm movement (free vs restricted)
594	Effect of Adaptive Seating Systems on Postural Control and Activity Performance: A Systematic Review	Acharya, BD; Karki, A; Prasertsukdee, S; Reed, D; Rawal, L; Baniya, PL; Boyd, RN	Web of Science	PEDIATRIC PHYSICAL THERAPY	2023	10.1097/PEP.0000000000001042	excluded	No comparison of arm movement in balance or postural control was conducted
595	Reliability and Validity of the Star Excursion Balance Test for Evaluating Dynamic Balance of Upper Extremities	Yang, QH; Zhang, YH; Du, SH; Wang, YC; Xu, HR; Chen, JW; Mao, Y; Wang, XQ	Web of Science	SPORTS HEALTH-A MULTIDISCIPLINARY APPROACH	2024	10.1177/19417381231221716	excluded	No comparison of arm movement in balance or postural control was conducted
596	Threshold position control of arm movement with anticipatory increase in grip force	Pilon, JF; De Serres, SJ; Feldman, AG	Web of Science	EXPERIMENTAL BRAIN RESEARCH	2007	10.1007/s00221-007-0901-8	excluded	grip context
597	Anticipatory postural adjustments to arm movement reveal complex control of paraspinal muscles in the thorax	Lee, LJ; Coppieters, MW; Hodges, PW	Web of Science	JOURNAL OF ELECTROMYOGRAPHY AND KINESIOLOGY	2009	10.1016/j.jelekin.2007.06.015	excluded	No comparison of arm movement in balance or postural control was conducted

598	Does Nordic walking improves the postural control and gait parameters of women between the age 65 and 74: a randomized trial	Kocur, P; Wiernicka, M; Wilski, M; Kaminska, E; Furmaniuk, L; Maslowska, MF; Lewandowski, J	Web of Science	JOURNAL OF PHYSICAL THERAPY SCIENCE	2015	10.1589/jpts.27.3733	excluded	No comparison of arm movement in balance or postural control was conducted
599	**Effects of free versus restricted arm movements on postural control in normal and modified sensory conditions inyoung and older adults**	**Johnson, E; Ellmers, TJ; Muehlbauer, T; Lord, SR; Hill, MW**	**Web of Science**	**EXPERIMENTAL GERONTOLOGY**	**2023**	**10.1016/j.exger.2023.112338**	**included**	
600	Analysis of Reach-to-Grasp by School-Aged Children with Down Syndrome Elucidates Limitations in Upper Extremity Motor Control	Valvano, J; Hogy, S; Worster, K; Ma, J; Denniston, N; Winders, P; Rapport, MJ; Pan, ZX; Carollo, JJ	Web of Science	PHYSICAL & OCCUPATIONAL THERAPY IN PEDIATRICS	2017	10.1080/01942638.2016.1261979	excluded	not representative of a healthy population
601	Comparative analysis of postural stability in elite and novice recurve archers	Ganjave, P; Dabholkar, A	Web of Science	MEDICAL SCIENCE	2020		excluded	No comparison of arm movement in balance or postural control was conducted

602	Long-Lasting Event-Related Beta Synchronizations of Electroencephalographic Activity in Response to Support-Surface Perturbations During Upright Stance: A Pilot Study Associating Beta Rebound and Active Monitoring in the Intermittent Postural Control	Nakamura, A; Suzuki, Y; Milosevic, M; Nomura, T	Web of Science	FRONTIERS IN SYSTEMS NEUROSCIENCE	2021	10.3389/fnsys.2021.660434	excluded	No comparison of arm movement in balance or postural control was conducted
603	Shoulder Strength and Upper Body Field Performance Tests in Young Female Handball and Volleyball Athletes: Are There Differences Between Sports?	Saccol, MF; Zanca, GG; Machado, RO; Teixeira, LP; Löbell, R; Cools, A; Mota, CB	Web of Science	JOURNAL OF SPORT REHABILITATION	2022	10.1123/jsr.2021-0221	excluded	No comparison of arm movement in balance or postural control was conducted
604	Effects of concurrent physical and cognitive demands on arm movement kinematics in a repetitive upper-extremity precision task	Srinivasan, D; Mathiassen, SE; Samani, A; Madeleine, P	Web of Science	HUMAN MOVEMENT SCIENCE	2015	10.1016/j.humov.2015.05.001	excluded	No balance or postural control was conducted
605	Intelligent Locomotion Planning With Enhanced Postural Stability for Lower-Limb Exoskeletons	Mehr, JK; Sharifi, M; Mushahwar, VK; Tavakoli, M	Web of Science	IEEE ROBOTICS AND AUTOMATION LETTERS	2021	10.1109/LRA.2021.3098915	excluded	No comparison of arm movement in balance or postural control was conducted

606	Adaptations of the Upper Body to Plyometric Training in Cricket Players of Different Age Groups	Singla, D; Hussain, ME	Web of Science	JOURNAL OF SPORT REHABILITATION	2020	10.1123/jsr.2018-0469	excluded	No comparison of arm movement in balance or postural control was conducted
607	Postural control and contingent negative variation during transient floor translation while standing with the ankle fixed	Lytnev, V; Fujiwara, K; Kiyota, N; Irei, M; Toyama, H; Yaguchi, C	Web of Science	JOURNAL OF PHYSIOLOGICAL ANTHROPOLOGY	2016	10.1186/s40101-016-0104-8	excluded	No comparison of arm movement (free vs restricted)
608	Effects of Thrower's Ten exercises on upper extremity performance A randomized controlled study	Gokalp, O; Kirmizigil, B	Web of Science	MEDICINE	2020	10.1097/MD.0000000000022837	excluded	No comparison of arm movement in balance or postural control was conducted
609	Stability, performance and upper and lower extremities range of motion in elite beach handball athletes: A cross sectional study	Barbosa, GM; Saccol, MF; Pinheiro, SM; Costa, IDS; Camargo, PR; Silva, RS	Web of Science	JOURNAL OF BODYWORK AND MOVEMENT THERAPIES	2023	10.1016/j.jbmt.2023.04.023	excluded	No comparison of arm movement in balance or postural control was conducted
610	Aging and balance control in response to external perturbations: role of anticipatory and compensatory postural mechanisms	Kanekar, N; Aruin, AS	Web of Science	AGE	2014	10.1007/s11357-014-9621-8	excluded	No comparison of arm movement in balance or postural control was conducted

611	Effect of time pressure on attentional shift and anticipatory postural control during unilateral shoulder abduction reactions in an oddball-like paradigm	Anan, K; Fujiwara, K; Yaguchi, C; Kiyota, N	Web of Science	JOURNAL OF PHYSIOLOGICAL ANTHROPOLOGY	2014	10.1186/1880-6805-33-17	excluded	No comparison of arm movement in balance or postural control was conducted
612	Postural stability effects of random vibration at the feet of construction workers in simulated elevation	Simeonov, P; Hsiao, H; Powers, J; Ammons, D; Kau, T; Amendola, A	Web of Science	APPLIED ERGONOMICS	2011	10.1016/j.apergo.2010.10.002	excluded	No comparison of arm movement in balance or postural control was conducted
613	Age-related asymmetry in anticipatory postural movements during unilateral arm movement and imagery	Wider, C; Mitra, S; Boulton, H; Andrews, M	Web of Science	EXPERIMENTAL BRAIN RESEARCH	2022	10.1007/s00221-022-06416-5	excluded	No comparison of arm movement in balance or postural control was conducted
614	Comparing the Impact of Upper Body Control and Core Muscle Stabilization Training on Landing Biomechanics in Individuals with Functional Ankle Instability: A Randomized Controlled Trial	Nekar, DM; Lee, DY; Hong, JH; Kim, JS; Kim, SG; Nam, YG; Yu, JH	Web of Science	HEALTHCARE	2024	10.3390/healthcare12010070	excluded	not representative of a healthy population
615	Backward Walking Styles and Impact on Spatiotemporal Gait Characteristics	Luecha, T; Takesue, S; Yeoh, WL; Loh, PY; Muraki, S	Web of Science	HEALTHCARE	2022	10.3390/healthcare10122487	excluded	No comparison of arm movement in balance or postural control was conducted

616	Neuromechanical response of the upper body to unexpected perturbations during gait initiation in young and older adults	Rum, L; Vannozzi, G; Macaluso, A; Laudani, L	Web of Science	AGING CLINICAL AND EXPERIMENTAL RESEARCH	2021	10.1007/s40520-020-01592-2	excluded	No comparison of arm movement in balance or postural control was conducted
617	Proprioception and dynamic balance performance in wrestlers: Freestyle vs. Greco-Romana	Tabasi, SR; Norasteh, AA; Mirzaei, B; Zarei, H	Web of Science	SCIENCE & SPORTS	2022	10.1016/j.scispo.2021.06.011	excluded	No comparison of arm movement in balance or postural control was conducted
618	Biarticular muscles are most responsive to upper-body pitch perturbations in human standing	Schumacher, C; Berry, A; Lemus, D; Rode, C; Seyfarth, A; Vallery, H	Web of Science	SCIENTIFIC REPORTS	2019	10.1038/s41598-019-50995-3	excluded	No comparison of arm movement in balance or postural control was conducted
619	Improving Trunk-Pelvis Stability Using Active Force Control at the Trunk and Passive Resistance at the Pelvis	Khan, MI; Santamaria, V; Agrawal, SK	Web of Science	IEEE ROBOTICS AND AUTOMATION LETTERS	2018	10.1109/LRA.2018.2809919	excluded	No comparison of arm movement in balance or postural control was conducted
620	Developing the Positional Characteristics of a Dance-Specific Star Excursion Balance Test (dsSEBT)	Beckman, S; Brouner, J	Web of Science	JOURNAL OF DANCE MEDICINE & SCIENCE	2022	10.12678/1089-313X.031522g	excluded	No comparison of arm movement in balance or postural control was conducted
621	Perturbation-based training enhances anticipatory postural control in individuals with chronic stroke: a pilot study	Curuk, E; Aruin, AS	Web of Science	INTERNATIONAL JOURNAL OF REHABILITATION RESEARCH	2022	10.1097/MRR.0000000000000515	excluded	not representative of a healthy population

622	Changes in and associations between functional indices of young handball players' upper extremities when applying neuromuscular training	Lendraitien, E; Petkut, T; Laurinaviciute, E	Web of Science	MEDICINA DELLO SPORT	2021	10.23736/S0025-7826.21.03908-9	excluded	No comparison of arm movement in balance or postural control was conducted
623	Exploring the effects of peripheral sensibility on visuospatial and postural capacities during goal-directed movements in long-term Tai Chi practitioners	Shao, ZF; Li, L; Mao, M; Sun, W; Zhang, C; Song, QP	Web of Science	FRONTIERS IN AGING NEUROSCIENCE	2022	10.3389/fnagi.2022.881972	excluded	No comparison of arm movement in balance or postural control was conducted
624	Upper Quarter Y-Balance Test in Collegiate Softball Players: Bilateral Arm Comparison and Influence of Reach Order	Christian, KM; Moran, MF	Web of Science	INTERNATIONAL JOURNAL OF ATHLETIC THERAPY & TRAINING	2021	10.1123/ijatt.2020-0064	excluded	No comparison of arm movement in balance or postural control was conducted
625	Injury Occurrence in Male Handball Players and its Impact on Physical Constitution	Salzer, S; Heansel, R; Rey, J; Maltry, L; Holzgreve, F; Lampe, J; Wanke, EM; Groneberg, DA; Ohlendorf, D	Web of Science	INTERNATIONAL JOURNAL OF SPORTS MEDICINE	2020	10.1055/a-1028-7630	excluded	No comparison of arm movement in balance or postural control was conducted
626	Does Fall Arrest Strategy Training Added to a Fall Prevention Programme Improve Balance, Strength, and Agility in Older Women? A Pilot Study	Arnold, CM; Walker-Johnston, J; Lanovaz, JL; Lattimer, LJ	Web of Science	PHYSIOTHERAPY CANADA	2017	10.3138/ptc.2016-27EP	excluded	No comparison of arm movement in balance or postural control was conducted

627	Altered Arm-Body Coordination with Triggered Pointing Responses as Influenced by Task Predictability	Prout, EC; Huntley, AH; Zettel, JL	Web of Science	JOURNAL OF MOTOR BEHAVIOR	2020	10.1080/00222895.2019.1596874	excluded	No comparison of arm movement (free vs restricted)
628	Spatial, But Not Temporal, Kinematics of Spontaneous Upper Extremity Movements Are Related to Gross and Fine Motor Skill Attainment in Infancy	Bican, R; Lowes, L; Alfano, L; McNally, M; Durbak, E; Pan, XL; Heathcock, J	Web of Science	JOURNAL OF MOTOR LEARNING AND DEVELOPMENT	2022	10.1123/jmld.2020-0035	excluded	No comparison of arm movement in balance or postural control was conducted
629	Time series analysis of spontaneous upper-extremity movements of premature infants with brain injuries	Ohgi, S; Morita, S; Loo, KK; Mizuike, C	Web of Science	PHYSICAL THERAPY	2008	10.2522/ptj.20070171	excluded	No comparison of arm movement in balance or postural control was conducted
630	Initiation of rapid reach-and-grasp balance reactions: is a pre-formed visuospatial map used in controlling the initial arm trajectory?	Ghafouri, M; McIlroy, WE; Maki, BE	Web of Science	EXPERIMENTAL BRAIN RESEARCH	2004	10.1007/s00221-004-1855-8	excluded	No comparison of arm movement (free vs restricted)
631	Does physical activity benefit motor performance and learning of upper extremity tasks in older adults? - A systematic review	Hübner, L; Voelcker-Rehage, C	Web of Science	EUROPEAN REVIEW OF AGING AND PHYSICAL ACTIVITY	2017	10.1186/s11556-017-0181-7	excluded	No comparison of arm movement in balance or postural control was conducted

632	Normative reference values for handgrip strength, shoulder and ankle range of motion and upper-limb and lower limb stability for 137 youth judokas of both sexes	Madaleno, FO; Verhagen, E; Ferreira, TV; Ribeiro, T; Ocarino, JM; Resende, RA	Web of Science	JOURNAL OF SCIENCE AND MEDICINE IN SPORT	2021	10.1016/j.jsams.2020.06.008	excluded	No comparison of arm movement in balance or postural control was conducted
633	Standing on Elevated Platform Changes Postural Reactive Responses during Arm Movement	Mochizuki, L; Pennone, J; Bigongiari, A; Cosme, RG; Massa, M; Ré, AHN; Alcântaro, RP Jr; Amadio, AC	Web of Science	BRAIN SCIENCES	2024	10.3390/brainsci14101004	excluded	No comparison of arm movement (free vs restricted)
634	Test-Retest Reliability of Single Leg Jump Performance Using the Drift Protocol in Division I Baseball Pitchers	Bergquist, AM; Lebron, MA; Mangum, LC; Stout, JR; Fukuda, DH	Web of Science	JOURNAL OF SCIENCE IN SPORT AND EXERCISE	2023	10.1007/s42978-023-00243-y	excluded	No comparison of arm movement in balance or postural control was conducted
635	Pain differs from non-painful attention-demanding or stressful tasks in its effect on postural control patterns of trunk muscles	Moseley, GL; Nicholas, MK; Hodges, PW	Web of Science	EXPERIMENTAL BRAIN RESEARCH	2004	10.1007/s00221-003-1766-0	excluded	No comparison of arm movement in balance or postural control was conducted
636	Changes in the control of arm position, movement, and thalamic discharge during local inactivation in the globus pallidus of the monkey	Inase, M; Buford, JA; Anderson, ME	Web of Science	JOURNAL OF NEUROPHYSIOLOGY	1996	10.1152/jn.1996.75.3.1087	excluded	monkey

637	1A complete, non-lumped, and verifiable set of upper body segment parameters for three-dimensional dynamic modeling	Vette, AH; Yoshida, T; Thrasher, TA; Masani, K; Popovic, MR	Web of Science	MEDICAL ENGINEERING & PHYSICS	2011	10.1016/j.medengphy.2010.09.008	excluded	No comparison of arm movement in balance or postural control was conducted
638	Influence of obesity on accurate and rapid arm movement performed from a standing posture	Berrigan, F; Simoneau, M; Tremblay, A; Hue, O; Teasdale, N	Web of Science	INTERNATIONAL JOURNAL OF OBESITY	2006	10.1038/sj.ijo.0803342	excluded	No comparison of arm movement (free vs restricted)
639	Tai Chi Can Improve Postural Stability as Measured by Resistance to Perturbation Related to Upper Limb Movement among Healthy Older Adults	Pan, JH; Liu, CX; Zhang, SQ; Li, L	Web of Science	EVIDENCE-BASED COMPLEMENTARY AND ALTERNATIVE MEDICINE	2016	10.1155/2016/9710941	excluded	No comparison of arm movement (free vs restricted)
640	Developing a Medical Garment for Upper-Body Posture-Related Issues	Ranawaka, RAHS; Kularatne, SDMW; Fernando, EASK; Niles, SN; Jayawardane, TSS; Ranaweera, RKPS	Web of Science	TEKSTILEC	2024	10.14502/tekstilec.67.2024002	excluded	No comparison of arm movement in balance or postural control was conducted
641	Constraints of Load and Posture on Coordination Variability and Marksmanship Performance	Palmer, CJ; van Emmerik, REA	Web of Science	MOTOR CONTROL	2020	10.1123/mc.2019-0082	excluded	No comparison of arm movement in balance or postural control was conducted

642	The effect of walking and stationary work on the acute back pain, muscle activation, posture and postural control of older women	Arkesteijn, M; Jones, R; Low, DC	Web of Science	ERGONOMICS	2022	10.1080/00140139.2021.2000044	excluded	No comparison of arm movement in balance or postural control was conducted
643	Correlations between core muscle strength endurance and upper-extremity performance in adolescent male sub-elite handball players	Bauer, J; Gruber, M; Muehlbauer, T	Web of Science	FRONTIERS IN SPORTS AND ACTIVE LIVING	2022	10.3389/fspor.2022.1050279	excluded	No comparison of arm movement in balance or postural control was conducted
644	Influence of Different Sitting Positions on Healthy Infants' Reaching Movements	da Silva, ESM; dos Santos, GL; Greco, ALR; Tudella, E	Web of Science	JOURNAL OF MOTOR BEHAVIOR	2017	10.1080/00222895.2016.1247034	excluded	No comparison of arm movement in balance or postural control was conducted
645	The use of peripheral vision to guide perturbation-evoked reach-to-grasp balance-recovery reactions	King, EC; McKay, SM; Cheng, KC; Maki, BE	Web of Science	EXPERIMENTAL BRAIN RESEARCH	2010	10.1007/s00221-010-2434-9	excluded	No comparison of arm movement (free vs restricted)
646	Slower visuomotor reaction time in division-I collegiate athletes with a history of ankle sprain	Song, K; Hoch, JM; Quintana, C; Heebner, NR; Hoch, MC	Web of Science	RESEARCH IN SPORTS MEDICINE	2023	10.1080/15438627.2021.1996361	excluded	No comparison of arm movement in balance or postural control was conducted
647	The Effects of Vision-Deprived Progressive Resistance Training on One-Repetition Maximum Bench Press Performance: An Exploratory Study	Boolani, A; Moghaddam, M; Fuller, D; Mondal, S; Sur, S; Martin, R; Kadry, A; Torad, AA; Elwan, MA; Kakar, RS	Web of Science	VISION	2022	10.3390/vision6030047	excluded	No comparison of arm movement in balance or postural control was conducted

648	Balance recovery from medio-lateral perturbations of the upper body during standing	Rietdyk, S; Patla, AE; Winter, DA; Ishac, MG; Little, CE	Web of Science	JOURNAL OF BIOMECHANICS	1999	10.1016/S0021-9290(99)00116-5	excluded	No comparison of arm movement (free vs restricted)
649	The Impact of Dance-Specific Neuromuscular Conditioning and Injury Prevention Training on Motor Control, Stability, Balance, Function and Injury in Professional Ballet Dancers: A Mixed-Methods Quasi-Experimental Study	Long, KL; Milidonis, MK; Wildermuth, VL; Kruse, AN; Parham, UT	Web of Science	INTERNATIONAL JOURNAL OF SPORTS PHYSICAL THERAPY	2021		excluded	No comparison of arm movement in balance or postural control was conducted
650	Trunk stabilization, body balance, body perception, and quality of life in professional physically disabled and able-bodied archers	Arkin, I; Budak, M	Web of Science	SPORT SCIENCES FOR HEALTH	2021	10.1007/s11332-021-00744-9	excluded	No comparison of arm movement in balance or postural control was conducted
651	Activation timing of postural muscles during bilateral arm flexion in self-timing, oddball and simple-reaction tasks	Fujiwara, K; Yaguchi, C; Shen, XZ; Maeda, K; Mammadova, A	Web of Science	JOURNAL OF ELECTROMYOGRAPHY AND KINESIOLOGY	2011	10.1016/j.jelekin.2011.04.002	excluded	No comparison of arm movement in balance or postural control was conducted

652	Difference in Functional Performance on the Upper-Quarter Y-Balance Test Between High School Baseball Players and Wrestlers	Myers, H; Poletti, M; Butler, RJ	Web of Science	JOURNAL OF SPORT REHABILITATION	2017	10.1123/jsr.2015-0168	excluded	No comparison of arm movement in balance or postural control was conducted
653	Therapeutic effects of brain-computer interface-controlled functional electrical stimulation training on balance and gait performance for stroke A pilot randomized controlled trial	Chung, EJ; Lee, BH; Hwang, S	Web of Science	MEDICINE	2020	10.1097/MD.0000000000022612	excluded	No comparison of arm movement in balance or postural control was conducted
654	Is whole-body vibration beneficial for seniors?	Lachance, C; Weir, P; Kenno, K; Horton, S	Web of Science	EUROPEAN REVIEW OF AGING AND PHYSICAL ACTIVITY	2012	10.1007/s11556-011-0094-9	excluded	No comparison of arm movement in balance or postural control was conducted
655	More Falls in Cerebellar Ataxia When Standing on a Slow Up-Moving Tilt of the Support Surface	Paquette, C; Franzén, E; Horak, FB	Web of Science	CEREBELLUM	2016	10.1007/s12311-015-0704-6	excluded	No comparison of arm movement in balance or postural control was conducted
656	Associations Between Functional Movement Screening, the Y Balance Test, and Injuries in Coast Guard Training	Cosio-Lima, L; Knapik, JJ; Shumway, R; Reynolds, K; Lee, Y; Greska, E; Hampton, M	Web of Science	MILITARY MEDICINE	2016	10.7205/MILMED-D-15-00208	excluded	No comparison of arm movement in balance or postural control was conducted

657	Factors associated with thoracic spinal cord injury, lesion level and rotator cuff disorders	Sinnott, KA; Milburn, P; McNaughton, H	Web of Science	SPINAL CORD	2000	10.1038/sj.sc.3101095	excluded	No comparison of arm movement in balance or postural control was conducted
658	Movement patterns and muscular demands during posterior transfers toward an elevated surface in individuals with spinal cord injury	Gagnon, D; Nadeau, S; Gravel, D; Noreau, L; Larivière, C; McFadyen, B	Web of Science	SPINAL CORD	2005	10.1038/sj.sc.3101660	excluded	No comparison of arm movement in balance or postural control was conducted
659	Motor alterations along the kinetic chain in amateur volleyball and handball athletes with shoulder pain: An observational comparative study	Barros, BRD; de Barros, ACM; da Silva, N Jr; Cavalcanti, IBS; Sousa, CD	Web of Science	JOURNAL OF BODYWORK AND MOVEMENT THERAPIES	2024	10.1016/j.jbmt.2024.02.002	excluded	No comparison of arm movement in balance or postural control was conducted
660	Ergonomic Evaluation of Sitting Postures in School Chairs of School-Age Children with Special Needs	Cemali, M; Tunç, AR; Cimilli, E; Alatas, DM; Arslan, BÇ; Karaduman, AA	Web of Science	JOURNAL OF OCCUPATIONAL THERAPY SCHOOLS AND EARLY INTERVENTION	2024	10.1080/19411243.2024.2333282	excluded	No comparison of arm movement in balance or postural control was conducted
661	Significance of adequate postural control in the appearance of habitual upright bipedal locomotion	Sekulic, S; Podgorac, J; Kekovic, G; Zarkov, M; Kopitovic, A	Web of Science	MEDICAL HYPOTHESES	2012	10.1016/j.mehy.2012.07.019	excluded	No comparison of arm movement in balance or postural control was conducted

662	The Relationship Between Trunk Rotation, Upper Quarter Dynamic Stability, and Pitch Velocity	Bullock, GS; Schmitt, AC; Chasse, PM; Little, BA; Diehl, LH; Butler, RJ	Web of Science	JOURNAL OF STRENGTH AND CONDITIONING RESEARCH	2018	10.1519/JSC.0000000000001772	excluded	No comparison of arm movement in balance or postural control was conducted
663	Effects of a Pilates exercise program on muscle strength, postural control and body composition: results from a pilot study in a group of post-menopausal women	Bergamin, M; Gobbo, S; Bullo, V; Zanotto, T; Vendramin, B; Duregon, F; Cugusi, L; Camozzi, V; Zaccaria, M; Neunhaeuserer, D; Ermolao, A	Web of Science	AGE	2015	10.1007/s11357-015-9852-3	excluded	No comparison of arm movement in balance or postural control was conducted
664	The content and effects of trunk rehabilitation on trunk and upper limb performance in people with multiple sclerosis: a systematic review	Raats, J; Lamers, I; Merken, I; Boeckmans, J; Soler, B; Normann, B; Arntzen, EC; Feys, P	Web of Science	EUROPEAN JOURNAL OF PHYSICAL AND REHABILITATION MEDICINE	2022	10.23736/S1973-9087.21.06689-2	excluded	not representative of a healthy population
665	The feasibility and efficacy of a serial reaction time task that measures motor learning of anticipatory stepping	Olivier, GN; Paul, SS; Walter, CS; Hayes, HA; Foreman, KB; Duff, K; Schaefer, SY; Dibble, LE	Web of Science	GAIT & POSTURE	2021	10.1016/j.gaitpost.2021.04.002	excluded	No comparison of arm movement in balance or postural control was conducted
666	The Effect of Additional Leg Supports in Control of Posture in Sitting	Ademiluyi, A; Liang, HQ; Aruin, AS	Web of Science	JOURNAL OF MOTOR BEHAVIOR	2023	10.1080/00222895.2023.2181751	excluded	No comparison of arm movement in balance or postural control was conducted

667	Postural and respiratory functions of the pelvic floor muscles	Hodges, PW; Sapsford, R; Pengel, LHM	Web of Science	NEUROUROLOGY AND URODYNAMICS	2007	10.1002/nau.20232	excluded	No comparison of arm movement in balance or postural control was conducted
668	Effects of core stability and feedback music on upper body mediolateral movements during cycling	Jeong, S; Kim, SH; Park, KN	Web of Science	BMC SPORTS SCIENCE MEDICINE AND REHABILITATION	2024	10.1186/s13102-024-00822-8	excluded	No comparison of arm movement in balance or postural control was conducted
669	Development of postural adaptation to arm raising	Hay, L; Redon, C	Web of Science	EXPERIMENTAL BRAIN RESEARCH	2001	10.1007/s002210100752	excluded	No comparison of arm movement (free vs restricted)
670	Control of posture with FES systems	Matjacic, Z; Hunt, K; Gollee, H; Sinkjaer, T	Web of Science	MEDICAL ENGINEERING & PHYSICS	2003	10.1016/S1350-4533(02)00115-7	excluded	No comparison of arm movement in balance or postural control was conducted
671	Immediate impact of extremity manipulation on dual task performance: a randomized, crossover clinical trial	Malaya, CA; Haworth, J; Pohlman, KA; Smith, DL	Web of Science	CHIROPRACTIC & MANUAL THERAPIES	2021	10.1186/s12998-021-00366-5	excluded	No comparison of arm movement in balance or postural control was conducted
672	Nonlinear response analysis of the human ligamentous lumbar spine in compression. On mechanisms affecting the postural stability	SHIRAZIADL, A; PARNIANPOUR, M	Web of Science	SPINE	1993	10.1097/00007632-199301000-00021	excluded	No comparison of arm movement in balance or postural control was conducted

673	The amplitude of interlimb cutaneous reflexes in the leg is influenced by fingertip touch and vision during treadmill locomotion	Forero, J; Misiaszek, JE	Web of Science	EXPERIMENTAL BRAIN RESEARCH	2015	10.1007/s00221-015-4250-8	excluded	No comparison of arm movement in balance or postural control was conducted
674	**The effect of arm movements on the lower limb during gait after a stroke**	**Stephenson, JL; De Serres, SJ; Lamontagne, A**	**Web of Science**	**GAIT & POSTURE**	**2010**	**10.1016/j.gaitpost.2009.09.008**	**included**	
675	Whole body adaptation to novel dynamics does not transfer between effectors	Pienciak-Siewert, A; Ahmed, AA	Web of Science	JOURNAL OF NEUROPHYSIOLOGY	2021	10.1152/jn.00628.2020	excluded	No comparison of arm movement in balance or postural control was conducted
676	Resolving conflicts in task demands during balance recovery: does holding an object inhibit compensatory grasping?	Bateni, H; Zecevic, A; McIlroy, WE; Maki, BE	Web of Science	EXPERIMENTAL BRAIN RESEARCH	2004	10.1007/s00221-003-1815-8	excluded	No comparison of arm movement in balance or postural control was conducted
677	Positive effects of 1-year football and strength training on mechanical muscle function and functional capacity in elderly men	Sundstrup, E; Jakobsen, MD; Andersen, LL; Andersen, TR; Randers, MB; Helge, JW; Suetta, C; Schmidt, JF; Bangsbo, J; Krustrup, P; Aagaard, P	Web of Science	EUROPEAN JOURNAL OF APPLIED PHYSIOLOGY	2016	10.1007/s00421-016-3368-0	excluded	No comparison of arm movement in balance or postural control was conducted

678	Sensory function and somatosensorial system changes according to visual acuity and throwing techniques in goalball players: A cross-sectional study	Göksen, A; Ince, G	Web of Science	PLOS ONE	2024	10.1371/journal.pone.0296948	excluded	No comparison of arm movement in balance or postural control was conducted
679	Lower Extremity Musculoskeletal Injuries After Concussion in Collegiate Student-Athletes	Buckley, TA; Chandran, A; Mauntel, TC; Kerr, ZY; Brown, DW; Boltz, AJ; Herman, DC; Hall, EE; Lynall, RC	Web of Science	AMERICAN JOURNAL OF SPORTS MEDICINE	2023	10.1177/03635465221125155	excluded	No comparison of arm movement in balance or postural control was conducted
680	Multimodal cortical and subcortical exercise compared with treadmill training for spinal cord injury	Martinez, SA; Nguyen, ND; Bailey, E; Doyle-Green, D; Hauser, HA; Handrakis, JP; Knezevic, S; Marett, C; Weinman, J; Romero, AF; Santiago, TM; Yang, AH; Yung, L; Asselin, PK; Weir, JP; Kornfeld, SD; Bauman, WA; Spungen, AM; Harel, NY	Web of Science	PLOS ONE	2018	10.1371/journal.pone.0202130	excluded	No comparison of arm movement in balance or postural control was conducted
681	Immersive virtual reality interferes with default head-trunk coordination strategies in young children	Miehlbradt, J; Cuturi, LF; Zanchi, S; Gori, M; Micera, S	Web of Science	SCIENTIFIC REPORTS	2021	10.1038/s41598-021-96866-8	excluded	No comparison of arm movement in balance or postural control was conducted

682	The Effect of Unsupervised Home-Based Exercise Training on Physical Functioning Outcomes in Older Adults: A Systematic Review and Meta-Analysis of Randomized Controlled Trials	Mahjur, M; Norasteh, AA	Web of Science	BIOLOGICAL RESEARCH FOR NURSING	2021	10.1177/1099800421989439	excluded	No comparison of arm movement in balance or postural control was conducted
683	Alcohol intoxication at 0.06 and 0.10% blood alcohol concentration changes segmental body movement coordination	Patel, M; Modig, F; Magnusson, M; Fransson, PA	Web of Science	EXPERIMENTAL BRAIN RESEARCH	2010	10.1007/s00221-009-2150-5	excluded	No comparison of arm movement in balance or postural control was conducted
684	Modulation of intrinsic and reflexive contributions to low-back stabilization due to vision, task instruction, and perturbation bandwidth	van Drunen, P; Koumans, Y; van der Helm, FCT; van Dieën, JH; Happee, R	Web of Science	EXPERIMENTAL BRAIN RESEARCH	2015	10.1007/s00221-014-4151-2	excluded	No comparison of arm movement in balance or postural control was conducted
685	Assessment of postural response after a self-initiated perturbation	Termoz, N; Martin, L; Prince, F	Web of Science	MOTOR CONTROL	2004	10.1123/mcj.8.1.51	excluded	No comparison of arm movement (free vs restricted)
686	Effects of additional external weight on posture and movement adaptations to fatigue induced by a repetitive pointing task	Cantú, H; Emery, K; Côté, JN	Web of Science	HUMAN MOVEMENT SCIENCE	2014	10.1016/j.humov.2014.02.003	excluded	No comparison of arm movement (free vs restricted)

687	Study of the effects of multimodal exercise program on physical fitness and health perception in community-living Hungarian older adults	Virág, A; Harkányi, I; Karóczi, CK; Vass, Z; Kovács, É	Web of Science	JOURNAL OF SPORTS MEDICINE AND PHYSICAL FITNESS	2018	10.23736/S0022-4707.17.07492-8	excluded	No comparison of arm movement in balance or postural control was conducted
688	Age-related changes in the capacity to select early-onset upper-limb reactions to either recover balance or protect against impact	Borrelli, JR; Zabukovec, J; Jones, S; Junod, CA; Maki, BE	Web of Science	EXPERIMENTAL GERONTOLOGY	2019	10.1016/j.exger.2019.110676	excluded	No comparison of arm movement (free vs restricted)
689	Effect of a plantar perceptual learning task on walking stability in the elderly: a randomized controlled trial	Nakano, H; Nozaki, M; Ueta, K; Osumi, M; Kawami, S; Morioka, S	Web of Science	CLINICAL REHABILITATION	2013	10.1177/0269215512471062	excluded	No comparison of arm movement in balance or postural control was conducted
690	Transversus abdominis and the superficial abdominal muscles are controlled independently in a postural task	Hodges, PW; Richardson, CA	Web of Science	NEUROSCIENCE LETTERS	1999	10.1016/S0304-3940(99)00216-5	excluded	No comparison of arm movement in balance or postural control was conducted
691	Innovative Use of Biodex Balance System to Improve Dynamic Stabilization and Function of Upper Quarter in Recreational Weightlifters: A Randomized Controlled Trial	Abdelraouf, OR; Abdel-aziem, AA; Ghally, SA; Osama, LA; Dawood, RS; Yehia, AM; Eed, EM; El-Gendy, AM; Radwan, RE	Web of Science	MEDICINA-LITHUANIA	2022	10.3390/medicina58111631	excluded	No comparison of arm movement in balance or postural control was conducted

692	Effect of Eight Weeks of Reformer Pilates on Shoulder Proprioception Dynamic Stability and Functionality	Altunalan, T; Çalik, M; Kapansahin, M	Web of Science	BEZMIALEM SCIENCE	2024	10.14235/bas.galenos.2024.46363	excluded	No comparison of arm movement in balance or postural control was conducted
693	Investigating the Effects of Center of Gravity (CoG) Shift Due to a Simulated Exploration Extravehicular Mobility Unit (xEMU) Suit on Balance	Melendez, RAR; Thompson, LA	Web of Science	APPLIED SCIENCES-BASEL	2024	10.3390/app14104032	excluded	No comparison of arm movement in balance or postural control was conducted
694	Identifying intrinsic and reflexive contributions to low-back stabilization	van Drunen, P; Maaswinkel, E; van der Helm, FCT; van Dieën, JH; Happee, R	Web of Science	JOURNAL OF BIOMECHANICS	2013	10.1016/j.jbiomech.2013.03.007	excluded	No comparison of arm movement in balance or postural control was conducted
695	Reaching to recover balance in unpredictable circumstances: Is online visual control of the reach-to-grasp reaction necessary or sufficient?	Cheng, KC; McKay, SM; King, EC; Maki, BE	Web of Science	EXPERIMENTAL BRAIN RESEARCH	2012	10.1007/s00221-012-3051-6	excluded	No comparison of arm movement (free vs restricted)
696	Learning to balance on a slackline: Development of coordinated multi-joint synergies	Mildren, RL; Zaback, M; Adkin, AL; Bent, LR; Frank, JS	Web of Science	SCANDINAVIAN JOURNAL OF MEDICINE & SCIENCE IN SPORTS	2018	10.1111/sms.13208	excluded	No comparison of arm movement in balance or postural control was conducted

697	Acute Hip Abduction Fatigue on Lumbopelvic-Hip Complex Stability in Softball Players	Washington, J; Gilmer, G; Oliver, G	Web of Science	INTERNATIONAL JOURNAL OF SPORTS MEDICINE	2018	10.1055/a-0577-3722	excluded	No comparison of arm movement in balance or postural control was conducted
698	Biomechanical approach to quantifying anticipatory postural adjustments in the elderly	MAKI, BE	Web of Science	MEDICAL & BIOLOGICAL ENGINEERING & COMPUTING	1993	10.1007/BF02446688	excluded	No comparison of arm movement (free vs restricted)
699	Absence of equifinality of hand position in a double-step unloading task	Norouzi-Gheidari, N; Archambault, P	Web of Science	EXPERIMENTAL BRAIN RESEARCH	2010	10.1007/s00221-010-2350-z	excluded	No comparison of arm movement in balance or postural control was conducted
700	Changes in postural mechanics associated with different types of minimally invasive surgical training exercises	Gillette, JC; Quick, NE; Adrales, GL; Shapiro, R; Park, AE	Web of Science	SURGICAL ENDOSCOPY AND OTHER INTERVENTIONAL TECHNIQUES	2003	10.1007/s00464-002-8842-6	excluded	No comparison of arm movement in balance or postural control was conducted
701	The Influence of Motor Imagery on Postural Sway: Differential Effects of Type of Body Movement and Person Perspective	Stins, JF; Schneider, IK; Koole, SL; Beek, PJ	Web of Science	ADVANCES IN COGNITIVE PSYCHOLOGY	2015	10.5709/acp-0173-x	excluded	No comparison of arm movement in balance or postural control was conducted

702	Effects of Yoga Training Applied with Telerehabilitation on Core Stabilization and Physical Fitness in Junior Tennis Players: A Randomized Controlled Trial	Gerguez, C; Bayram, GA	Web of Science	COMPLEMENTARY MEDICINE RESEARCH	2023	10.1159/000533848	excluded	No comparison of arm movement in balance or postural control was conducted
703	Eliminating toe-fixing pattern can improve standing and gait pattern of children with cerebral palsy in a qualitative way	Chan, TWT; Law, SH	Web of Science	INTERNATIONAL JOURNAL OF REHABILITATION RESEARCH	2008	10.1097/MRR.0b013e3282fb7857	excluded	No comparison of arm movement in balance or postural control was conducted
704	The role of age, sex, anthropometry, and body composition as determinants of physical fitness in nonobese children aged 6-12	Milanese, C; Sandri, M; Cavedon, V; Zancanaro, C	Web of Science	PEERJ	2020	10.7717/peerj.8657	excluded	No comparison of arm movement in balance or postural control was conducted
705	The development of postural adjustments during reaching in 6-to 18-month-old infants - Evidence for two transitions	Van der Fits, IBM; Klip, AWJ; Van Eykern, LA; Hadders-Algra, M	Web of Science	EXPERIMENTAL BRAIN RESEARCH	1999	10.1007/s002210050760	excluded	No comparison of arm movement in balance or postural control was conducted
706	Postural activity of the diaphragm is reduced in humans when respiratory demand increases	Hodges, PW; Heijnen, I; Gandevia, SC	Web of Science	JOURNAL OF PHYSIOLOGY-LONDON	2001		excluded	No comparison of arm movement in balance or postural control was conducted

707	A Comparison of Injury Risk Screening Tools in Turkish Young Elite Male Handball Players Based on Field Positions	Koçak, UZ; Ünver, B; Özer Kaya, D	Web of Science	TURKISH JOURNAL OF PHYSIOTHERAPY REHABILITATION-TURK FIZYOTERAPI VE REHABILITASYON DERGISI	2020	10.21653/tjpr.583463	excluded	No comparison of arm movement in balance or postural control was conducted
708	Changes in motor planning of feedforward postural responses of the trunk muscles in low back pain	Hodges, PW	Web of Science	EXPERIMENTAL BRAIN RESEARCH	2001	10.1007/s002210100873	excluded	No comparison of arm movement in balance or postural control was conducted
709	Female Collegiate Dancers' Physical Fitness across Their Four-Year Programs: A Prospective Analysis	Ambegaonkar, JP; Hansen-Honeycutt, J; Wiese, KR; Cavanagh, CM; Caswell, SV; Ambegaonkar, SJ; Martin, J	Web of Science	JOURNAL OF FUNCTIONAL MORPHOLOGY AND KINESIOLOGY	2023	10.3390/jfmk8030098	excluded	No comparison of arm movement in balance or postural control was conducted
710	An investigation into the influence of biological sex, anthropometrics, footwear, and dual tasking on balance	Aljawaee, M; Jones, MD; Williams, JM	Web of Science	PHYSIOTHERAPY PRACTICE AND RESEARCH	2024	10.3233/PPR-230806	excluded	No comparison of arm movement in balance or postural control was conducted

711	Effect of Leg Dominance on The Center-of-Mass Kinematics During an Inside-of-the-Foot Kick in Amateur Soccer Players	Zago, M; Motta, AF; Mapelli, A; Annoni, I; Galvani, C; Sforza, C	Web of Science	JOURNAL OF HUMAN KINETICS	2014	10.2478/hukin-2014-0060	excluded	No comparison of arm movement in balance or postural control was conducted
712	Ipsilateral versus contralateral static endurance- balance abilities among healthy college students	El-gohary, TM; Al-Shenqiti, AM; Alshehri, YS; Aljohani, MM	Web of Science	JOURNAL OF TAIBAH UNIVERSITY MEDICAL SCIENCES	2024	10.1016/j.jtumed.2023.12.009	excluded	No comparison of arm movement in balance or postural control was conducted
713	Role of heel lifting in standing balance recovery: A simulation study	Cheng, KB; Tanabe, H; Chen, WC; Chiu, HT	Web of Science	JOURNAL OF BIOMECHANICS	2018	10.1016/j.jbiomech.2017.11.020	excluded	No comparison of arm movement in balance or postural control was conducted
714	Age-related differences in body segmental movement during perturbed stance in humans	Wu, G	Web of Science	CLINICAL BIOMECHANICS	1998	10.1016/S0268-0033(98)00068-0	excluded	No comparison of arm movement (free vs restricted)
715	Sensory contributions to stabilization of trunk posture in the sagittal plane	van Dieën, JH; van Drunen, P; Happee, R	Web of Science	JOURNAL OF BIOMECHANICS	2018	10.1016/j.jbiomech.2017.07.016	excluded	No comparison of arm movement in balance or postural control was conducted
716	Biomechanical and physiological age differences in a simulated forward fall on outstretched hands in women	Lattimer, LJ; Lanovaz, JL; Farthing, JP; Madill, S; Kim, SY; Robinovitch, S; Arnold, CM	Web of Science	CLINICAL BIOMECHANICS	2018	10.1016/j.clinbiomech.2018.01.018	excluded	No comparison of arm movement in balance or postural control was conducted
717	Postural muscle activity during bilateral and unilateral arm movements at different speeds	Mochizuki, G; Ivanova, TD; Garland, SJ	Web of Science	EXPERIMENTAL BRAIN RESEARCH	2004	10.1007/s00221-003-1732-x	excluded	No comparison of arm movement in balance or postural control was conducted

718	Postural Strategies Used While Donning a Simulated xEMU Spacesuit	Melendez, RAR; Thompson, LA	Web of Science	APPLIED SCIENCES-BASEL	2024	10.3390/app14198773	excluded	No comparison of arm movement in balance or postural control was conducted
719	Factors affecting the shoulder functional profile in elite judo athletes	Delorme, J; Blache, Y; Degot, M; Rogowski, I	Web of Science	EUROPEAN JOURNAL OF SPORT SCIENCE	2023	10.1080/17461391.2022.2069511	excluded	No comparison of arm movement in balance or postural control was conducted
720	Postural adjustments to self-triggered perturbations under conditions of changes in body orientation	Pascucci, F; Cesari, P; Bertucco, M; Latash, ML	Web of Science	EXPERIMENTAL BRAIN RESEARCH	2023	10.1007/s00221-023-06671-0	excluded	No balance or postural control was conducted
721	Anticipatory EMG patterns associated with preferred and non-preferred arm pointing movements	Teyssèdre, C; Lino, F; Zattara, M; Bouisset, S	Web of Science	EXPERIMENTAL BRAIN RESEARCH	2000	10.1007/s002210000490	excluded	No comparison of arm movement in balance or postural control was conducted
722	Neuromuscular but Not Technical Performance is Affected by Time-of-Day in Semiprofessional, Female Basketball Players	Gaos, S; Sánchez-Jorge, S; Munoz, A; Vicente-Campos, D; Acebes-Sánchez, J; Esquius, L; Scanlan, AT; López-Samanes, A	Web of Science	RESEARCH QUARTERLY FOR EXERCISE AND SPORT	2024	10.1080/02701367.2023.2265447	excluded	No comparison of arm movement in balance or postural control was conducted
723	Predicting reactive stepping in response to perturbations by using a classification approach	Emmens, AR; van Asseldonk, EHF; Prinsen, V; van der Kooij, H	Web of Science	JOURNAL OF NEUROENGINEERING AND REHABILITATION	2020	10.1186/s12984-020-00709-y	excluded	No comparison of arm movement in balance or postural control was conducted

724	Thinking on your feet: An analysis of movement and cognition in a sit to stand task	Gibbons, CT; Amazeen, PG; Jondac, JJ	Web of Science	ACTA PSYCHOLOGICA	2019	10.1016/j.actpsy.2018.10.014	excluded	No comparison of arm movement in balance or postural control was conducted
725	The effects of stance configuration and target distance on reaching I. Movement preparation	Kaminski, TR; Simpkins, S	Web of Science	EXPERIMENTAL BRAIN RESEARCH	2001	10.1007/s002210000604	excluded	No comparison of arm movement in balance or postural control was conducted
726	Age and initial position affect movement biomechanics in sit to walk transitions: Whole body balance and trunk control	Miller, MF; van der Kruk, E; Silverman, AK	Web of Science	JOURNAL OF BIOMECHANICS	2024	10.1016/j.jbiomech.2024.112256	excluded	No comparison of arm movement in balance or postural control was conducted
727	Normative values and factors affecting Pediatric Reach Tests in Saudi children aged 6-11 years in the eastern province: cross-sectional study	Alotaibi, RS; Algabbani, MF; Shaheen, AAM; Albishi, AM; Almurdi, MM	Web of Science	FRONTIERS IN PEDIATRICS	2024	10.3389/fped.2023.1240659	excluded	No comparison of arm movement in balance or postural control was conducted
728	Ankle foot orthoses in cerebral palsy: Effects of ankle stiffness on trunk kinematics, gait stability and energy cost of walking	Meyns, P; Kerkum, YL; Brehm, MA; Becher, JG; Buizer, AI; Harlaar, J	Web of Science	EUROPEAN JOURNAL OF PAEDIATRIC NEUROLOGY	2020	10.1016/j.ejpn.2020.02.009	excluded	No comparison of arm movement in balance or postural control was conducted
729	Activation timing of postural muscles of lower legs and prediction of postural disturbance during bilateral arm flexion in older adults	Yaguchi, C; Fujiwara, K; Kiyota, N	Web of Science	JOURNAL OF PHYSIOLOGICAL ANTHROPOLOGY	2017	10.1186/s40101-017-0160-8	excluded	no balance parameter

730	Soft Neurological Signs in Childhood by Measurement of Arm Movements Using Acceleration and Angular Velocity Sensors	Kaneko, M; Yamashita, Y; Inomoto, O; Iramina, K	Web of Science	SENSORS	2015	10.3390/s151025793	excluded	focused on pronation and supination
731	Physical Fitness and Dynamic Balance in Medication Naive Turkish Children with ADHD	Buker, N; Sengul, YS; Ozbek, A	Web of Science	PERCEPTUAL AND MOTOR SKILLS	2020	10.1177/0031512520938517	excluded	No comparison of arm movement in balance or postural control was conducted
732	The effects of muscle vibration on anticipatory postural adjustments	Slijper, H; Latash, ML	Web of Science	BRAIN RESEARCH	2004	10.1016/j.brainres.2004.04.054	excluded	No comparison of arm movement in balance or postural control was conducted
733	Proximal and distal movement patterns during a graphomotor task in typically developing children and children with handwriting problems	Steinhart, S; Weiss, PL; Friedman, J	Web of Science	JOURNAL OF NEUROENGINEERING AND REHABILITATION	2021	10.1186/s12984-021-00970-9	excluded	No comparison of arm movement in balance or postural control was conducted
734	Static and dynamic evaluation of the influence of supplementary hip-joint stiffness on crutch-supported paraplegic stance	van der Spek, JH; Veltink, PH; Hermens, HJ; Koopman, BFJM; Boom, HBK	Web of Science	IEEE TRANSACTIONS ON NEURAL SYSTEMS AND REHABILITATION ENGINEERING	2003	10.1109/TNSRE.2003.819940	excluded	No comparison of arm movement in balance or postural control was conducted

735	Alterations of Muscular Strength and Left and Right Limb Balance in Weightlifters after an 8-week Balance Training Program	Kang, SH; Kim, CW; Kim, YI; Kim, KB; Lee, SS; Shin, KO	Web of Science	JOURNAL OF PHYSICAL THERAPY SCIENCE	2013	10.1589/jpts.25.895	excluded	No comparison of arm movement in balance or postural control was conducted
736	Does risk-sensitivity transfer across movements?	O'Brien, MK; Ahmed, AA	Web of Science	JOURNAL OF NEUROPHYSIOLOGY	2013	10.1152/jn.00826.2012	excluded	No comparison of arm movement in balance or postural control was conducted
737	Comparison of upper and lower body dynamic balance between sport climbers and non-climbers	Slak, V; Kozinc, Z	Web of Science	KINESIOLOGIA SLOVENICA	2024	10.52165/kinsi.30.3.143-157	excluded	No comparison of arm movement in balance or postural control was conducted
738	An Intensive Intervention for Improving Gait, Balance, and Mobility in Individuals With Chronic Incomplete Spinal Cord Injury: A Pilot Study of Activity Tolerance and Benefits	Fritz, SL; Merlo-Rains, AM; Rivers, ED; Peters, DM; Goodman, A; Watson, ET; Carmichael, BM; McClenaghan, BA	Web of Science	ARCHIVES OF PHYSICAL MEDICINE AND REHABILITATION	2011	10.1016/j.apmr.2011.05.006	excluded	No comparison of arm movement in balance or postural control was conducted
739	Predicting Upper Quadrant Musculoskeletal Injuries in the Military: A Cohort Study	Campbell, KE; Parent, EC; Crumback, DJ; Hebert, JS	Web of Science	MEDICINE & SCIENCE IN SPORTS & EXERCISE	2022	10.1249/MSS.0000000000002789	excluded	No comparison of arm movement in balance or postural control was conducted
740	Effects of athletic training on physical fitness and stroke velocity in healthy youth and adult tennis players: A systematic review and meta-analysis	Lambrich, J; Muehlbauer, T	Web of Science	FRONTIERS IN SPORTS AND ACTIVE LIVING	2023	10.3389/fspor.2022.1061087	excluded	No comparison of arm movement in balance or postural control was conducted

741	Upper limb and trunk muscle activation during an unexpected descent on the outstretched hands in young and older women	Lattimer, LJ; Lanovaz, JL; Farthing, JP; Madill, S; Kim, S; Arnold, C	Web of Science	JOURNAL OF ELECTROMYOGRAPHY AND KINESIOLOGY	2016	10.1016/j.jelekin.2016.08.001	excluded	No comparison of arm movement in balance or postural control was conducted
742	The control of body orientation and center of mass location under asymmetrical loading	Wu, G; MacLeod, M	Web of Science	GAIT & POSTURE	2001	10.1016/S0966-6362(00)00102-8	excluded	No comparison of arm movement in balance or postural control was conducted
743	The interactions between agonist-to-antagonist muscle strength performance and plantar pressure distribution, foot contact area, and impulse in novice ballet dancers	Arinli, Y; Umutlu, G; Pehlevan, Z	Web of Science	JOURNAL OF BACK AND MUSCULOSKELETAL REHABILITATION	2023	10.3233/BMR-220406	excluded	No comparison of arm movement in balance or postural control was conducted
744	Inhibitory response capacities of bilateral lower and upper extremities in children with developmental coordination disorder in endogenous and exogenous orienting modes	Tsai, CL; Yu, YK; Chen, YJ; Wu, SK	Web of Science	BRAIN AND COGNITION	2009	10.1016/j.bandc.2008.07.012	excluded	No comparison of arm movement in balance or postural control was conducted
745	Effects of a Combined Upper- and Lower-Limb Plyometric Training Program on High-Intensity Actions in Female U14 Handball Players	Hammami, M; Ramirez-Campillo, R; Gaamouri, N; Aloui, G; Shephard, RJ; Chelly, MS	Web of Science	PEDIATRIC EXERCISE SCIENCE	2019	10.1123/pes.2018-0278	excluded	No comparison of arm movement in balance or postural control was conducted

746	Simulating mechanical consequences of voluntary movement upon whole-body equilibrium: the arm-raising paradigm revisited	Pozzo, T; Ouamer, M; Gentil, C	Web of Science	BIOLOGICAL CYBERNETICS	2001	10.1007/PL00007995	excluded	computer simulation
747	Age-Related Reversal of Postural Adjustment Characteristics During Motor Imagery	Mitra, S; Doherty, N; Boulton, H; Maylor, EA	Web of Science	PSYCHOLOGY AND AGING	2016	10.1037/pag0000120	excluded	No comparison of arm movement in balance or postural control was conducted
748	Effects of the type and direction of support surface perturbation on postural responses	Chen, CL; Lou, SZ; Wu, HW; Wu, SK; Yeung, KT; Su, FC	Web of Science	JOURNAL OF NEUROENGINEERING AND REHABILITATION	2014	10.1186/1743-0003-11-50	excluded	No comparison of arm movement in balance or postural control was conducted
749	Peculiarities of Adolescent, Qualified Female Volleyball Players' Shoulder Girdle	Sakne, KE; Liepa, A; Pontaga, I	Web of Science	MLTJ-MUSCLES LIGAMENTS AND TENDONS JOURNAL	2024	10.32098/mltj.01.2024.14	excluded	No comparison of arm movement in balance or postural control was conducted
750	Temporal facilitation of gaze in the presence of postural reactions triggered by sudden surface perturbations	Paquette, C; Fung, J	Web of Science	NEUROSCIENCE	2007	10.1016/j.neuroscience.2006.12.027	excluded	No comparison of arm movement in balance or postural control was conducted

751	The effect of a shoulder injury prevention programme on proprioception and dynamic stability of young volleyball players; a randomized controlled trial	Zarei, M; Eshghi, S; Hosseinzadeh, M	Web of Science	BMC SPORTS SCIENCE MEDICINE AND REHABILITATION	2021	10.1186/s13102-021-00300-5	excluded	No comparison of arm movement in balance or postural control was conducted
752	A cutaneous positioning system	Martin, BJ; Lee, BC; Sienko, KH	Web of Science	EXPERIMENTAL BRAIN RESEARCH	2015	10.1007/s00221-014-4194-4	excluded	No comparison of arm movement in balance or postural control was conducted
753	Effectiveness of a Dry-Land Resistance Training Program on Strength, Power, and Swimming Performance in Paralympic Swimmers	Dingley, AA; Pyne, DB; Youngson, J; Burkett, B	Web of Science	JOURNAL OF STRENGTH AND CONDITIONING RESEARCH	2015	10.1519/JSC.0000000000000684	excluded	No comparison of arm movement in balance or postural control was conducted
754	Effects of initial foot position on postural responses to lateral standing surface perturbations in younger and older adults	Jeon, W; Griffin, L; Hsiao, HY	Web of Science	GAIT & POSTURE	2021	10.1016/j.gaitpost.2021.09.193	excluded	No comparison of arm movement in balance or postural control was conducted
755	Biomechanical constraints on the feedforward regulation of endpoint stiffness	Hu, X; Murray, WM; Perreault, EJ	Web of Science	JOURNAL OF NEUROPHYSIOLOGY	2012	10.1152/jn.00330.2012	excluded	No comparison of arm movement in balance or postural control was conducted
756	Physical characteristics of experienced and junior open-wheel car drivers	Raschner, C; Platzer, HP; Patterson, C	Web of Science	JOURNAL OF SPORTS SCIENCES	2013	10.1080/02640414.2012.720703	excluded	No comparison of arm movement in balance or postural control was conducted

757	A motor learning-based postural intervention with a robotic trunk support trainer to improve functional sitting in spinal cord injury: case report	Santamaria, V; Ai, X; Agrawal, SK	Web of Science	SPINAL CORD SERIES AND CASES	2022	10.1038/s41394-022-00554-2	excluded	No comparison of arm movement in balance or postural control was conducted
758	Neuromuscular performance of balance and posture control in childhood and adolescence	Ludwig, O; Kelm, J; Hammes, A; Schmitt, E; Fröhlich, M	Web of Science	HELIYON	2020	10.1016/j.heliyon.2020.e04541	excluded	No comparison of arm movement in balance or postural control was conducted
759	Improved shoulder stability through plyometric, proprioceptive and strength exercises in rugby players. A randomized clinical trial	Suarez-García, M; López-Mardomingo, P; Nah-Mohamed, M; Cuesta-Barriuso, R	Web of Science	JOURNAL OF MENS HEALTH	2021	10.31083/jomh.2021.021	excluded	No comparison of arm movement in balance or postural control was conducted
760	Scapular muscle endurance may improve shooting performance in air pistol shooters	Sezik, EG; Uysal, O; Sezik, AC; Duezguen, I	Web of Science	JOURNAL OF SPORTS MEDICINE AND PHYSICAL FITNESS	2023	10.23736/S0022-4707.23.14966-8	excluded	No comparison of arm movement in balance or postural control was conducted
761	Effect of an Injury Prevention Program on Traumatic Factors and Athletic Performance in juniors Judokas: the SLSTs Intervention	Mahmoudkhani, MR; Shakibaee, A; Minoonejad, H; Rajabi, R; Barati, AH	Web of Science	TRAUMA MONTHLY	2021	10.30491/TM.2021.214296.1045	excluded	No comparison of arm movement in balance or postural control was conducted

762	The effects of a community-centered muscle strengthening exercise program using an elastic band on the physical abilities and quality of life of the rural elderly	Park, SY; Kim, JK; Lee, SA	Web of Science	JOURNAL OF PHYSICAL THERAPY SCIENCE	2015	10.1589/jpts.27.2061	excluded	No comparison of arm movement in balance or postural control was conducted
763	How is precision regulated in maintaining trunk posture?	Willigenburg, NW; Kingma, I; van Dieën, JH	Web of Science	EXPERIMENTAL BRAIN RESEARCH	2010	10.1007/s00221-010-2207-5	excluded	No comparison of arm movement in balance or postural control was conducted
764	Analysis and control of a running spring-mass model with a trunk based on virtual pendulum concept	Karagoz, OK; Secer, G; Ankarali, MM; Saranli, U	Web of Science	BIOINSPIRATION & BIOMIMETICS	2022	10.1088/1748-3190/ac6d97	excluded	No comparison of arm movement in balance or postural control was conducted
765	Comparison of muscle strength imbalance in powerlifters and jumpers	Luk, HY; Winter, C; O'Neill, E; Thompson, BA	Web of Science	JOURNAL OF STRENGTH AND CONDITIONING RESEARCH	2014	10.1519/JSC.0b013e318295d311	excluded	No comparison of arm movement in balance or postural control was conducted
766	The Effects of Loaded Plyometrics and Short Sprints in U19 Male Soccer Players in Tunisia	Aloui, G; Souhail, H; Hayes, LD; Bouhafs, E; Chelly, MS; Schwesig, R	Web of Science	APPLIED SCIENCES-BASEL	2024	10.1371/journal.pone.0315103	excluded	No comparison of arm movement in balance or postural control was conducted
767	Effect of concentric exercise-induced fatigue on proprioception, motor control and performance of the upper limb in handball players	Hadjisavvas, S; Efstathiou, MA; Themistocleous, IC; Daskalaki, K; Malliou, P; Giannaki, CD; Lewis, J; Stefanakis, M	Web of Science	PLOS ONE	2021	10.3390/app11167621	excluded	No comparison of arm movement in balance or postural control was conducted

768	Head and Trunk Control While Walking in Older Adults with Diabetes: Effects of Balance Confidence	Hewston, P; Deshpande, N	Web of Science	JOURNAL OF MOTOR BEHAVIOR	2018	10.1080/00222895.2017.1283291	excluded	No comparison of arm movement in balance or postural control was conducted
769	Thorax and pelvis kinematics during walking, a comparison between children with and without cerebral palsy: A systematic review	Swinnen, E; Vander Goten, L; De Koster, B; Degelaen, M	Web of Science	NEUROREHABILITATION	2016	10.3233/NRE-161303	excluded	No comparison of arm movement in balance or postural control was conducted
770	Older adults utilize less efficient postural adaptations when they are uncertain about the magnitude of a perturbation	Kaewmanee, T; Liang, HQ; Madrid, KC; Aruin, AS	Web of Science	HUMAN MOVEMENT SCIENCE	2022	10.1016/j.humov.2022.102996	excluded	No comparison of arm movement in balance or postural control was conducted
771	The effect of Zumba exercises on body composition, dynamic balance and functional fitness parameters in 15-17 years old women with high body mass index	Kolayis, IE; Arol, P	Web of Science	PEDAGOGY OF PHYSICAL CULTURE AND SPORTS	2020	10.15561/26649837.2020.0303	excluded	No comparison of arm movement in balance or postural control was conducted
772	Self-Assisted Standing Enabled by Non-Invasive Spinal Stimulation after Spinal Cord Injury	Sayenko, DG; Rath, M; Ferguson, AR; Burdick, JW; Havton, LA; Edgerton, VR; Gerasimenko, YP	Web of Science	JOURNAL OF NEUROTRAUMA	2019	10.1089/neu.2018.5956	excluded	No comparison of arm movement in balance or postural control was conducted

773	5-week suspension training program increase physical performance of youth judokas: a pilot study	Norambuena, Y; Winkler, L; Guevara, R; Lavados, P; Monrroy, M; Ramírez-Campillo, R; Herrera-Valenzuela, T; Gajardo-Burgos, R	Web of Science	RETOS-NUEVAS TENDENCIAS EN EDUCACION FISICA DEPORTE Y RECREACION	2021		excluded	No comparison of arm movement in balance or postural control was conducted
774	10-week suspension and traditional push-up training: Comparison the effects on physical performance in young men	Bayrak, G; Aslan, UB	Web of Science	SCIENCE & SPORTS	2024	10.1016/j.scispo.2023.05.001	excluded	No comparison of arm movement in balance or postural control was conducted
775	Is the use of vestibular information weighted differently across the initiation of walking?	Bent, LR; McFadyen, BJ; Inglis, JT	Web of Science	EXPERIMENTAL BRAIN RESEARCH	2004	10.1007/s00221-004-1854-9	excluded	No comparison of arm movement in balance or postural control was conducted
776	Effects of strength training with elastic band programme on fitness components in young female handball players: a randomized controlled trial	Hammami, M; Gaamouri, N; Wagner, H; Pagaduan, JC; Hill, L; Nikolaidis, PT; Knechtle, B; Chelly, MS	Web of Science	BIOLOGY OF SPORT	2022	10.5114/biolsport.2022.106390	excluded	No comparison of arm movement in balance or postural control was conducted
777	Comparison of Path Length and Ranges of Movement of the Center of Pressure and Reaction Time and Between Paired-Play and Solo-Play of a Virtual Reality Game	Portnoy, S; Hersch, A; Sofer, T; Tresser, S	Web of Science	GAMES FOR HEALTH JOURNAL	2017	10.1089/g4h.2017.0017	excluded	No comparison of arm movement in balance or postural control was conducted

778	Arm raising in humans under loaded vs. unloaded and bipedal vs. unipedal conditions	Vernazza-Martin, S; Martin, N; Cincera, M; Pedotti, A; Massion, J	Web of Science	BRAIN RESEARCH	1999	10.1016/S0006-8993(99)01846-6	excluded	No comparison of arm movement in balance or postural control was conducted
779	Effect of movement speed on limb segment motions for reaching from a standing position	Thomas, JS; Corcos, DM; Hasan, Z	Web of Science	EXPERIMENTAL BRAIN RESEARCH	2003	10.1007/s00221-002-1287-2	excluded	No comparison of arm movement in balance or postural control was conducted
780	Effect of Reaction Time Exercises on Physical Functionality and Quality of Life in Geriatrics: A Non-controlled Study	Erdoganoglu, Y; Oktar, BE; Sel, S; Bodur, F	Web of Science	BEZMIALEM SCIENCE	2024	10.14235/bas.galenos.2023.43660	excluded	No comparison of arm movement in balance or postural control was conducted
781	Effects of Tai Chi on a Functional Arm Reaching Task in Older Adults: A Cross-Sectional Study	Varghese, R; Hui-Chan, CWY; Bhatt, T	Web of Science	JOURNAL OF AGING AND PHYSICAL ACTIVITY	2015	10.1123/japa.2014-0031	excluded	No comparison of arm movement in balance or postural control was conducted
782	The Effects of a Novel Quadrupedal Movement Training Program on Functional Movement, Range of Motion, Muscular Strength, and Endurance	Buxton, JD; Prins, PJ; Miller, MG; Moreno, A; Welton, GL; Atwell, AD; Talampas, TR; Elsey, GE	Web of Science	JOURNAL OF STRENGTH AND CONDITIONING RESEARCH	2022	10.1519/JSC.0000000000003818	excluded	No comparison of arm movement in balance or postural control was conducted

783	Effects of Eight-Week Circuit Training with Core Exercises on Performance in Adult Male Soccer Players	Belli, G; Marini, S; Mauro, M; Latessa, PM; Toselli, S	Web of Science	EUROPEAN JOURNAL OF INVESTIGATION IN HEALTH PSYCHOLOGY AND EDUCATION	2022	10.3390/ejihpe12090086	excluded	No comparison of arm movement in balance or postural control was conducted
784	Dynamic studies on human body sway by using a simple model with special concerns on the pelvic and muscle roles	Jiang, YF; Nagasaki, S; You, MS; Zhou, JL	Web of Science	ASIAN JOURNAL OF CONTROL	2006		excluded	No comparison of arm movement in balance or postural control was conducted
785	Stance width influences frontal plane balance responses to centripetal accelerations	Goodworth, A; Chandan, A; Chase, H; Foster, E; Francoeur, H; Michaud, J; Terry, K	Web of Science	GAIT & POSTURE	2013	10.1016/j.gaitpost.2012.06.019	excluded	No comparison of arm movement in balance or postural control was conducted
786	Motion analysis of throwing Boccia balls in children with cerebral palsy	Huang, PC; Pan, PJ; Ou, YC; Yu, YC; Tsai, YS	Web of Science	RESEARCH IN DEVELOPMENTAL DISABILITIES	2014	10.1016/j.ridd.2013.11.017	excluded	No comparison of arm movement in balance or postural control was conducted
787	High postural constraints affect the organization of reaching and grasping movements	Bourdin, C; Teasdale, N; Nougier, V	Web of Science	EXPERIMENTAL BRAIN RESEARCH	1998	10.1007/s002210050513	excluded	No comparison of arm movement in balance or postural control was conducted

788	Speed-related spinal excitation from ankle dorsiflexors to knee extensors during human walking	Iglesias, C; Nielsen, JB; Marchand-Pauvert, V	Web of Science	EXPERIMENTAL BRAIN RESEARCH	2008	10.1007/s00221-008-1344-6	excluded	No comparison of arm movement in balance or postural control was conducted
789	The strength of balance: Strength and dynamic balance in children with and without hypermobility	Ituen, OA; Duysens, J; Ferguson, G; Smits-Engelsman, B	Web of Science	PLOS ONE	2024	10.1371/journal.pone.0302218	excluded	No comparison of arm movement in balance or postural control was conducted
790	The Time Course of Health, Fitness, and Occupational Performance Changes in Recruits across a Fire Academy	Wohlgemuth, KJ; Gerstner, GR; Giuliani-Dewig, HK; Mota, JA; Smith-Ryan, AE; Ryan, ED	Web of Science	MEDICINE & SCIENCE IN SPORTS & EXERCISE	2023	10.1249/MSS.0000000000003119	excluded	No comparison of arm movement in balance or postural control was conducted
791	Comparing the effects of traditional resistance training and functional training on the bio-motor capacities of female elite taekwondo athletes	Khazaei, L; Parnow, A; Amani-shalamzari, S	Web of Science	BMC SPORTS SCIENCE MEDICINE AND REHABILITATION	2023	10.1186/s13102-023-00754-9	excluded	No comparison of arm movement in balance or postural control was conducted
792	Methodological infrastructure in surgical ergonomics: A review of tasks, models, and measurement systems	Lee, GS; Lee, T; Dexter, D; Klein, R; Park, A	Web of Science	SURGICAL INNOVATION	2007	10.1177/1553350607307956	excluded	No comparison of arm movement in balance or postural control was conducted
793	Effects of local and widespread muscle fatigue on movement timing	Cowley, JC; Dingwell, JB; Gates, DH	Web of Science	EXPERIMENTAL BRAIN RESEARCH	2014	10.1007/s00221-014-4020-z	excluded	No comparison of arm movement in balance or postural control was conducted

794	Effects of trunk muscle activation on trunk stability, arm power, blood pressure and performance in wheelchair rugby players with a spinal cord injury	Kouwijzer, I; van der Meer, M; Janssen, TWJ	Web of Science	JOURNAL OF SPINAL CORD MEDICINE	2022	10.1080/10790268.2020.1830249	excluded	No comparison of arm movement in balance or postural control was conducted
795	The Effect of Exergame Training on Physical Functioning of Healthy Older Adults: A Meta-Analysis	Hai, LG; Hou, HY; Zhou, C; Li, HJ	Web of Science	GAMES FOR HEALTH JOURNAL	2022	10.1089/g4h.2021.0173	excluded	No comparison of arm movement in balance or postural control was conducted
796	Lower extremity movement quality in professional team sport athletes: Inter-rater agreement and relationships with quantitative results from the corresponding pattern	Keller, M; Niederer, D; Schwesig, R; Kurz, E	Web of Science	BMC SPORTS SCIENCE MEDICINE AND REHABILITATION	2024	10.1186/s13102-024-00886-6	excluded	No comparison of arm movement in balance or postural control was conducted
797	Effects of singular and dual task constraints on motor skill variability in childhood	Gill, SV; Yang, Z; Hung, YC	Web of Science	GAIT & POSTURE	2017	10.1016/j.gaitpost.2017.01.021	excluded	No comparison of arm movement in balance or postural control was conducted
798	Motorized Mobility Scooters: The Use of Training/Intervention and Technology for Improving Driving Skills in Aging Adults - A Mini-Review	Toosizadeh, N; Bunting, M; Howe, C; Mohler, J; Sprinkle, J; Najafi, B	Web of Science	GERONTOLOGY	2014	10.1159/000356766	excluded	No comparison of arm movement in balance or postural control was conducted

799	Posture Control-Human-Inspired Approaches for Humanoid Robot Benchmarking: Conceptualizing Tests, Protocols and Analyses	Mergner, T; Lippi, V	Web of Science	FRONTIERS IN NEUROROBOTICS	2018	10.3389/fnbot.2018.00021	excluded	No comparison of arm movement in balance or postural control was conducted
800	Effects of an Offshore Sailing Competition on Anthropometry, Muscular Performance, Subjective Wellness, and Salivary Cortisol in Professional Sailors	Philippe, K; Paillard, T; Maurelli, O; Moody, J; Prioux, J	Web of Science	INTERNATIONAL JOURNAL OF SPORTS PHYSIOLOGY AND PERFORMANCE	2022	10.1123/ijspp.2021-0575	excluded	No comparison of arm movement in balance or postural control was conducted
801	Movement timing and reach to reach variability during a repetitive reaching task in persons with chronic neck/shoulder pain and healthy subjects	Lomond, KV; Côté, JN	Web of Science	EXPERIMENTAL BRAIN RESEARCH	2010	10.1007/s00221-010-2405-1	excluded	No comparison of arm movement in balance or postural control was conducted
802	Inferring Human Control Intent Using Inverse Linear Quadratic Regulator With Output Penalty Versus Gain Penalty: Better Fit but Similar Intent	Yu, H; Ramadan, A; Cholewicki, J; Popovich, JM ; Reeves, NP; You, JH; Choi, J	Web of Science	JOURNAL OF DYNAMIC SYSTEMS MEASUREMENT AND CONTROL-TRANSACTIONS OF THE ASME	2024	10.1115/1.4065593	excluded	No comparison of arm movement in balance or postural control was conducted

803	The influence of the reciprocal hip joint link in the Advanced Reciprocating Gait Orthosis on standing performance in paraplegia	Baardman, G; Ijzerman, MJ; Hermens, HJ; Veltink, PH; Boom, HBK; Zilvold, G	Web of Science	PROSTHETICS AND ORTHOTICS INTERNATIONAL	1997		excluded	No comparison of arm movement in balance or postural control was conducted
804	The Influence of Fatigue on Throwing and YBT-UQ Performance in Male Adolescent Handball Players	Bauer, J; Hagen, M; Weisz, N; Muehlbauer, T	Web of Science	FRONTIERS IN SPORTS AND ACTIVE LIVING	2020	10.3389/fspor.2020.00081	excluded	No comparison of arm movement in balance or postural control was conducted
805	Dual tasking affects lateral trunk control in healthy younger and older adults	Asai, T; Doi, T; Hirata, S; Ando, H	Web of Science	GAIT & POSTURE	2013	10.1016/j.gaitpost.2013.04.005	excluded	No comparison of arm movement in balance or postural control was conducted
806	Assessment of Dynamic Balancing Performance of Synchronized Ice Skaters With Sudden Provocation Test via Principal Component Analysis	Pálya, Z; Petró, B; Kiss, RM	Web of Science	JOURNAL OF MOTOR LEARNING AND DEVELOPMENT	2022	10.1123/jmid.2021-0059	excluded	No comparison of arm movement in balance or postural control was conducted
807	Physical activity and fitness in 8-year-old overweight and normal weight children and their parents	Karppanen, AK; Ahonen, SM; Tammelin, T; Vanhala, M; Korpelainen, R	Web of Science	INTERNATIONAL JOURNAL OF CIRCUMPOLAR HEALTH	2012	10.3402/ijch.v71i0.17621	excluded	No comparison of arm movement in balance or postural control was conducted

808	Effectiveness of Virtual Reality in Children With Cerebral Palsy: A Systematic Review and Meta-Analysis of Randomized Controlled Trials	Chen, YP; Fanchiang, HD; Howard, A	Web of Science	PHYSICAL THERAPY	2018	10.1093/ptj/pzx107	excluded	No comparison of arm movement in balance or postural control was conducted
809	The effectiveness of traditional and sling exercise strength training in women	Dannelly, BD; Otey, SC; Croy, T; Harrison, B; Rynders, CA; Hertel, JN; Weltman, A	Web of Science	JOURNAL OF STRENGTH AND CONDITIONING RESEARCH	2011	10.1519/JSC.0b013e318202e473	excluded	No comparison of arm movement in balance or postural control was conducted
810	Resistance exercise snacks improve muscle mass in female university employees: a prospective, controlled, intervention pilot-study	Brandt, T; Schwandner, CTL; Schmidt, A	Web of Science	FRONTIERS IN PUBLIC HEALTH	2024	10.3389/fpubh.2024.1347825	excluded	No comparison of arm movement in balance or postural control was conducted
811	Knee joint laxity and neuromuscular characteristics of male and female soccer and basketball players	Rozzi, SL; Lephart, SM; Gear, WS; Fu, FH	Web of Science	AMERICAN JOURNAL OF SPORTS MEDICINE	1999	10.1177/03635465990270030801	excluded	No comparison of arm movement in balance or postural control was conducted
812	Fitness testing in padel: Performance differences according to players' competitive level	Courel-Ibáñez, J; Herrera-Gálvez, JJ	Web of Science	SCIENCE & SPORTS	2020	10.1016/j.scispo.2019.05.009	excluded	No comparison of arm movement in balance or postural control was conducted

813	Gymnastic skills on a balance beam with simulated height	Ritter, Y; Bürger, D; Pastel, S; Sprich, M; Lück, T; Hacke, M; Stucke, C; Witte, K	Web of Science	HUMAN MOVEMENT SCIENCE	2023	10.1016/j.humov.2022.103023	excluded	No comparison of arm movement in balance or postural control was conducted
814	Variations induced by the use of unstable surface do not facilitate motor adaptation to a throwing skill	Moreno, FJ; Barbado, D; Caballero, C; Urban, T; Sabido, R	Web of Science	PEERJ	2023	10.7717/peerj.14434	excluded	No comparison of arm movement in balance or postural control was conducted
815	Dynamic balance abilities of collegiate men for the bench press	Piper, TJ; Radlo, SJ; Smith, TJ; Woodward, RW	Web of Science	JOURNAL OF STRENGTH AND CONDITIONING RESEARCH	2012	10.1519/JSC.0b013e318248d789	excluded	No comparison of arm movement in balance or postural control was conducted
816	Can Primary School Mathematics Performance Be Predicted by Longitudinal Changes in Physical Fitness and Activity Indicators?	Sember, V; Jurak, G; Starc, G; Morrison, SA	Web of Science	FRONTIERS IN PSYCHOLOGY	2022	10.3389/fpsyg.2022.796838	excluded	No comparison of arm movement in balance or postural control was conducted
817	Effects of Open Skill Visuomotor Choice Reaction Time Training on Unanticipated Jump-Landing Stability and Quality: A Randomized Controlled Trial	Friebe, D; Engeroff, T; Giesche, F; Niederer, D	Web of Science	FRONTIERS IN HUMAN NEUROSCIENCE	2021	10.3389/fnhum.2021.683909	excluded	No comparison of arm movement in balance or postural control was conducted

818	Does knee motion contribute to feet-in-place balance recovery?	Cheng, KYB	Web of Science	JOURNAL OF BIOMECHANICS	2016	10.1016/j.jbiomech.2016.04.026	excluded	No comparison of arm movement in balance or postural control was conducted
819	Influence of Bilateral Vestibular Loss on Spinal Stabilization in Humans	Goodworth, AD; Peterka, RJ	Web of Science	JOURNAL OF NEUROPHYSIOLOGY	2010	10.1152/jn.01064.2009	excluded	No comparison of arm movement in balance or postural control was conducted
820	Effects of Otago exercise program on physical function in older adults: A systematic review and meta-analysis of randomized controlled trials	Wu, S; Guo, YZ; Cao, Z; Nan, JH; Zhang, QX; Hu, MY; Ning, HT; Huang, WP; Xiao, LD; Feng, H	Web of Science	ARCHIVES OF GERONTOLOGY AND GERIATRICS	2024	10.1016/j.archger.2024.105470	excluded	No comparison of arm movement in balance or postural control was conducted
821	Is the attenuation effect on the ankle muscles activity from the EMG biofeedback generalized to-or compensated by-other lower limb muscles during standing?	dos Anjos, FV; Pinto, TP; Cerone, GL; Gazzoni, M; Vieira, TM	Web of Science	JOURNAL OF ELECTROMYOGRAPHY AND KINESIOLOGY	2022	10.1016/j.jelekin.2022.102721	excluded	No comparison of arm movement in balance or postural control was conducted
822	Evidence-based approach to physical therapy in cerebral palsy	Das, SP; Ganesh, GS	Web of Science	INDIAN JOURNAL OF ORTHOPAEDICS	2019	10.4103/ortho.IJOrtho_241_17	excluded	No comparison of arm movement in balance or postural control was conducted
823	Multi-Task Center-of-Pressure Metrics Estimation With Graph Convolutional Network	Du, C; Graham, S; Depp, C; Nguyen, T	Web of Science	IEEE TRANSACTIONS ON MULTIMEDIA	2022	10.1109/TMM.2021.3075025	excluded	No comparison of arm movement in balance or postural control was conducted

824	Why is the explicit component of motor adaptation limited in elderly adults?	Vandevoorde, K; de Xivry, JJO	Web of Science	JOURNAL OF NEUROPHY SIOLOGY	2020	10.1152/jn.00659 .2019	excluded	No comparison of arm movement in balance or postural control was conducted
825	Study of between-subject and within-subject variability of electromyography data and its intrinsic determinants for clip fitting tasks	Gaudez, C; Wild, P; Gilles, MA; Savin, J; Claudon, L; Bailleul, D	Web of Science	INTERNATIO NAL JOURNAL OF OCCUPATIO NAL SAFETY AND ERGONOMI CS	2021	10.1080/1080354 8.2019.1568754	excluded	No comparison of arm movement in balance or postural control was conducted
826	The role of the sensorimotor system in the athletic shoulder	Myers, JB; Lephart, SM	Web of Science	JOURNAL OF ATHLETIC TRAINING	2000		excluded	No comparison of arm movement in balance or postural control was conducted
827	The professional network underlying cerebral palsy intervention research based on systematic reviews and meta-analyses published in international journals: authors? communities, institutional networks, and international collaboration	Pinter, H; Gal, F; Molnar, P	Web of Science	HELIYON	2022	10.1016/j.heliyon .2022.e09718	excluded	No comparison of arm movement in balance or postural control was conducted
828	Spiritual Intelligence: A Vital Component of Consolation within Rehabilitation for Medical Social Workers	Nilsson H.	Web of Science	Soc Work Health Care	2024	10.1080/0098138 9.2024.2425611	excluded	No comparison of arm movement in balance or postural control was conducted

829	A Pilot Evaluation of mHealth App Accessibility for Three Top-Rated Weight Management Apps by People with Disabilities	Radcliffe E, Lippincott B, Anderson R, Jones M.	Web of Science	Int J Environ Res Public Health	2021	10.3390/ijerph18073669	excluded	No comparison of arm movement in balance or postural control was conducted
830	A qualitative study to explore the barriers and enablers for young people with disabilities to access sexual and reproductive health services in Senegal	Burke E, Kébé F, Flink I, van Reeuwijk M, le May A.	Web of Science	Reprod Health Matters	2017	10.1080/09688080.2017.1329607	excluded	No comparison of arm movement in balance or postural control was conducted
831	The impact of disability-related deprivation on employment opportunity at the neighborhood level: does family socioeconomic status matter?	Qiu N, Jiang Y, Sun Z, Du M.	Web of Science	Front Public Health	2023	10.3389/fpubh.2023.1232829	excluded	No comparison of arm movement in balance or postural control was conducted
832	Racial and Ethnic Differences in Disability Transitions Among Older Adults in the United States	Dong L, Freedman VA, Sánchez BN, Mendes de Leon CF.	Web of Science	J Gerontol A Biol Sci Med Sci	2019	10.1093/gerona/gly052	excluded	No comparison of arm movement in balance or postural control was conducted
833	Barriers to the long-term recovery of individuals with disabilities following a disaster	Stough LM, Sharp AN, Resch JA, Decker C, Wilker N.	Web of Science	Disasters	2016	10.1111/disa.12161	excluded	No comparison of arm movement in balance or postural control was conducted
834	Assessment of Accommodation Requests Reported by a National Sample of US MD Students by Category of Disability	Meeks LM, Pereira-Lima K, Plegue M, Stergiopoulos E, Jain NR, Addams A, Moreland CJ.	Web of Science	JAMA	2022	10.1001/jama.2022.12283	excluded	No comparison of arm movement in balance or postural control was conducted

835	The meaning of adapted ice-skating for children and youths with disabilities	Thorslund E, Rosberg S.	Web of Science	Disabil Rehabil	2024	10.1080/09638288.2024.2317998	excluded	No comparison of arm movement in balance or postural control was conducted
836	Working "With" Not "On" Disabled People: The Role of Hate Crime Research within the Community	Burch L.	Web of Science	J Interpers Violence	2024	10.1177/08862605241260005	excluded	No comparison of arm movement in balance or postural control was conducted
837	Cocreating guide dog partnerships: dog training and interdependence in 1930s America	Pemberton N.	Web of Science	Med Humanit	2019	10.1136/medhum·2018-011626	excluded	No comparison of arm movement in balance or postural control was conducted
838	Performing Pain and Inflammation: Rendering the Invisible Visible	Dokumaci A.	Web of Science	AMA J Ethics	2017	10.1001/journalofethics.2017.19.8.imhl1-1708	excluded	No comparison of arm movement in balance or postural control was conducted
839	Evaluation of the Impact of the First Wave of COVID-19 and Associated Lockdown Restrictions on Persons with Disabilities in 14 States of India	Tetali S, Kamalakannan S, Sadanand S, Lewis MG, Varughese S, Hans A, Murthy GVS.	Web of Science	Int J Environ Res Public Health	2022	10.3390/ijerph191811373	excluded	No comparison of arm movement in balance or postural control was conducted
840	Return to work factors and vocational rehabilitation interventions for long-term, partially disabled workers: a modified Delphi study among vocational rehabilitation professionals	de Geus CJC, Huysmans MA, van Rijssen HJ, Anema JR.	Web of Science	BMC Public Health	2022	10.1186/s12889-022-13295-6	excluded	No comparison of arm movement in balance or postural control was conducted

841	Data-Driven Smart Living Lab to Promote Participation in Rehabilitation Exercises and Sports Programs for People with Disabilities in Local Communities	Lee SB, Oh YT, Yang SW, Kim JB.	Web of Science	Sensors (Basel)	2023	10.3390/s23052761	excluded	No comparison of arm movement in balance or postural control was conducted
842	Perceptions of people living with Parkinson's disease: a qualitative study in Iran	Soleimani MA, Bastani F, Negarandeh R, Greysen R.	Web of Science	Br J Community Nurs	2016	10.12968/bjcn.2016.21.4.188	excluded	No comparison of arm movement in balance or postural control was conducted
843	Pregnancy in Disability: Community Perceptions and Personal Experiences in a Rural Setting in Ghana	Akasreku BD, Habib H, Ankomah A.	Web of Science	J Pregnancy	2018	10.1155/2018/8096839	excluded	No comparison of arm movement in balance or postural control was conducted
844	Visual function of children with visual and other disabilities in Oman: A case series	Gogri U, Khandekar R, Al Harby S.	Web of Science	Indian J Ophthalmol	2016	10.4103/0301-4738.198845	excluded	No comparison of arm movement in balance or postural control was conducted
845	The shadow side of occupational therapy: Necropower, state racism and colonialism	Turcotte PL, Holmes D.	Web of Science	Scand J Occup Ther	2024	10.1080/11038128.2023.2264330	excluded	No comparison of arm movement in balance or postural control was conducted
846	A longitudinal study on self-rated health changes in disabled older people	Yi E, Choi B.	Web of Science	Front Public Health	2024	10.3389/fpubh.2024.1372463	excluded	No comparison of arm movement in balance or postural control was conducted
847	Employment Legal Framework for Persons with Disabilities in China: Effectiveness and Reasons	Hao Y, Li P.	Web of Science	Int J Environ Res Public Health	2020	10.3390/ijerph17144976	excluded	No comparison of arm movement in balance or postural control was conducted

848	Impact of rehabilitation services on employment outcomes for individuals with physical disabilities: a propensity score matching analysis	Park HN, Lee SJ, Yoon JY.	Web of Science	BMC Public Health	2024	10.1186/s12889-024-19015-6	excluded	No comparison of arm movement in balance or postural control was conducted
849	The medical reshaping of disabled bodies as a response to stigma and a route to normality	McLaughlin J.	Web of Science	Med Humanit	2017	10.1136/medhum-2016-011065	excluded	No comparison of arm movement in balance or postural control was conducted
850	Self-reported sexual coercion among in-school young people with disabilities in Ghana	Seidu AA, Kumi-Kyereme A, Darteh EKM.	Web of Science	BMC Public Health	2024	10.1186/s12889-024-18631-6	excluded	No comparison of arm movement in balance or postural control was conducted
851	Trajectories and characteristics of functional impairment before and after suicide attempt in young adults - a nationwide register-based cohort study	Wang M, Helgesson M, Rahman S, Niederkrotenthaler T, Mittendorfer-Rutz E.	Web of Science	BMC Psychiatry	2017	10.1186/s12888-017-1567-9	excluded	No comparison of arm movement in balance or postural control was conducted
852	Description of an interdisciplinary, holistic cognitive rehabilitation program for adults with mild to moderate cognitive impairment after acquired brain injury	Reilly KT, Holé J, Nash S, Pugniet V, Servajean V, Varsovie D, Jacquin-Courtois S.	Web of Science	Disabil Rehabil	2024	10.1080/09638288.2022.2157058	excluded	No comparison of arm movement in balance or postural control was conducted

853	Barriers and Facilitators to Accessing Health Services: A Qualitative Study Amongst People with Disabilities in Cameroon and India	Zuurmond M, Mactaggart I, Kannuri N, Murthy G, Oye JE, Polack S.	Web of Science	Int J Environ Res Public Health	2019	10.3390/ijerph16071126	excluded	No comparison of arm movement in balance or postural control was conducted
854	Chronic Musculoskeletal Disabilities following Snake Envenoming in Sri Lanka: A Population-Based Study	Jayawardana S, Gnanathasan A, Arambepola C, Chang T.	Web of Science	PLoS Negl Trop Dis	2016	10.1371/journal.pntd.0005103	excluded	No comparison of arm movement in balance or postural control was conducted
855	Usability of World Health Organization Disability Assessment Schedule in chronic traumatic brain injury	Tarvonen-Schröder S, Tenovuo O, Kaljonen A, Laimi K.	Web of Science	J Rehabil Med	2018	10.2340/16501977-2345	excluded	No comparison of arm movement in balance or postural control was conducted
856	More Than Just Assistive Devices: How a South African Social Enterprise Supports an Environment of Inclusion	Trafford Z, van der Westhuizen E, McDonald S, Linegar M, Swartz L.	Web of Science	Int J Environ Res Public Health	2021	10.3390/ijerph18052655	excluded	No comparison of arm movement in balance or postural control was conducted
857	Being disabled' as an exclusion criterion for clinical trials: a scoping review	Camanni G, Ciccone O, Lepri A, Tinarelli C, Bedetti C, Cicuttin S, Murgia N, Elisei S.	Web of Science	BMJ Glob Health	2023	10.1136/bmjgh-2023-013473	excluded	No comparison of arm movement in balance or postural control was conducted
858	Peer support needs, preferences and experiences of adults with acquired neurological disability: a scoping review protocol	Wellecke C, Douglas J, Winkler D, Brown M.	Web of Science	BMJ Open	2024	10.1136/bmjopen-2024-088237	excluded	No comparison of arm movement in balance or postural control was conducted

859	Investigating physical activity levels in adults who are blind and vision impaired	Flynn L, Millar K, Belton S, O'Connor N, Meegan S, Britton U, Behan S.	Web of Science	Disabil Health J	2024	10.1016/j.dhjo.2024.101594	excluded	No comparison of arm movement in balance or postural control was conducted
860	Diversity & Inclusion: Fitness for Work Through a Personalized Work Plan For Workers With Disabilities and Chronic Diseases According to the Iso 30415/2021	Cristaudo A, Guglielmi G, Foddis R, Caldi F, Buselli R, Brilli C, Coggiola M, Ceccarelli G, Saffioti G.	Web of Science	Med Lav	2023	10.23749/mdl.v114i3.14339	excluded	No comparison of arm movement in balance or postural control was conducted
861	Secondary health conditions and social role satisfaction in adults with long-term physical disability	Battalio SL, Jensen MP, Molton IR.	Web of Science	Health Psychol	2019	10.1037/hea0000671	excluded	No comparison of arm movement in balance or postural control was conducted
862	The incidence of depression among residents of assisted living: prevalence and related risk factors	Almomani FM, Bani-Issa W.	Web of Science	Clin Interv Aging	2017	10.2147/CIA.S147436	excluded	No comparison of arm movement in balance or postural control was conducted
863	National approaches to promote sports and physical activity in adults with disabilities: examples from the Netherlands and Canada	Hoekstra F, Roberts L, van Lindert C, Martin Ginis KA, van der Woude LHV, McColl MA.	Web of Science	Disabil Rehabil	2019	10.1080/09638288.2017.1423402	excluded	No comparison of arm movement in balance or postural control was conducted
864	Communication access on trains: a qualitative exploration of the perspectives of passengers with communication disabilities	Bigby C, Johnson H, O'Halloran R, Douglas J, West D, Bould E.	Web of Science	Disabil Rehabil	2019	10.1080/09638288.2017.1380721	excluded	No comparison of arm movement in balance or postural control was conducted

865	Limited accessibility to HIV services for persons with disabilities living with HIV in Ghana, Uganda and Zambia	Tun W, Okal J, Schenk K, Esantsi S, Mutale F, Kyeremaa RK, Ngirabakunzi E, Asiah H, McClain-Nhlapo C, Moono G.	Web of Science	J Int AIDS Soc	2016	10.7448/IAS.19.5.20829	excluded	No comparison of arm movement in balance or postural control was conducted
866	Determinants of caregiver burden of persons with disabilities in a rural district in Egypt	Ghazawy ER, Mohammed ES, Mahfouz EM, Abdelrehim MG.	Web of Science	BMC Public Health	2020	10.1186/s12889-020-09266-4	excluded	No comparison of arm movement in balance or postural control was conducted
867	What is the gap in activity and participation between people with disability and the general population in Taiwan?	Chiu TY, Yen CF, Escorpizo R, Chi WC, Liou TH, Liao HF, Chou CH, Fang WH.	Web of Science	Int J Equity Health	2017	10.1186/s12939-017-0628-5	excluded	No comparison of arm movement in balance or postural control was conducted
868	Disability and its impact on life expectancy: heterogeneity across Mexican states	Baptista EA, Shen T, Canudas-Romo V.	Web of Science	BMC Public Health	2024	10.1186/s12889-024-20245-x	excluded	No comparison of arm movement in balance or postural control was conducted
869	Assessment of Disclosure of Psychological Disability Among US Medical Students	Meeks LM, Plegue M, Case B, Swenor BK, Sen S.	Web of Science	JAMA Netw Open	2020	10.1001/jamanetworkopen.2020.11165	excluded	No comparison of arm movement in balance or postural control was conducted
870	The International Classification of Functioning, Disability and Health (ICF) core sets for deafblindness, part II of the systematic review: linking data to the ICF categories	Jaiswal A, Paramasivam A, Budhiraja S, Santhakumaran P, Gravel C, Martin J, Ogedengbe TO, James TG, Kennedy B, Tang D, Tran Y, Colson-Osborne H, Minhas R, Granberg S, Wittich W.	Web of Science	Eur J Phys Rehabil Med	2024	10.23736/S1973-9087.24.07984-X	excluded	No comparison of arm movement in balance or postural control was conducted

871	Inflammation in multimorbidity and disability: An integrative review	Friedman E, Shorey C.	Web of Science	Health Psychol	2019	10.1037/hea0000749	excluded	No comparison of arm movement in balance or postural control was conducted
872	Requirements on a community-based intervention for stimulating physical activity in physically disabled people: a focus group study amongst experts	Krops LA, Hols DHJ, Folkertsma N, Dijkstra PU, Geertzen JHB, Dekker R.	Web of Science	Disabil Rehabil	2018	10.1080/09638288.2017.1336645	excluded	No comparison of arm movement in balance or postural control was conducted
873	Accessibility and inclusiveness of new information and communication technologies for disabled users and content creators in the Metaverse	Radanliev P, De Roure D, Novitzky P, Sluganovic I.	Web of Science	Disabil Rehabil Assist Technol	2024	10.1080/17483107.2023.2241882	excluded	No comparison of arm movement in balance or postural control was conducted
874	Disabled-by-design: effects of inaccessible urban public spaces on users of mobility assistive devices - a systematic review	Kapsalis E, Jaeger N, Hale J.	Web of Science	Disabil Rehabil Assist Technol	2024	10.1080/17483107.2022.2111723	excluded	No comparison of arm movement in balance or postural control was conducted
875	Collaboration in providing intimate-partner violence services to women with disabilities	Namatovu F, Ineland J.	Web of Science	BMC Public Health	2024	10.1186/s12889-024-19352-6	excluded	No comparison of arm movement in balance or postural control was conducted
876	Engagement in Meaningful Activities Among Older Adults With Disability, Dementia, and Depression	Oh A, Gan S, Boscardin WJ, Allison TA, Barnes DE, Covinsky KE, Smith AK.	Web of Science	JAMA Intern Med	2021	10.1001/jamainternmed.2020.7492	excluded	No comparison of arm movement in balance or postural control was conducted

877	Disability Rights During COVID-19: Emergency Law and Guidelines in England	Antova I.	Web of Science	Med Law Rev	2020	10.1093/medlaw/fwaa026	excluded	No comparison of arm movement in balance or postural control was conducted
878	Nonsense variant in a consanguineous family expands the phenotype of KPTN gene-related syndrome to include hearing impairment	Liaqat K, Bharadwaj T, Shah K, Nasir A, Acharya A, Khan S, Ullah I, Schrauwen I, Ahmad W, Leal SM.	Web of Science	Clin Genet	2023	10.1111/cge.14390	excluded	No comparison of arm movement in balance or postural control was conducted
879	Developing additional competition classes for athletes with intellectual impairments: Conceptual approach and efficacy of an ICF derived measure	Lemmey S, Burns J, Jones F.	Web of Science	J Sports Sci	2021	10.1080/02640414.2021.1881302	excluded	No comparison of arm movement in balance or postural control was conducted
880	Participation of adults with cognitive, physical, or psychiatric impairments in family of origin and intimate relationships: a grounded theory study	Pfister A, Georgi-Tscherry P, Berger F, Studer M.	Web of Science	BMC Public Health	2020	10.1186/s12889-020-08770-x	excluded	No comparison of arm movement in balance or postural control was conducted
881	National Institutes of Health Designates Disabled People a Health Disparity Population	Reynolds JM.	Web of Science	JAMA Health Forum	2024	10.1001/jamahealthforum.2024.1185	excluded	No comparison of arm movement in balance or postural control was conducted
882	Sex/gender disparities in health outcomes of individuals with long-term disabling conditions	Thakral M, Lacroix AZ, Molton IR.	Web of Science	Rehabil Psychol	2019	10.1037/rep0000248	excluded	No comparison of arm movement in balance or postural control was conducted

883	Differences in the Progression of Disability: A U.S.-Mexico Comparison	Díaz-Venegas C, Reistetter TA, Wong R.	Web of Science	J Gerontol B Psychol Sci Soc Sci	2018	10.1093/geronb/gbw082	excluded	No comparison of arm movement in balance or postural control was conducted
884	Burden of disability in children and adolescents must be integrated into the global health agenda	Cieza A, Kamenov K, Sanchez MG, Chatterji S, Balasegaram M, Lincetto O, Servili C, Bermejo R, Ross DA.	Web of Science	BMJ	2021	10.1136/bmj.n9	excluded	No comparison of arm movement in balance or postural control was conducted
885	LidSonic V2.0: A LiDAR and Deep-Learning-Based Green Assistive Edge Device to Enhance Mobility for the Visually Impaired	Busaeed S, Katib I, Albeshri A, Corchado JM, Yigitcanlar T, Mehmood R.	Web of Science	Sensors (Basel)	2022	10.3390/s22197435	excluded	No comparison of arm movement in balance or postural control was conducted
886	Leprosy and lymphatic filariasis-related disability and psychosocial burden in northern Mozambique	van Wijk R, Raimundo L, Nicala D, Stakteas Y, Cumbane A, Muquingue H, Cliff J, van Brakel W, Muloliwa AM.	Web of Science	PLoS Negl Trop Dis	2024	10.1371/journal.pntd.0012342	excluded	No comparison of arm movement in balance or postural control was conducted
887	Adverse health effects of climate change and air pollution in people with disabilities: a systematic review	Rhim N, Lee S, Choi KH.	Web of Science	Epidemiol Health	2024	10.4178/epih.e2024080	excluded	No comparison of arm movement in balance or postural control was conducted
888	Multidimensional poverty of persons with disabilities in China: An analysis of poverty reduction effect of employment services	Wang X, Guo J, Li H.	Web of Science	Front Public Health	2023	10.3389/fpubh.2023.1093978	excluded	No comparison of arm movement in balance or postural control was conducted

889	Development of a New Negative Obstacle Sensor for Augmented Electric Wheelchair	Favey C, Farcy R, Donnez J, Villanueva J, Zogaghi A.	Web of Science	Sensors (Basel)	2021	10.3390/s21196341	excluded	No comparison of arm movement in balance or postural control was conducted
890	The Implementation of the Convention on the Rights of Persons with Disabilities: More Than Just Another Reform of Psychiatry	Russo J, Wooley S.	Web of Science	Health Hum Rights	2020		excluded	No comparison of arm movement in balance or postural control was conducted
891	Disabled women's attendance at community women's groups in rural Nepal	Morrison J, Colbourn T, Budhathoki B, Sen A, Adhikari D, Bamjan J, Pathak S, Basnet A, Trani JF, Costello A, Manandhar D, Groce N.	Web of Science	Health Promot Int	2017	10.1093/heapro/dav099	excluded	No comparison of arm movement in balance or postural control was conducted
892	A qualitative exploration of Chinese rural older adults' adaption experience to disability in Henan Province	Gao M, Zhang Y, Tian Y, Gao Y, Li X, Lu Y.	Web of Science	BMC Public Health	2023	10.1186/s12889-023-15425-0	excluded	No comparison of arm movement in balance or postural control was conducted
893	Life expectancy and healthy life expectancy of Korean registered disabled by disability type in 2014-2018: Korea National Rehabilitation Center database	Jang H, Choi KH, Kim JA, Choi YJ.	Web of Science	BMC Public Health	2023	10.1186/s12889-023-16682-9	excluded	No comparison of arm movement in balance or postural control was conducted
894	Toward a new definition of rehabilitation for research purposes: a comparative analysis of current definitions	Meyer T, Kiekens C, Selb M, Posthumus E, Negrini S.	Web of Science	Eur J Phys Rehabil Med	2020	10.23736/S1973-9087.20.06610-1	excluded	No comparison of arm movement in balance or postural control was conducted

895	Responsiveness of people with moderate and significant intellectual disability to physical stimulation	Ślężyńska M, Mięsok G, Mięsok K.	Web of Science	Ann Agric Environ Med	2018	10.5604/12321966.1233560	excluded	No comparison of arm movement in balance or postural control was conducted
896	Health knowledge and the impact of social exclusion on young people with intellectual disabilities	Pownall J, Wilson S, Jahoda A.	Web of Science	J Appl Res Intellect Disabil	2020	10.1111/jar.12331	excluded	No comparison of arm movement in balance or postural control was conducted
897	Effect of the age of visual impairment onset on employment outcomes in South Korea: analysis of the national survey on persons with disabilities data	Jeon B, Koo H, Lee HJ, Han E.	Web of Science	BMC Public Health	2022	10.1186/s12889-022-13747-z	excluded	No comparison of arm movement in balance or postural control was conducted
898	Gesture controlled human-computer interface for the disabled	Szczepaniak OM, Sawicki DJ.	Web of Science	Med Pr	2017	10.13075/mp.5893.00529	excluded	No comparison of arm movement in balance or postural control was conducted
899	Late life disability and experienced wellbeing: Are economic resources a buffer?	Freedman VA, Cornman JC, Carr D, Lucas RE.	Web of Science	Disabil Health J	2019	10.1016/j.dhjo.2019.02.003	excluded	No comparison of arm movement in balance or postural control was conducted
900	Essential components of rehabilitation services provided to visually impaired people	Sarabandi A, Vatankhah S, Kamali M, Aryankhesal A.	Web of Science	Clin Exp Optom	2021	10.1111/cxo.13121	excluded	No comparison of arm movement in balance or postural control was conducted
901	Assistive Technology's Potential to Improve Employment of People with Disabilities	Kruse D, Schur L, Johnson-Marcus HA, Gilbert L, Di Lallo A, Gao W, Su H.	Web of Science	J Occup Rehabil	2024	10.1007/s10926-023-10164-w	excluded	No comparison of arm movement in balance or postural control was conducted

902	Improving a web-based employability intervention for work-disabled employees: results of a pilot economic evaluation	Noben C, Evers S, Genabeek JV, Nijhuis F, de Rijk A.	Web of Science	Disabil Rehabil Assist Technol	2017	10.3109/17483107.2015.1135999	excluded	No comparison of arm movement in balance or postural control was conducted
903	Types and severity of physical impairments of para taekwondo athletes	Davalli A, O'Sullivan DM, Bella S, Jeong HS.	Web of Science	J Sports Med Phys Fitness	2021	10.23736/S0022-4707.21.12675-1	excluded	No comparison of arm movement in balance or postural control was conducted
904	Disabilities and medical emergencies: a population-based study in Peru	Jiménez-Lozada MA, López-Magallanes LD, Alarco JJ.	Web of Science	Emergencias	2024	10.55633/s3me/040.2024	excluded	No comparison of arm movement in balance or postural control was conducted
905	Physical activity experiences of community-dwelling older adults with physical disabilities: a scoping review of qualitative research	Jackman PC, Cooke S, George T, Blackwell J, Middleton G.	Web of Science	Disabil Rehabil	2024	10.1080/09638288.2023.2253536	excluded	No comparison of arm movement in balance or postural control was conducted
906	Participation and quality of life outcomes among individuals with earthquake-related physical disability: A systematic review	Nunnerley J, Dunn J, McPherson K, Hooper G, Woodfield T.	Web of Science	J Rehabil Med	2015	10.2340/16501977-1965	excluded	No comparison of arm movement in balance or postural control was conducted
907	Disability Trajectories at the End of Life: A "Countdown" Model	Wolf DA, Freedman VA, Ondrich JI, Seplaki CL, Spillman BC.	Web of Science	J Gerontol B Psychol Sci Soc Sci	2015	10.1093/geronb/gbu182	excluded	No comparison of arm movement in balance or postural control was conducted
908	Attitudes of Students of Social Sciences and Humanities towards People with Physical Disabilities (MAS-PL)	Tomczyszyn D, Pańczuk A, Szepeluk A.	Web of Science	Int J Environ Res Public Health	2022	10.3390/ijerph19031544	excluded	No comparison of arm movement in balance or postural control was conducted

909	Confronting the Legacy of Eugenics and Ableism: Towards Anti-Ableist Bioscience Education	Da Silva SM, Hubbard K.	Web of Science	CBE Life Sci Educ	2024	10.1187/cbe.23-10-0195	excluded	No comparison of arm movement in balance or postural control was conducted
910	**Clinical Implications of Hand Position and Lower Limb Length Measurement Method on Y-Balance Test Scores and Interpretations**	**Hébert-Losier K.**		**J Athl Train**	**2017**	**10.4085/1062-6050-52.8.02**	**included**	
911	**Effect of Lower Limb Muscle Fatigue on Dynamic Balance Performance in Healthy Young Adults: Role of Arm Movement**	**Borgmann, Katharina & Brinkmann, Ruben & Bauer, Julian & Hill, Matt & Muehlbauer, Thomas.**		**Sports Medicine Internationa l Open**	**2024**	**10.1055/a-2346-2759.**	**included**	
912	**Exploring how arm movement moderates the effect of lower limb muscle fatigue on dynamic balance in healthy youth**	**Borgmann K, Ferdenhert J, Neyses AC, Bauer J, Hill MW and Muehlbauer T**		**Front Sports Act Living**	**2025**	**10.3389/fspor.2024.1391869**	**included**	
913	**Age, Cognitive Task, and Arm Position Differently Affect Muscle Synergy Recruitment but have Similar Effects on Walking Balance.**	**da Silva Costa AA, Hortobágyi T, den Otter R, Sawers A, Moraes R.**		**Neuroscien ce**	**2023**	**10.1016/j.neuroscience.2023.07.010**	**included**	
914	**Beam width and arm position but not cognitive task affect walking balance in older adults**	**da Silva Costa AA, Hortobágyi T, Otter RD, Sawers A, Moraes R.**		Sci Rep	**2022**	**10.1038/s41598-022-10848-y**	**included**	

915	Effects of Soccer Exercise on Balance Ability and Kinesthesia of the Lower Limb Joints in Children Aged 5-6 Years	Shen K, Liu Y.	PubMed	Motor Control	2022	10.1123/mc.2021-0093	excluded	No comparison of arm movement in balance or postural control was conducted
916	Influence of controlled masticatory muscle activity on dynamic reactive balance	Fadillioglu C, Kanus L, Möhler F, Ringhof S, Schindler HJ, Stein T, Hellmann D.	PubMed	J Oral Rehabil	2022	10.1111/joor.13284	excluded	No comparison of arm movement in balance or postural control was conducted
917	Community-based postural control assessment in autistic individuals indicates a similar but delayed trajectory compared to neurotypical individuals	Fears NE, Sherrod GMC, Templin TN, Bugnariu NL, Patterson RM, Miller HL.	PubMed	Autism Res	2023	10.1002/aur.2889	excluded	No comparison of arm movement in balance or postural control was conducted
918	Center of pressure position in the touches of the star excursion balance test in healthy individuals	Petter GDN, Pereira FF, Glänzel MH, da Silva FS, Pozzobon D, Saccol MF, Mota CB.	PubMed	J Bodyw Mov Ther	2022	10.1016/j.jbmt.2022.02.012	excluded	No comparison of arm movement in balance or postural control was conducted
919	Altered characteristics of balance control in obese older adults	Melzer I, Oddsson LI.	PubMed	Obes Res Clin Pract	2016	10.1016/j.orcp.2015.05.016	excluded	No comparison of arm movement in balance or postural control was conducted
920	Relationships among the Y balance test, Berg Balance Scale, and lower limb strength in middle-aged and older females	Lee DK, Kang MH, Lee TS, Oh JS.	PubMed	Braz J Phys Ther	2015	10.1590/bjpt-rbf.2014.0096	excluded	No comparison of arm movement in balance or postural control was conducted
921	Postural control patterns in gravid women-A systematic review	Forczek-Karkosz W, Masłoń A.	PubMed	PLoS One	2024	10.1371/journal.pone.0312868	excluded	No comparison of arm movement in balance or postural control was conducted
